# Machine learning-based approach for identification of new resistance associated mutations from whole genome sequences of *Mycobacterium tuberculosis*

**DOI:** 10.1093/bioadv/vbaf050

**Published:** 2025-03-11

**Authors:** Ankita Pal, Debasisa Mohanty

**Affiliations:** Bioinformatics Center, National Institute of Immunology, New Delhi 110067, India; Bioinformatics Center, National Institute of Immunology, New Delhi 110067, India

## Abstract

**Motivation:**

Currently available methods for the prediction of genotypic drug resistance in *Mycobacterium tuberculosis* utilize information on known markers of drug resistance. Hence, machine learning approaches are needed that can discover new resistance markers.

**Results:**

Whole genome sequences with known phenotypic drug resistance profiles have been utilized to train XGBoost and ANN classifiers for 5 first-line and 8 second-line tuberculosis drugs. Benchmarking on a completely independent dataset from CRyPTIC database revealed that our method has high sensitivity (90%–95%) and specificity (94%–99%) for five first-line drugs and robust performance for six second-line drugs with a sensitivity of 77%–89% at over 95% specificity. An explainable AI method, SHapley Additive exPlanations, has successfully identified resistance mutations for each drug in a completely automated way. This approach could not only identify known resistance associated mutations in agreement with the WHO mutation catalogue, but also predicted >100 other potential resistance associated mutations for 13 antibiotics in new genes outside the known resistance loci. Identification of new resistance markers opens up the opportunity for the discovery of novel mechanisms of drug resistance.

**Availability and implementation:**

Our prediction method has been implemented as TB-AMRpred webserver and command line tool, available freely at http://www.nii.ac.in/TB-AMRpred.html and https://github.com/Ankitapal1995/TB-AMRprd.

## 1 Introduction

The rapid increase in the incidence of drug resistant (DR) tuberculosis (TB) has emerged as a major global health challenge (Goletti *et al.* 2025). In recent years, there has been a shift toward genotypic Drug Susceptibility Testing (DST) as an alternative to the time-consuming phenotypic DST for *Mycobacterium tuberculosis* (*M.tb*) strains, allowing for rapid prediction of drug sensitivity based on genomic mutations. This approach utilizes advances in genome sequencing technology to quickly analyze the bacterial genome from sputum samples (Nimmo *et al.* 2019), providing a faster and resource-efficient method compared to traditional culture-based DST. Hence, results of genotypic DST can be available within a few days in contrast to phenotypic DST which takes several weeks. The recent recommendations of WHO on the use of genotypic DST (WHO 2024) as an alternative to phenotypic DST have opened up the opportunity for fighting drug-resistant *M.tb* globally. Hence, in recent years the development of computational methods for the accurate prediction of drug resistance from the genome sequence of *M.tb* has been a topic of active research.

The decreasing cost of whole genome sequence (WGS) has resulted in the availability of WGSs of a large number of mycobacterium strains with known phenotypic DST profiles in public databases like BV-BRC (previously known as PATRIC) (Antonopoulos *et al.* 2019), NCBI (Sayers *et al.* 2023), ReSeqTB (Ezewudo *et al.* 2018), CRyPTIC (Allix-Béguec *et al*. 2018) and WHO tuberculosis mutation catalogue (Walker *et al.* 2022). These sequences of susceptible and resistant *M.tb* strains have facilitated the identification of DR associated mutations for different antibiotics by comparative genome analysis. Resistance in *M.tb* results from mutations, insertion/deletions (indels), and infrequent structural variations. Unlike other pathogenic bacteria, drug resistance phenotypes in *M.tb* are primarily determined by mutations that commonly occur within genes encoding primary drug targets or regulatory genes, such as *katG, inhA, ahpC, rpoB, rpoC, embB, rrs, rpsL*, and *pncA*, (Coll *et al.* 2014, Galagan 2014, Merker *et al.* 2015, Oppong *et al.* 2019). Therefore, a large majority of antibiotic resistance prediction tools such as TB-Profiler (Coll *et al.* 2015), Mykrobe (Hunt *et al.* 2019), KVarQ (Steiner *et al.* 2014), MTBseq (Kohl *et al.* 2018), ReSeqTB-UVP (Ezewudo *et al.* 2018), SAM-TB (Yang *et al.* 2022), PhyResSE (Feuerriegel *et al.* 2015) have been built by using mutations in these known resistance associated genes as their primary knowledge base. Recent findings have highlighted limitations in the 2021 WHO mutation catalogue's predictive accuracy (Walker *et al.* 2022), emphasizing the necessity to explore additional drug resistance mutations to enhance predictive precision.

Since the WGS of resistance strains harbors mutations governing unknown antibiotic resistance mechanisms (Boolchandani *et al.* 2019), focusing on regions outside known markers of drug resistance can potentially reveal novel resistance associated mutations in both coding and noncoding regions (Farhat *et al.* 2013, Phelan *et al.* 2016, Coll *et al.* 2018, Sobkowiak *et al.* 2020). Few studies have revealed that there are likely hidden mechanisms involving alternative genes that contribute to resistance (Kavvas *et al.* 2018, Cohen *et al.* 2019, Poulton and Rock 2022). Machine learning (ML) based methods for prediction of genotypic DR for tuberculosis have shown high sensitivities >90% (Kouchaki *et al.* 2019, Jamal *et al.* 2020, Kuang *et al.* 2022). Recent machine learning based tools such as GenTB (Gröschel *et al.* 2021) and MTB-CNN (Green *et al.* 2022) have been able to predict new mutations but only in known resistance-associated genomic regions. Because these methods rely on 18 pre-defined genomic regions associated with resistance, failing to identify novel resistance genes. To overcome this, new ML approaches are needed that can discover previously unknown resistance markers and mechanisms across diverse *M.tb* strains.

In this work, we have developed a novel machine learning based methodology for identifying highly relevant mutations across the entire genome of *M.tb* utilizing explainable AI (XAI) without relying on the current knowledge of drug resistance biomarkers. This approach significantly enhances the detection of correlations between individual mutations and drug resistance associations, irrespective of their prevalence within resistant and susceptible *M.tb* populations. Through the training of 13 distinct drug resistance prediction models encompassing SNPs (Single nucleotide polymorphism) and INDELs (Insertion and Deletions) within the complete genomes of 13 947 *M.tb* isolates, our study has yielded promising results. Moreover, by using an XAI framework, such as SHAP to score individual mutations across the whole genome based on their discriminative importance between two classes: drug-resistant and drug-susceptible. After the model is trained for optimal classification of resistant and susceptible genomes, the contribution of each mutation to the overall classification in terms of their SHAP and XGBoost feature importance scores have been calculated. Mutations with high SHAP or XGBoost feature importance scores are predicted as resistance associated mutations. We have also carried out a comprehensive evaluation of DR associated mutations predicted by our method with the WHO mutation catalogue to demonstrate that our XAI framework can successfully identify a significant number of resistance associated mutations in the WHO catalogue, even though our method does not utilize known mutations for training. A most important contribution of our method is the prediction of a significant number of DR associated mutations in new genes as well as new mutations in known resistance associated genes. For many of these new mutations, we have been able to provide a rationale for the mechanistic basis of their association with drug resistance.

## 2 Methods

**Figure 1.**
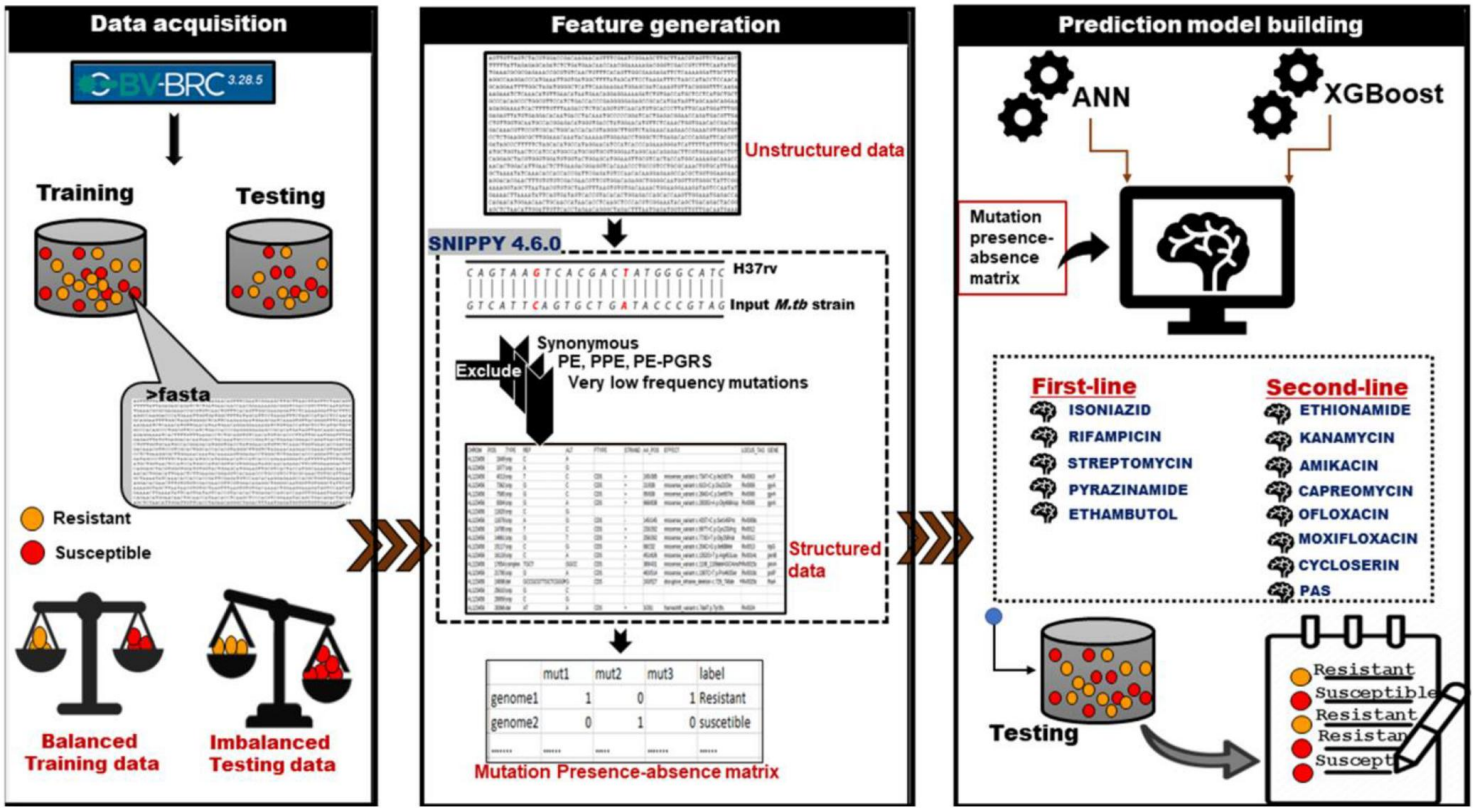
Flowchart depicting various steps involved in building machine learning models for drug resistance prediction.

Figure 1 depicts a schematic overview of the methodology for building the machine learning models for prediction of genotypic DR. It involves compilation of WGS and associated phenotypic DST data, generation of feature vectors, building the machine learning models, and their benchmarking. Details on each of these steps are described below.

### 2.1 Compilation of WGS of *M.tb* strains with known antibiotic resistance phenotype data

A total of 13 947 whole genome *fasta* sequences from the BV-BRC [previously known as PATRIC database (Antonopoulos *et al.* 2019)] were used to train 13 different resistance prediction models including first-line and second-line drugs (Table 1), whereas a separate set of 12 602 sequences were used to test (Table 2) those models (Supplementary Methods).

**Table 1.** Training dataset: a total of 13 947 *M.tb* isolates taken from BV-BRC were used for 13 individual antibiotic resistance model building.^a^

Drug type	Drugs	No. of resistance	No. of susceptible
First-line	Isoniazid (INH)	3512	3512
	Ethambutol (EMB)	1700	1700
	Rifampicin (RIF)	2750	2750
	Pyrazinamide (PZA)	1500	1500
	Streptomycin (STM)	1500	1500
Second-line	Ethionamide (ETH)	1076	1076
	Ofloxacin (OFLX)	959	959
	Kanamycin (KAN)	500	500
	Capreomycin (CAP)	400	400
	Amikacin (AMI)	350	350
	Moxifloxacin (MXF)	242	242
	Cycloserine (CYCLO)	100	100
	Para-Aminosalicylic Acid (PAS)	90	90

a An equal number of resistant and susceptible isolates were taken to build an unbiased prediction model.

b Note that since each genome can exhibit multiple drug resistances, the total genome count does not sum to 13 947.

**Table 2.** Test dataset: a total of 12602 *M.tb* isolates taken from BV-BRC, were used for every 13 drug-resistant prediction model evaluations.^a^

Drug type	Drugs	No. of resistance	No. of susceptible
First-line	Isoniazid (INH)	288	3186
	Ethambutol (EMB)	200	3642
	Rifampicin (RIF)	278	5178
	Pyrazinamide (PZA)	230	1796
	Streptomycin (STM)	235	916
Second-line	Ethionamide (ETH)	63	1036
	Ofloxacin (OFLX)	100	890
	Kanamycin (KAN)	38	740
	Capreomycin (CAP)	38	587
	Amikacin (AMI)	62	596
	Moxifloxacin (MXF)	35	831
	Cycloserine (CYCLO)	26	362
	Para-Aminosalicylic Acid (PAS)	10	283

a Note that not all the isolates were tested for every drug, hence the counts per drug do not add up to 12602.

### 2.2 Generation of feature vectors for machine learning

Each *M.tb* WGS was aligned with a reference sequence *M.tb* H37Rv (Genome assembly ID: GCA_000195955.2, biosample ID: SAMEA3138326) using SNIPPY^4.6.0^ (Seemann) and all SNPs and INDELs in coding and noncoding regions were gathered (Supplementary Methods). Mutation files were pre-processed as outlined in Supplementary Fig. S1 to exclude *PE* (Proline-Glutamic acid), *PPE* (Proline-Proline-Glutamic acid), *PE-PGRS* (Proline-Glutamic acid Repeat Sequence) regions (Gómez-González *et al.* 2023). The mutations of all the DR *M.tb* strains were taken as features (as shown in Supplementary Table S1) to create the presence/absence binary matrix for each *M.tb* genome. This matrix was used as feature vector to build the model using *scikit-learn^1.1.1^* (Pedregosa *et al.* 2011) framework.

### 2.3 Building the machine learning models

Whole genome extreme gradient boost (WG-XGB) and Whole genome artificial neural network (WG-ANN) models were built for all 13 drugs. WG-ANN models were implemented with *Keras* (v3.0.1) (Chollet 2015) upon *Tensorflow* (v2.10) (Abadi *et al.* 2016). The WG-XGB models were trained with a “gpu_hist” tree method, random_state of 1, learning_rate of 0.1, and other parameters with the default values of the current release of XGBoost (XGBoost^1.7.1)^) (Friedman 2001). Both the WG-XGB and WG-ANN models developed in the current study as well as the TB-Profiler were benchmarked on the same dataset (BV-BRC and CRyPTIC) by taking their DST labels as gold standard. Performances of the models were evaluated using standard statistical measures namely Recall/sensitivity (Sn), specificity (Sp), Matthews Correlation Coefficient (MCC), Precision/Positive Predictive Value (PPV), Negative Predictive Value (NPV), F1 Score, and Accuracy (Supplementary Methods).

### 2.4 Benchmarking of the ML models on BV-BRC & CRyPTIC datasets

The WG-XGB and WG-ANN models were benchmarked on a hold out test datasets consisting of 12 602 isolates (as shown in Table 2) as well as a completely independent CRyPTIC dataset (CRyPTIC *et al.* 2018) consisting of 12 287 genomes with matched DST profiles. The evaluation was based on the Recall/sensitivity (Sn), specificity (Sp), MCC, Precision/PPV, NPV, F1 Score, and Accuracy values with actual DST data serving as the ground truth (Supplementary Methods).

### 2.5 Feature importance analysis for identification of DR associated mutations

Feature importance scores for each mutation in the XGBoost model were evaluated using XGBoost^1.7.1^ inbuilt functions, while for WG-ANN model, for extracting important feature information, we implemented XAI using SHAP^0.41.0^ (Shapley Additive exPlanations) framework (Supplementary Methods). We combined the normalized scores from both XGBoost’s internal feature module and SHAP to create a unified list of important mutations or resistance associated mutations (Supplementary Fig. S2).

### 2.6 Comparison of predicted mutations with WHO mutation catalogue

Our ML-predicted mutations were compared based on the five categories listed in the WHO mutation catalogue 2021 (Walker *et al.* 2022) such as (i) “Assoc w R,” (ii) “Assoc w R-interim,” (iii) “Uncertain significance,” (iv) “Not assoc w R,” and (v) “Not assoc w R-interim.” The mutations have been assigned to different categories based on odds ratio (OR) and *P*-value of their occurrence in the resistant and susceptible genomes. Mutations labeled as “Assoc w R” have high statistical significance for their association with DR, mutations labeled “Assoc w R-interim” and “Uncertain Significance” do not meet the required significance cut-off for association with drug resistance. The details on OR and *P*-value cut-off for various types of mutations are described in the Supplementary Methodology Section (Supplementary Methods). Any new mutation not reported yet but occurring in known resistance associated regions was named “New mutation in known gene,” whereas mutations appearing in entirely new genes with unreported involvement were named “New mutations.” Mutations associated with resistance in other drugs were termed co-occurrent mutations.

### 2.7 Implementation as a webserver

We have implemented our WG-XGB based method as a webserver named as “TB-AMRpred” on a LINUX machine using HTML, CSS, Javascript, python flask framework, and Apache webserver.

## 3 Results

### 3.1 Training of ML classifiers and n-fold cross validation

During the training and cross-validation (CV) of the WG-XGB and WG-ANN classifiers, the models were optimized using 2-fold, 5-fold, 10-fold, and leave-one-out (LOO) approach. For all the five first-line drugs the specificity (sp) values were in the range of 99%–100%. For all four methods of CV, the sensitivities (sn) varied from 78% to 94% in case of 2-fold cross validation, which corresponded to an equal partition of training and test data. The sensitivity values increased for 5- and 10-fold CV with the increase in the size of training data and LOO yielded the best result for these five first-line drugs with sensitivities in the range of 80%–95% (Supplementary Table S2). For all the second-line drugs except ETH, the specificity varied from 90% to 100% for all four different CV methods, but only three of the second-line drugs (AMI, MXF, and OFLX) had sensitivity values above 80% in all 4 methods of CV. KAN and CAP had sensitivities of 47.6% and 69.5% in cases of 2-fold CV, while sensitivities for CYCLO and PAS varied from 12% to 64% for different CV methods. In case of ETH, the LOO yielded a sensitivity of 78% at a specificity of 79%, while 2-fold CV had a sensitivity of 70% at a specificity of 79%, indicating higher false positive rates for ETH compared to other drugs. Thus CV results indicated that using our WG-XGB and WG-ANN classifiers genotypic DR can be predicted with reasonably high sensitivities at minimal false positive rate for 10 out of the 13 drugs.

### 3.2 Benchmarking of the ML models on BV-BRC test dataset

The performance of the WG-XGB and WG-ANN models as well as TB-Profiler were also evaluated on the hold out test dataset (Table 2). As can be seen from Table 3, for the five first-line drugs both of our ML models and TB-Profiler have high specificity in the range of 94%–100% and sensitivity of 86%–95%, indicating high prediction accuracy even on unseen data. The sensitivity of WG-XGB and WG-ANN for EMB and RIF are comparable to the sensitivity of TB-Profiler, while for STM and PZA, WG-XGB shows higher sensitivity compared to WG-ANN and TB-Profiler. In case of INH, the sensitivity of WG-ANN and TB-Profiler was higher than the sensitivity of WG-XGB. For the second-line drugs except ETH, WG-XGB, and TB-Profiler showed high specificity in the range of 90%–100% and the specificity of the WG-XGB model was either better or comparable to the sensitivity of TB-Profiler (Table 3). On the other hand, WG-ANN model showed lower specificity values, i.e. 68%, 55%, 79% for ETH, OFLX, MXF, respectively. For KAN, AMI, CAP, OFLX, and MXF, WG-XGB model showed sensitivities above 77%–89% even at very high specificity values and they were either better or comparable to the sensitivity of TB-Profiler. For MXF, KAN, and OFLX, WG-ANN model had specificity values of 79%, 88%, and 55%, respectively, indicating poor performance compared to WG-XGB and TB-Profiler (Table 3). It is important to note that for newer drugs like CYCLO and PAS, WG-XGB model showed sensitivities of 54% and 64%, respectively, while TB-Profiler showed very lower sensitivity in the range of 27%–30%. WG-ANN model had a sensitivity of 54% for PAS, but only 27% for CYCLO (Table 3). In case of ETH, all three models had specificities in the range of 68%–77%. However, the sensitivity of WG-XGB for ETH was 77% which is higher than the sensitivities of TB-Profiler (68%) and WG-ANN (49%). Since the sensitivity of a given model can change with a change in specificity, we also compared the performance of WG-XGB, WG-ANN, and TB-Profiler based on their MCC values and F1 scores for predictions on this hold-out dataset (Table 3). As can be seen, in terms of MCC value for INH, PZA, STM, AMI, CYCLO, and PAS, WG-XGB models performed bettethan TB-Profiler, while for the other drugs like EMB, RIF, KAN and ETH performance of WG-XGB and TB-profiler were similar. However, for OFLX and MXF, TB-profiler performed better than WG-XGB. The MCC values for WG-ANN were lower compared to WG-XGB for all 13 drugs and lower compared to TB-Profiler for all drugs except PAS. Although the sample sizes for PAS and CYCLO were smaller than those for other drugs (Table 2), it is worth noting that the WG-XGB models predicted outcomes with higher MCC and F1 score values compared to TB-Profiler. Supplementary Table S3 shows precision (PPV), NPV, and accuracy values of WG-XGB, WG-ANN, and TB-profiler for predictions on this test dataset. Since our BV-BRC hold out test dataset was imbalanced with susceptible samples outnumbering the resistant counterparts, we used a downsampling approach to create a balanced dataset for evaluating model performance. This approach preserved the total number of minority class (DR) instances while randomly selecting an equivalent number of majority class (drug susceptible) samples to create a proportionally representative dataset. We compared model performance on the balanced dataset with the results from imbalanced test datasets in terms of sensitivity (recall), specificity, MCC, precision (PPV), NPV, F1 score, and accuracy (Supplementary Table S4). Interestingly, that sensitivity remained consistent across balanced and imbalanced datasets, as it is independent of negative sample distribution. Specificity demonstrated slight variations (1%–3%) for both WG-XGB and WG-ANN models, with notable exceptions in the ETH dataset. In the imbalanced test dataset of ETH, specificity was 77%, whereas the balanced test dataset showed 68%. Notably, other performance metrics improved significantly with dataset balancing for both WG-XGB and WG-ANN, particularly for the ETH strain. For example, the MCC value for ETH exhibited substantial enhancement, increasing from 0.29 to 0.48 for WG-XGB and from 0.08 to 0.14 for WG-ANN when transitioning from imbalanced to balanced datasets.

**Table 3.** Model evaluation on BV-BRC test dataset containing 12 602 *M.tb* isolates using Sn (sensitivity), Sp (specificity), MCC (Matthews Correlation Coefficient), and F1 score.^a^

Drugs		INH	EMB	RIF	PZA	STM	ETH	OFLX	KAN	CAP	AMI	MXF	CYCLO	PAS
R		288	200	278	230	235	63	100	38	38	62	35	26	11
S		3186	3642	5178	1796	916	1036	890	740	587	596	831	362	283
WG-XGB	Sn	86%	90%	90%	**95%**	**91%**	**79%**	**89%**	79%	**89%**	77%	83%	**54%**	**64%**
WG-ANN		90%	**92%**	**93%**	81%	83%	49%	52%	65%	87%	73%	72%	27%	54%
TB-profiler		94%	90%	91%	87%	82%	68%	87%	86%	78%	80%	86%	27%	30%
WG-XGB	Sp	**98%**	99%	97%	99%	99%	77%	96%	**99%**	100%	**99%**	96%	90%	97%
WG-ANN		94%	97%	92%	95%	99%	68%	55%	88%	98%	90%	79%	92%	98%
TB-profiler		94%	99%	97%	100%	99%	82%	99%	97%	100%	97%	99%	96%	99%
WG-XGB	MCC	**0.81**	**0.91**	0.74	**0.94**	**0.94**	0.29	0.77	**0.78**	**0.94**	**0.81**	0.52	**0.32**	**0.52**
WG-ANN		0.69	0.74	0.57	0.71	0.86	0.08	0.04	0.34	0.78	0.50	0.24	0.16	0.48
TB-profiler		0.73	0.85	0.75	0.91	0.85	0.29	0.88	0.71	0.88	0.77	0.81	0.26	0.37
WG-XGB	F1 score	**0.83**	**0.91**	**0.75**	**0.95**	**0.95**	0.27	0.78	**0.87**	**0.94**	**0.85**	0.58	**0.38**	**0.54**
WG-ANN		0.70	0.75	0.55	0.73	0.90	0.14	0.18	0.33	0.84	0.54	0.21	0.22	0.52
TB-profiler		0.72	0.86	0.74	0.93	0.88	0.29	0.88	0.70	0.88	0.77	0.81	0.29	0.37

a The bold-highlighted numbers demonstrate the superior performance of our model compared to the TB-profiler.

### 3.3 Prediction on CRyPTIC data

In order to benchmark our models on a larger independent dataset we used genomes with known phenotypic DST profiles from CRyPTIC dataset after ensuring that they had no overlap with our training data. Out of the 13 antibiotics for which we had developed ML models, CRyPTIC dataset had WGS linked DST profiles for only three first-line drugs (INH, EMB, and RIF) and four second-line drugs (KAN, AMI, MXF, and ETH). Table 4 shows the number of resistant and susceptible samples in the CRyPTIC dataset and sensitivities, specificities, MCC, F1 scores values of WG-XGB, WG-ANN, and TB-profiler. While precision (PPV), NPV and accuracy have been shown in Supplementary Table S5. It can be seen from Table 4 that WG-XGB model demonstrated sensitivities of 84%, 91%, and 92% corresponding to the specificity values of 96%, 90%, and 95% for the first-line drugs INH, EMB, and RIF, respectively. WG-ANN model also showed very similar sensitivity and specificity values for these three first-line drugs. TB-Profiler showed sensitivities of 92%, 92%, and 94% corresponding to specificities of 97%, 92%, and 94% for INH, EMB, and RIF, respectively. Interestingly, even on the much larger independent dataset both WG-XGB and WG-ANN models show comparable performance to TB-Profiler, though for INH, TB-Profiler has 8% higher sensitivity compared to our ML models at similar specificity. Since the number of resistance samples is close to 6000, our models give around 48 more false negative predictions compared to TB-Profiler. WG-XGB model achieved sensitivities of 72%, 74%, 83%, and 72% corresponding to specificities of 93%, 99%, 84%, and 92% for the second-line drugs ETH, KAN, AMI, and MXF, respectively. For these four second-line drugs, TB-Profiler had sensitivities in the range of 70%–85% corresponding to specificities of 94%–98%. The WG-ANN model, on the other hand, performed did not perform well for second-line drugs (sensitivities 36%–68%; specificities 55%–93%). These results indicate that, while WG-XGB model showed comparable performance to TB-Profiler for both first and second-line drugs even on the large independent test dataset like CRyPTIC, WG-ANN showed poor performance for second line drugs. To further assess the overall performance comparison of WG-XGB, WG-ANN, and TB-profiler, we calculated MCC values. The MCC for the WG-XGB model was comparable to the corresponding values for TB-Profiler for all antibiotics except AMI and MXF. In the case of AMI, WG-XGB shows an MCC value of 0.80 compared to 0.74 for TB-profiler. For MXF, the WG-XGB had MCC of 0.53 compared to 0.73 for TB-Profiler, because it showed 10% lower specificity compared to TB-Profiler. The WG-ANN model had poor MCC values for all 4 second-line drugs, while its MCC for first-line drugs was comparable to WG-XGB and TB-Profiler. A similar trend was observed for the F1 score as well. The performances of WG-XGB and WG-ANN were also compared using a balanced CRyPTIC dataset obtained by using a downsampling approach. It was noted that sensitivity remained unchanged (Supplementary Table S6), while specificity was observed to vary by 1%–3% across most drugs, with notable exceptions for INH, ETH, and MXF. For INH, specificity was reduced from 96% to 84% for WG-XGB and from 91% to 85% for WG-ANN when transitioning from imbalanced to balanced datasets. In the case of ETH, the specificity of the balanced dataset was increased from 55% to 76% for WG-ANN, consequently leading to improvements in other parameters including MCC, precision, and accuracy. In case of MXF, specificities for the balanced dataset were 97% and 82% for WG-XGB and WG-ANN, respectively, while the corresponding values for the imbalanced dataset were 84% and 57%, respectively (Supplementary Table S6).

**Table 4.** Model evaluation on CRyPTIC dataset containing 12 287 *M.tb* isolates using Sn (sensitivity), Sp (specificity), MCC (Matthews Correlation Coefficient), and F1 score.^a^

Drugs		INH	EMB	RIF	ETH	KAN	AMI	MXF
R		5908	2261	4684	1727	1121	883	1724
S		6161	8337	7414	9431	11 008	11 188	10 565
WG-XGB	Sn	84%	91%	92%	72%	**72%**	74%	83%
WG-ANN		86%	92%	94%	43%	51%	36%	68%
TB-profiler		92%	92%	94%	74%	70%	76%	85%
WG-XGB	Sp	96%	90%	**95%**	93%	93%	**99%**	84%
WG-ANN		91%	92%	90%	55%	93%	94%	57%
TB-profiler		97%	92%	94%	95%	96%	98%	94%
WG-XGB	MCC	0.81	0.74	0.87	0.61	0.54	**0.80**	0.53
WG-ANN		0.76	0.78	0.83	0.01	0.40	0.26	0.17
TB-profiler		0.89	0.78	0.88	0.68	0.63	0.74	0.73
WG-XGB	F1 score	0.90	0.80	0.92	0.68	0.58	**0.81**	0.60
WG-ANN		0.88	0.83	0.90	0.22	0.46	0.35	0.32
TB-profiler		0.94	0.83	0.92	0.74	0.67	0.75	0.76

a The bold-highlighted numbers demonstrate the superior performance of our model compared to the TB-profiler method.

A recently Id method MTB++ Investigated drug resistance prediction for *M.tb* using a novel approach utilizing unique 31-mer features extracted from *de novo* assembled whole genomes of 6224 *M.tb* isolates from the CRyPTIC dataset (Serajian *et al.* 2024). LASSO Logistic Regression (LR) and Random Forest (RF) algorithms were used to train their predictive models. Comparison of results from MTB++ with other tools like TB-profiler, ResFinder, MyKrobe, and KvarQ demonstrated its superior performance. For first-line drugs, MTB++ achieved a mean F1 score of 96, 95, and 86 for INH, RIF, and EMB, respectively on hold out CRyPTIC dataset, while our best performing WG-XGB model had F1 scores of 90, 92, and 80 for INH, RIF, and EMB, respectively. The better performance of MTB++ compared to our ML method could arise either from the use of different ML algorithms like LASSO LR and RF or because of training using a portion of CRyPTIC dataset, unlike our method which does not use any CRyPTIC data for training.

### 3.4 Resistance associated mutations predicted by XAI and comparison with WHO mutation catalogue

**Figure 2.**
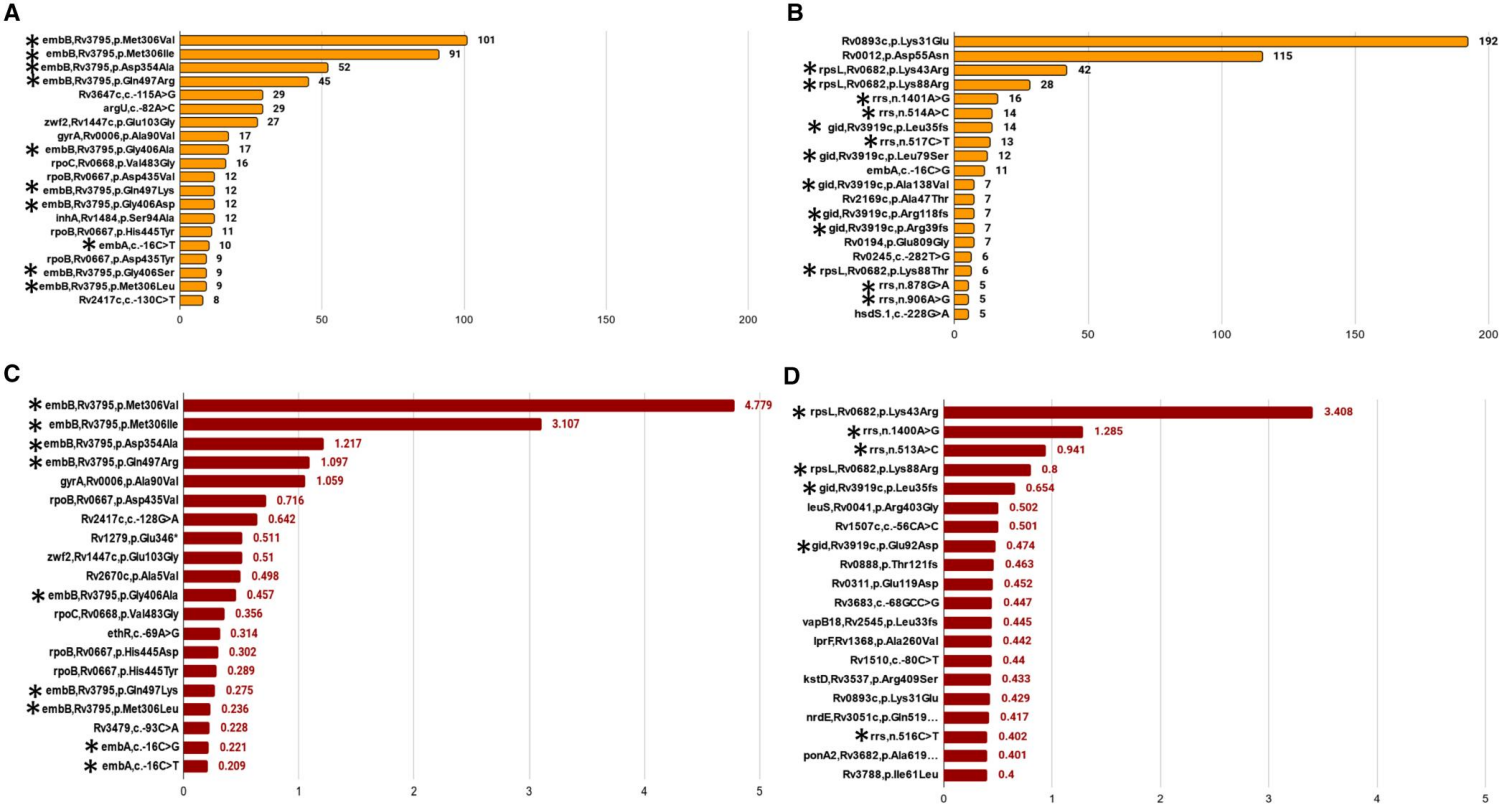
Identification of mutations associated with drug resistance by explainable AI (XAI) approach. Feature scoring was performed utilizing XAI techniques. Specifically, XGBoost internal feature importance module was used for WG-XGB models (A and B), while SHAP values were utilized for WG-ANN models (C and D) to evaluate the contribution of each mutation to drug response. The top 20 mutations with the highest feature importance scores were identified for drugs. (A) EMB and (B) STM using XGBoost, and for drugs. (C) EMB and (D) STM using SHAP values. Among these, those marked with an asterisk (*) represent known DR associated mutations, while the remaining mutations are novel DR-associated mutations.

Unlike other computational methods for prediction of genotypic DR which use only known marker regions for prediction, the WG-XGB and WG-ANN models use the entire sequence of the genome barring PE/PPE regions. One of the objectives of the current study was to investigate if our method can correctly identify known resistance associated mutations as well as novel mutations in known genes or new genes. As described in the methods section, we attempted to identify resistance associated mutations based on the identification of features that contribute maximally to discriminate the resistant versus susceptible genomes or by XAI using SHAP^0.41.0^ (Shapley Additive exPlanations) framework. Figure 2 shows the feature importance scores for the WG-XGB model and SHAP scores for the WG-ANN model for the top 20 mutations for EMB and STM. As can be seen in addition to known DR-associated mutations for these two antibiotics, several new mutations also have high feature importance or SHAP scores. Supplementary Table S7 shows the number of DR associated mutations for different antibiotics as predicted by our ML models. Since both WG-XGB and WG-ANN methods had a significant overlap in the predicted resistance associated mutations, the union of predictions by both methods was considered as putative DR associated mutations. As WG-ANN model exhibited suboptimal performance for genotypic DR prediction in case of OFLX, MXF, and ETH in comparison to other methods, for these three drugs only DR associated mutations predicted by WG-XGB models were utilized. We evaluated the accuracy of our resistance associated mutation prediction by comparing our results with WHO mutation catalogue published in 2021 (first edition) and 2023 (latest edition) as ground truth. Supplementary Table S8 shows the five different categories of mutations as per the “final confidence grading” of WHO for each antibiotic. The DR associated mutations predicted by our XAI approach were compared with the list of mutations in each of the categories in WHO catalogue. Supplementary Table S8 also shows the number of predicted mutations that overlap with each category. It is worth noting that our predicted mutation list has no overlap with “Not Assoc with R” and “Not Assoc w R-Interim” and we also predict a very significant number of mutations from the “Assoc w R” category. Out of the thousands of mutations listed in the “Uncertain Significance” category, our method predicts a very small number of mutations, which could be genuinely associated with DR. Similarly, out of hundreds of mutations listed by WHO in “Assoc w R-Interim,” we predict a very small number as associated with DR. These results demonstrate the success of our feature importance score and SHAP score in identifying true DR associated mutations. Since WHO lists only mutations in the known marker regions (Supplementary Table S9), our predicted mutations which do not overlap with any of the categories of WHO list could be categorized as new mutations in known genes or mutations in new genes. As can be seen, our method has identified several mutations in new genes and a few new mutations in known genes (Table 5).

**Figure 3.**
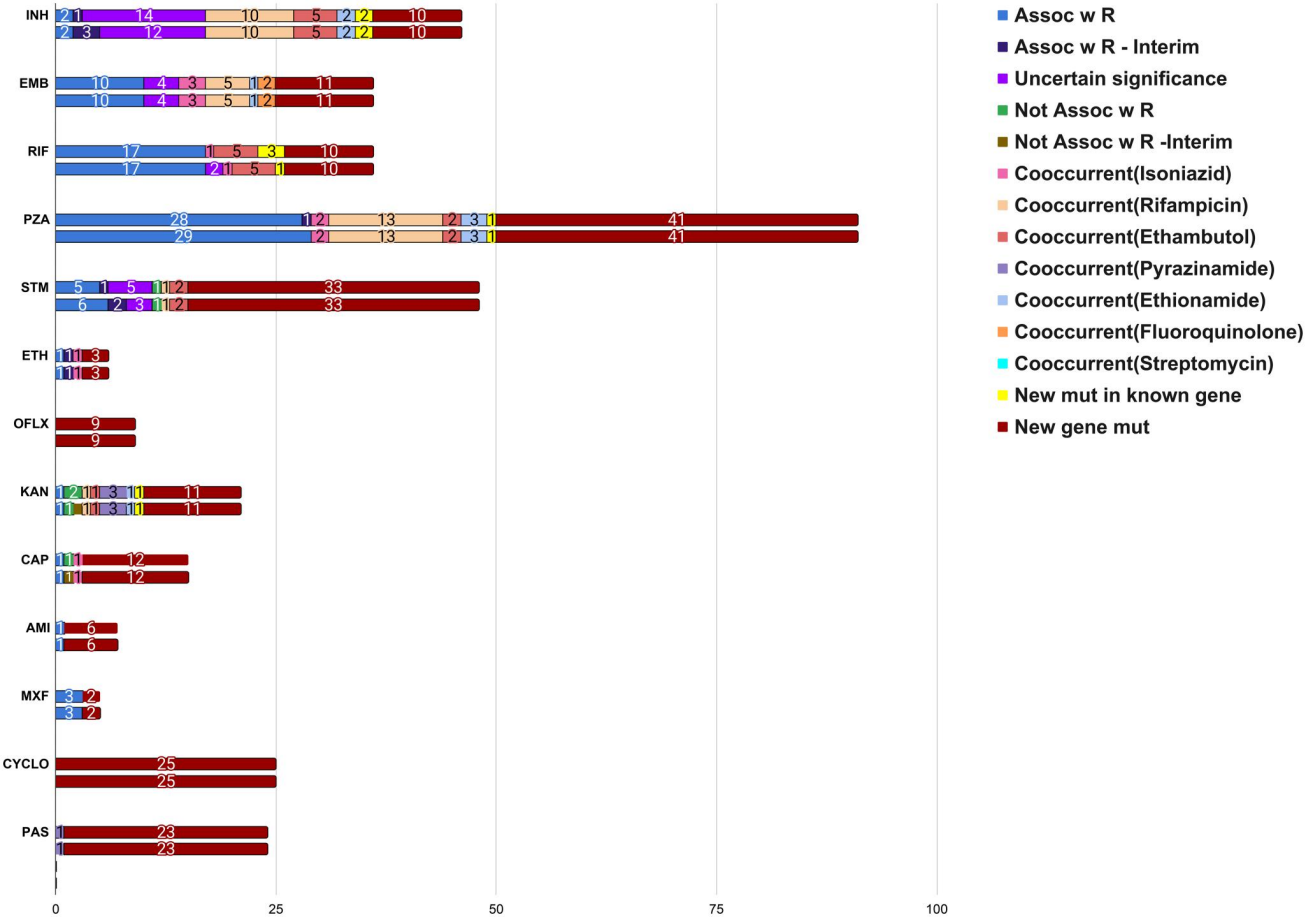
Number of resistance associated mutations predicted by feature extraction method for different drugs. High scoring mutations predicted by feature extraction method were categorized according to the WHO Mutation Catalogue 2021 and 2023 editions. All predicted mutations were compared with mutations reported in WHO list as “Assoc w R,” “Assoc w R -interim,” “Uncertain significance,” “Not assoc w R,” and “Not assoc w R-interim,” Mutations not previously reported in WHO but occurring in known resistance-associated genes were designated as “New mut in known gene,” while mutations in new genes were labeled as “new gene mutation.” Additionally, co-occurring mutations (“Co-occurrent Mutations”) were analyzed. For each drug, the upper bar represented the WHO Mutation Catalogue 2021, and the lower bar represented the WHO Mutation Catalogue 2023.

Since these mutations have similar feature importance or SHAP score as other mutations which overlap with WHO list, these can be considered as bonafide novel DR associated mutations predicted by our unbiased approach. Figure 3 shows a graphical depiction of the overlap of our predicted DR associated mutations with different categories in the WHO mutation list of 2021 and 2023, along with novel DR associated mutations predicted by our ML model in new genes or new mutations in known genes. As can be seen from Fig. 3, we also predict several co-occurring mutations that are associated with DR of other antibiotics. For example, the ML model for INH predicts several “Assoc w R” categories of mutations for RIF, EMB, and ETH. These mutations will not be present in the WHO list as they analyze mutations in a known set of genes for each antibiotic. However, when we analyze the mutations in the entire genome and predict DR associated mutations, we also correctly predict known DR associated mutations for other antibiotics due to the presence of multi-DR strains in our dataset. If we compare the overlap of our predictions for RIF it can be seen that *rpoC*, p.Val483Ala and *rpoC*, p.Val483Gly DR associated mutations predicted by our model moved from new mutations in the known gene category to “Uncertain significance” category as WHO list was refined with the addition of new data. However, our method had successfully predicted them among a limited number of DR associated mutations. These results further demonstrate the success of XAI in the identification of novel DR associated mutations without prior knowledge of any resistance markers. Hence, DR associated mutations in new genes could be bonafide novel resistance markers. Overall, our analysis revealed that a large number of known resistance associated mutations (Assoc w R) could be identified by our automated method for most antibiotics except PZA. The comparison could not be made for PAS, CYCLO, and OFLX as WHO mutation list did not have mutation data for them. We analyzed the percentage of the predicted mutations that overlap with WHO mutation catalogue and the percentage of new mutations for all 13 drugs (Supplementary Fig. S3). As can be seen, a very high percentage of DR associated mutations predicted by our ML models occur in new genes which are not analyzed by WHO or other methods for prediction of DR.

**Table 5.** New mutations detected in those genes that are already known to be associated with resistance by our ML based approach.^a^

Drug	Predicted new mutations on known drug resistance-associated genes
INH	*katG*, p.G124fs; *inhA*, p.P251A
RIF	*rpoB*, p.MD434IY; *rpoC*, p.V483A; *rpoC*, p.V483G
PZA	*pncA*, c.-12A>G
KAN	*eis*, c.-14G>A

a These mutations are not listed in WHO mutation catalogue 2021 (first edition); *rpoC*, p.V483A; *rpoC*, p.V483G is added as “Unknown significance” in WHO mutation catalogue 2023 (second edition). Whereas *inhA*, p.P251A, *rpoB*, p.MD434IY, pncA, c.-12A>G and *eis*, c.-14G>C mutations remains ungraded.

**Figure 4.**
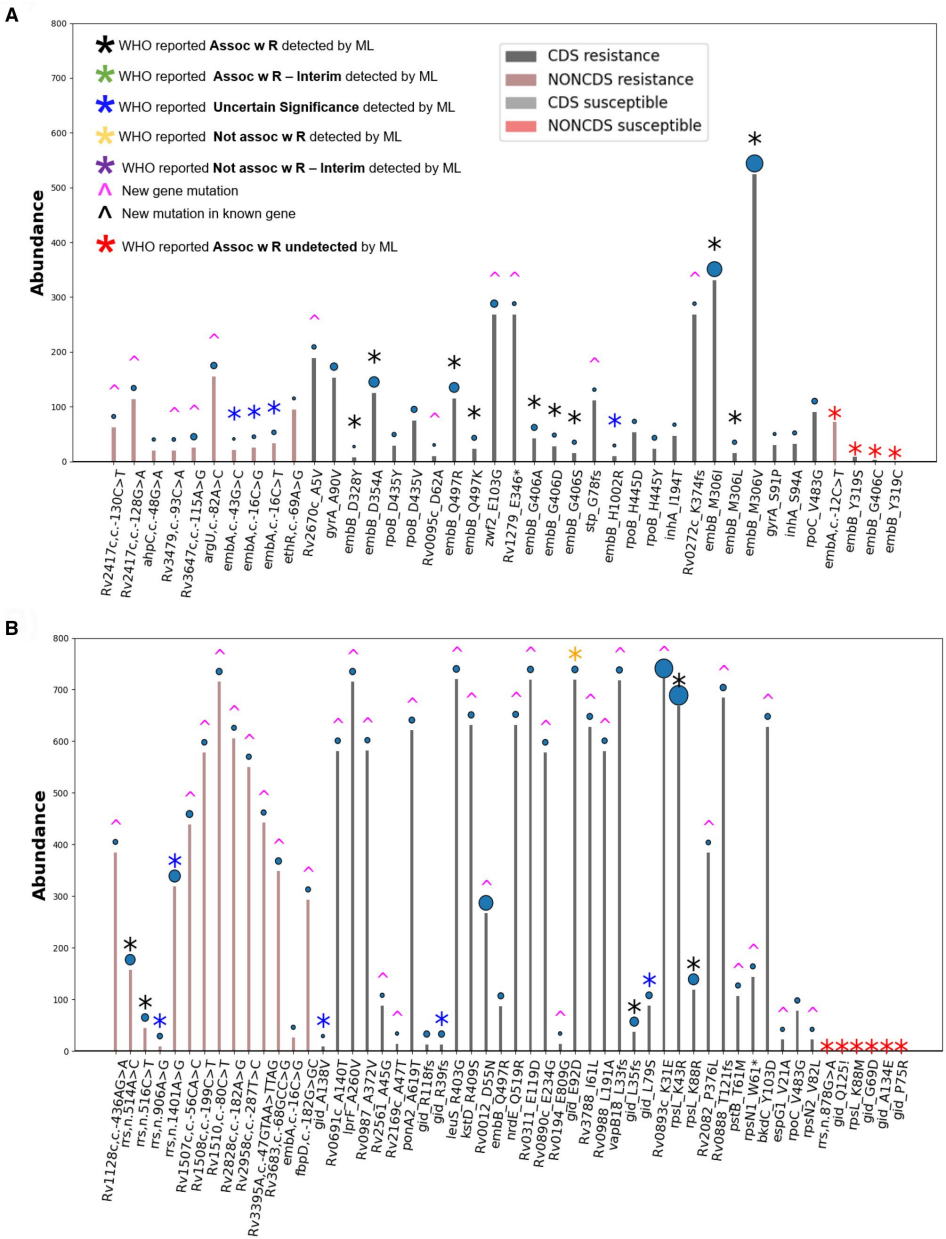
Comparison of the high scoring mutations predicted by feature extraction method with WHO Mutation Catalogue. Panels A and B depict the machine learning predicted scores and relative abundances of each mutation in DR and susceptible *M.tb* populations for EMB and STM, respectively. The predicted mutation profiles for EMB and STM were characterized based on the WHO mutation list. Each mutation is denoted by a distinct color of an asterisk (*), with each color representing a specific WHO gradation while arrow ( ^ ) in magenta and black colours indicated “New gene mutation” and “New mutation in known gene,” respectively, predicted by our ML method. The size of the bubble above each bar corresponds to the associated score, with larger bubbles representing higher scores. Each bar on the chart represents the abundance value within a given training dataset of resistant and susceptible isolates for EMB and STM.

We also checked the abundance and SHAP score of highly significant ML-predicted mutations in both resistant and susceptible populations and compared them with the abundance of WHO mutations which overlapped with ML predicted mutations. Figure 3 shows the results for EMB. As can be seen out of 36 DR associated mutations predicted by our ML model, 10 are mutations in the new genes, 12 are co-occurring mutations, 10 belong to “Assoc w R” category of WHO, and 4 belong to “uncertain significance” category. Out of the 14 “Assoc w R” mutations reported in the WHO dataset, our models can predict 10 “Assoc w R” mutations. Among them, the *embB*, M306V mutation exhibited the highest abundance (31%; *n* = 524) and ML-assigned score. The remaining mutations located in the embB and embA genes, known to be associated with EMB resistance, have been classified by WHO as “Uncertain significance” (Fig. 4A). Our ML models did not detect any new mutations in *embB* and *embA* in the case of EMB. Even though the top two abundant mutations belong to “Assoc w R” category, several low abundance mutations are also ranked as “Assoc w R” in the WHO list and have high SHAP scores as per our XAI scoring. This suggests that abundance in resistant populations is not the only criterion based on which resistance associated mutations can be identified. Since our ML model can take into account the cooccurrence of mutations in addition to their abundance, it has been able to capture a majority of high confidence resistance associated mutations from the WHO list, even if it uses the entire genome without prior knowledge of resistance markers. Figure 4A also shows the abundance of four mutations that belong to “Assoc w R” category as per WHO list, but are not predicted by our ML model (red *). As can be seen, two of them had an abundance count in our dataset below our preset cut-off of 5 genomes, while the third one is a noncoding mutation (*embA*, c.-12C>T) which gets a low SHAP score. This mutation is present in 72 resistance genomes and 6 susceptible genomes. It is noteworthy that in the latest 2023 WHO mutation list, the *embA*, c. -12C>T mutation has been shifted from the “Assoc w R” category to the “Uncertain Significance” category. This re-gradation of the *embA*, c. -12C>T mutation in WHO 2023 list substantiates that our model can accurately predict the significance of mutations that occur in both resistant and susceptible with different abundance. It is also important to note that, WHO 2021 list for EMB analyzes mutations associated with *embA, embB, embC, embR*, and *ubiA*, hence it misses several coding as well as noncoding mutations associated with other genes that have high abundance as well relatively higher SHAP score. These are essentially predicted as mutations in new genes by our ML methods, e.g. *zwf2* (E103G), *Rv3647c* (c.-115A>G), and *argU* (c.-82A>C). It will be interesting to analyze these new genes harboring DR associated mutations for deciphering the mechanism of resistance for EMB.

For STM Fig. 4B shows a similar abundance plot for 48 DR associated mutations predicted by our ML models. Out of a total of 11 “Assoc w R” mutations 5 mutations, *rrs*, n.514A>C (*n* = 10%), *rrs*, n.516C>T (*n* = 3%)*, gid*, p.L35fs (*n* = 2%), *rpsL*, p.K43R (*n* = 45%), and *rpsL*, p. K88R (*n* = 8%) were predicted by our ML model and our model fails to predict six mutations belonging to “Assoc w R” category of WHO. As can be seen, these six mutations had abundance below cut-off in our dataset and many of the 33 new genes predicted by our model have high abundance as well as SHAP score. Since WHO list analyzes only six genes namely gid, rpsL, rrs, Rv1258c, whiB6, and whiB7, mutations in these new genes are not predicted as potential DR associated mutations in WHO 2021 mutation list. Out of these mutations in new genes, two of the top scoring mutations in terms of SHAP score and with high abundance are *Rv0893c*, p. K31E (Ab = 48%), and *Rv0012*-D55N (Ab = 18%). The role of these two genes in conferring STM resistance is not known yet. We have also identified novel gene involvements, as detailed in Supplementary File S6, including *rpsN1* (W61*) and *rpsN2* (V82L), both of which are 30S ribosomal proteins essential for the assembly of 30S ribosomal particles. Since STM targets the 30S ribosomal subunit to inhibit protein synthesis, mutations in these proteins can exert a significant impact on STM drug resistance (Springer *et al.* 2001).

For INH DR associated mutation profile (Supplementary Fig. S4A) our method detected a frameshift mutation *katG*, p.G124fs (*n* = 0.2%) with nucleotide variant c.354_373delCCGCGGCGGCGCCGGGGGCGinsTCGCGGTGGCGCCGGTCAGGGC which is also absent in the WHO 2021 as well as WHO 2023 mutation catalogue. Another new mutation in known gene for INH was detected in *inhA* gene, *inhA*, p.P251A (*n* = 0.6%). Several efflux pump proteins such as *stp, drrA, pstB*, Rv0194, and Rv0987 are predicted by our method as novel gene associations with drug resistance (Supplementary File S6). For CAP we detected a mutation A85V in Rv1830 (Supplementary Fig. S4D), which is known to be associated with drug resilience (Liu *et al.* 2022) by accelerating the recovery of *M.tb* bacteria. Supplementary Figure S4A–K shows similar abundance plots for the other 11 antibiotics. These results demonstrate that even in the absence of any pre-existing knowledge, our models predict a majority of the mutations classified in the WHO mutation catalogue 2021 under “Assoc w R” as mutations with high feature importance score and help to identify several mutations in new genes which correlate strongly with phenotypic DST. Some of the new mutations predicted by our model, such as *rpoC, p.*Val483Ala, *rpoC, p*.Val483Gly, have been recently incorporated in WHO 2023 list. This further supports that our new predictions could be novel resistance markers that are yet to be experimentally discovered.

**Figure 5.**
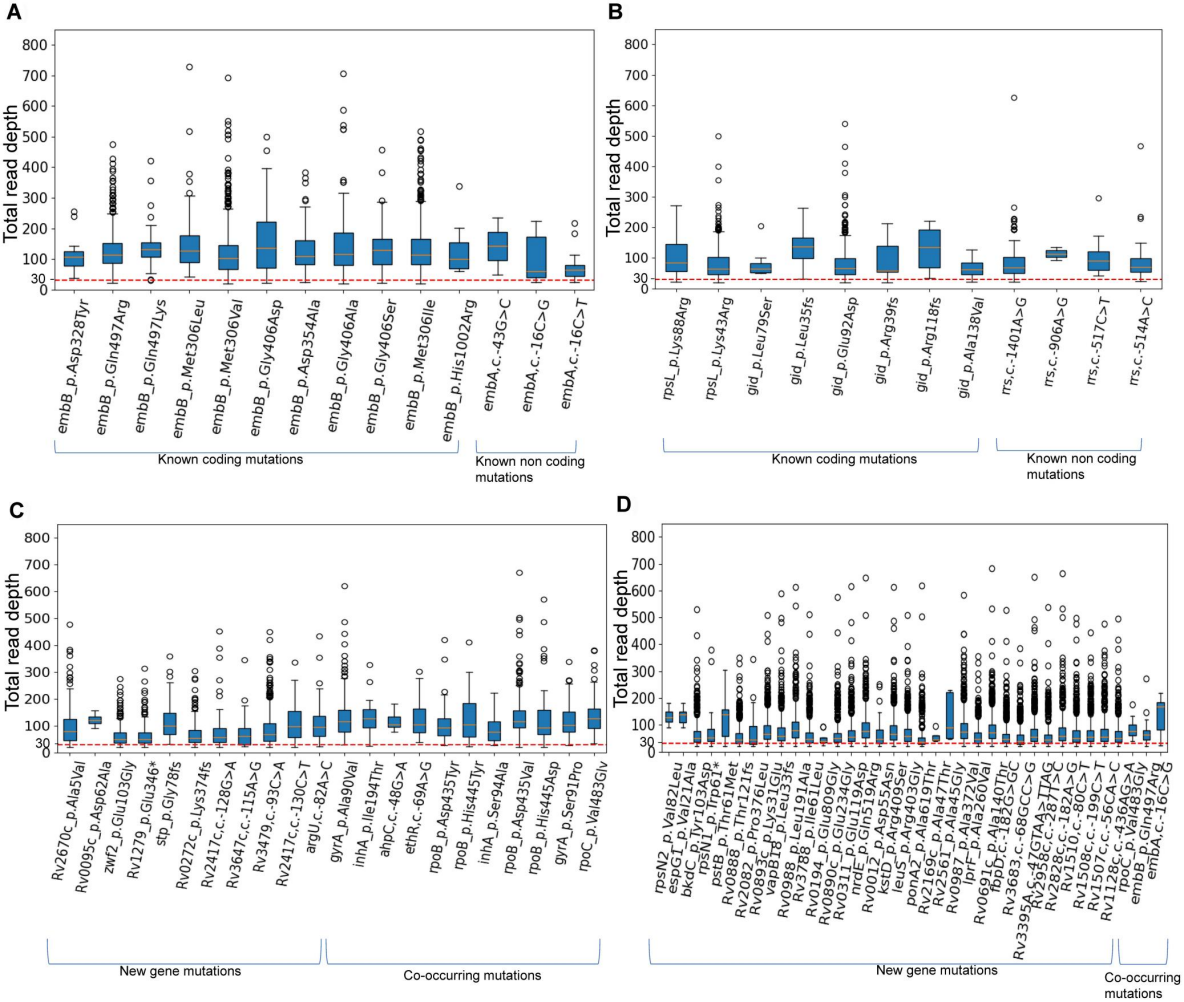
Variant quality analysis in terms of total read depth per base: a box plot showing *y*-axis represents total read depth, while the *x*-axis displays mutations. Panels A and B illustrate known mutations for EMB and STM, respectively, with panels C and D presenting new gene mutations and co-occurring mutations for the same drugs. Each box plot illustrates: the lower whisker indicates the minimum total read depth, while the upper whisker shows the maximum total read depth. The blue box represents the interquartile range, capturing the central 50% of the data distribution. A yellow line within the box denotes the median value, providing a central tendency measure. A red dashed line at a total read depth of 30 serves as a critical benchmarking threshold to distinguish high-confidence mutations. Points extending beyond the lower and upper whiskers are identified as outliers.

In order to demonstrate that the mutations (known and new) predicted by our models are not artifacts from sequencing errors, a comprehensive variant quality analysis was carried out for all the genomes used in the current study (BV-BRC and CRyPTIC) except for 3150 genomes from the BV-BRC dataset, for which accession ID of the FASTQ files were not available in the sample metadata file provided on the BV-BRC. Figure 5 shows the box plot analysis of read counts for all resistance associated mutations for two representative drugs, EMB and STM averaged over all the genomes. Figure 5A and B display read counts for known coding and noncoding mutations, whereas Fig. 5C and D depict read counts for new mutations in known genes and mutations in new genes. Recognizing that a total read depth of 30 or higher provides confident variant calling with a lower error rate, a cut-off threshold was established at 30. As can be seen, the interquartile range (capturing the central 50% of the data distribution) and median values (providing a central tendency measure) for both known and new mutations exceed this cut-off of 30. Similar variant quality verification plots for INH and RIF are shown in Supplementary Fig. S5. The variant quality matrix (averaged over all the genomes) for all the predicted DR associated mutations in all 13 antibiotics is provided in Supplementary File S7. Analysis of the variant quality matrix (Supplementary File S7) revealed that the median read depth for known and new mutations ranged from 31 to 387. Similarly, the predicted resistance-associated mutations had an average alternate allele frequency of 93%–100% and an average base quality score (Q-score or Phred score) ranging from 30 to 39. Variants with alternate allele frequencies above 90% and base quality scores of 20 or higher are considered high-confidence and confirmatory. Hence, our variant quality analysis demonstrates that the predicted DR associated mutations are not sequencing artifacts.

**Figure 6.**
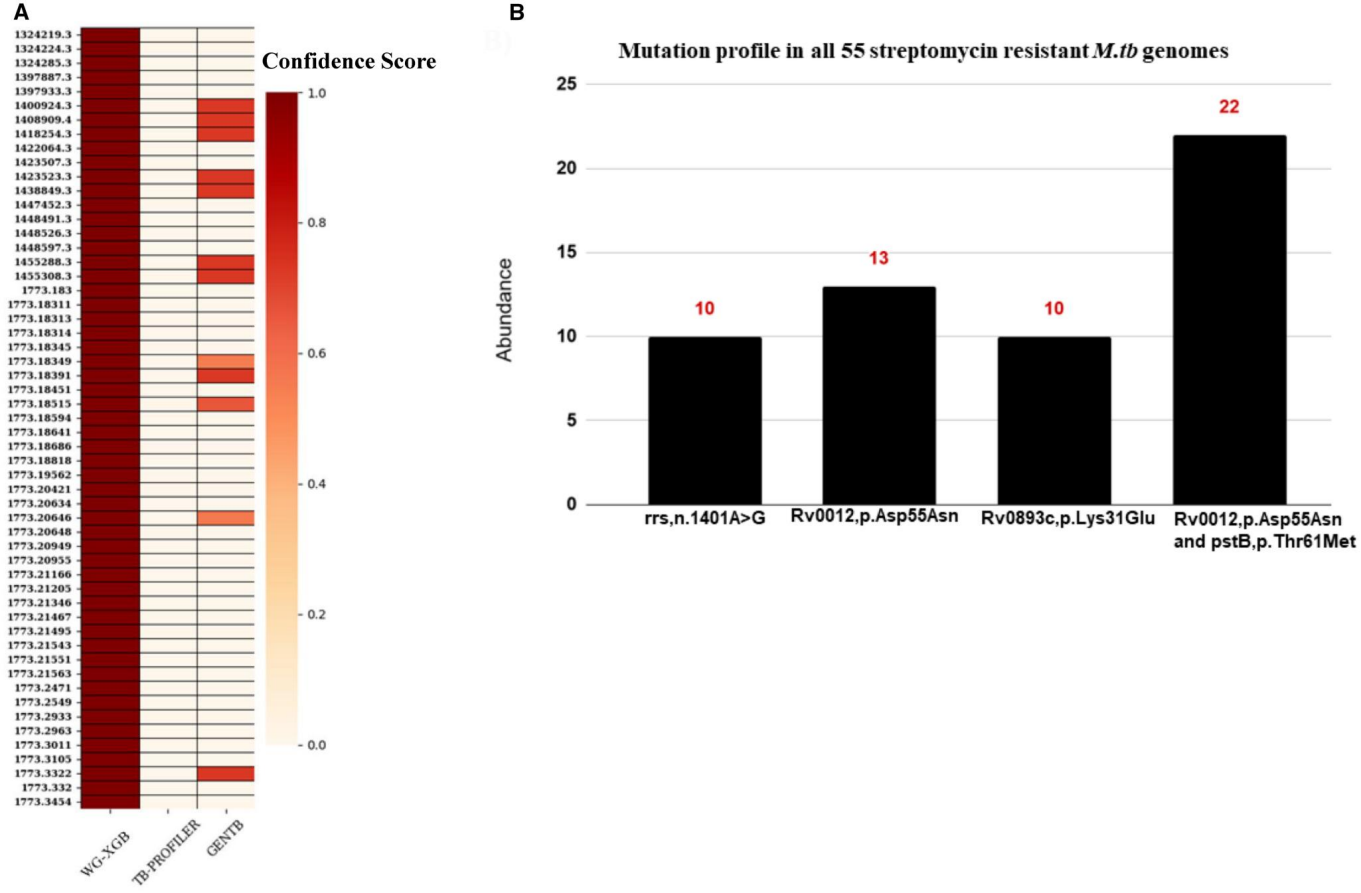
Comparison of the predictions by WG-XGB with TB-profiler and GENTB for samples lacking known mutations associated with STM resistance. The analysis was conducted on 55 *M.tb* variants, utilizing DST phenotype as the gold standard: (A) WG-XGB accurately predicted all 55 *M.tb* variants as STM resistant, whereas TB-profiler failed to make any predictions. GENTB, on the other hand, successfully predicted STM resistance in 12 out of the 55 genomes, albeit with a relatively low confidence score. (B) Prediction of potential mutations that can be associated with STM resistance such as *Rv0893c*, p.Lys31Glu; *Rv0012*, p.Asp55Asn; *pstB*, p.Thr61Met.

The advantage of the DR associated new mutations in known genes and mutations in new genes identified by our method become more apparent when we analyze the false negative predictions by known marker-based methods like TB-Profiler. In our dataset, analysis of a total of 1735 STM resistant genomes (1500 in training and 235 in test dataset), we identified 55 *M.tb* strains conferring STM resistance according to phenotypic DST data, but the corresponding genomes did not harbor any known STM resistance-associated marker mutations, such as mutations in *gid, rpsL* genes. Consequently, TB-profiler failed to predict any of these 55 *M.tb* strains as resistant to STM. Additionally, we tested genomes of these 55 *M.tb* strains using the recently published AI/ML based DR prediction method GENTB. GENTB could predict 12 *M.tb* strains out of 55 as resistant to STM albeit with a very low confidence score. In contrast, our WG-XGB model accurately predicted STM resistance in all 55 strains. Figure 6A shows a comparison of the results of WG-XGB with results from TB-Profiler and GENTB. In fact, WG-ANN method could also predict 49 of these 55 genomes as resistant to STM. We further analyzed the mutation patterns of these 55 variants to determine the presence of novel DR-associated mutations identified by our ML model, which accurately predicted the drug sensitivity of these 55 samples (Fig. 6B). It is interesting to note that novel mutations *Rv0012*, p.D55N and *pstB*, p.T61M co-occurred in 22 genomes. In 13 genomes *Rv0012*, p.D55N was present alone, 10 genomes contained *Rv0893c*, p.K31E alone, and rrs, n.1401A>G was present in the remaining 10 genomes. Since many of these novel mutations occur as a solo in >5 DR samples and they are absent in drug sensitive samples, these could be marked as “Assoc w R” based on WHO criteria. These results indicate that novel resistance associated mutations predicted by our method are bonafide markers of drug resistance. We investigated whether the additional STM resistant genomes lacking known resistance markers exist. The analysis of 1579 other STM resistant genomes in BV-BRC (distinct from our initial dataset of 1735 STM genomes given in Supplementary File S3) using WG-XGB and TB-Profiler revealed 49 additional STM-resistant samples harboring novel mutations identified by our model. TB-Profiler did not predict any of these 49 samples as STM resistant. The IDs for all 104 STM resistant samples (an initial 55 and an additional 49) are provided in Supplementary File S3.

### .3.5 TB-AMRpred webserver

The WG-XGB model was implemented as TB-AMRpred (Tuberculosis Antimicrobial resistance prediction) webserver (Supplementary Fig. S6) for predicting drug resistance profiles using WGS for 13 drugs. As can be seen, for the *M.tb* genome ERR133942 (read accession ID), TB-AMRpred not only predicts correctly drug resistance profiles for 13 antibiotics but also identifies known as well as new DR associated mutations for each of them (Supplementary Fig. S6A). Mutations corresponding to the “Assoc w R” category in the WHO mutation catalogue are highlighted in bold (as shown in Supplementary Fig. S6B). This webserver stands out for its capability for identifying novel resistance-associated mutations.

## 4 Discussion

Our study demonstrates that WG-XGB and WG-ANN exhibit high sensitivity and specificity in predicting resistance, particularly for first-line drugs such as EMB, RIF, PZA, and STM. While WG-XGB models for EMB and RIF yield comparable results, WG-XGB models for STM and PZA outperform the knowledge-based approach (TB-profiler). In the case of second-line drug CAP, the WG-XGB model performs superiorly to TB-profiler, while performance for the remaining second-line drugs is more or less equivalent. WG-ANN models perform optimally with large, complex datasets, as demonstrated by the high accuracy achieved for first-line drugs with comparatively larger training data. However, the performance of WG-ANN models for second-line drugs degrades due to smaller dataset sizes. ML algorithms like XGBoost show more resilience in such low-data environments. Enhancing WG-ANN model performance for second-line drugs requires incorporating more diverse and representative data points, but this was limited in the current study by the lack of sufficient resistant samples.

The sensitivity and specificity of drug resistance prediction for CYCLO and PAS are compromised due to limited phenotypic data availability, yet they still outperform the TB-profiler by 27% and 34% better sensitivity, respectively, at almost similar specificity. On the CRyPTIC dataset, our WG-XGB models perform comparably to TB-profiler. A notable advantage of using a combination of XGBoost internal feature extraction and SHAP based approach is that this method can detect hidden loci in genomic regions associated with drug resistance. Such loci are promising for further investigation as drug targets. Even though new resistance associated mutations predicted by our method in hidden loci provide valuable insights, it will be necessary to carry out experimental validation to establish their functional significance. Since the models were constructed using WGS information, noncoding mutations that influence resistance in *M.tb* are also predicted by our approach. This is a significant advantage over knowledge-based approaches, which often overlook these potential regions critical for resistance. However, our method also excludes PE/PPE/PGRS regions, because they are highly repetitive and contain a high rate of mutations because of intrinsically disordered regions. Given the complexity of interpreting regulatory mechanisms of the noncoding variants and potential limitations arising from the exclusion of PE/PPE/PGRS regions, the newly identified loci cannot be conclusively linked to drug resistance mechanisms. Therefore, it is essential to emphasize that these identified mutations represent promising candidates requiring further experimental investigation, such as functional assays, gene expression studies, and targeted genome editing, to conclusively demonstrate their role in drug resistance mechanisms. Similarly, though our models for a given drug sometimes predict resistance associated mutations for other drugs as co-occurring mutations, experimental studies will be necessary to verify if they really indicate biological or genetic interactions between resistance mechanisms.

In a recent study, Kavvas *et al.* used a reference agnostic ML approach on a dataset of 1595 *M.tb* strains and demonstrated that they could not only identify 33 known DR associated genes, but also predicted 24 new DR associated genes such as Rv3848, Rv3129, *proC*, and *kdpC* (Kavvas *et al.* 2018). These results support the observations from the current study which identifies a large number of new drug resistance-associated genes for 13 drugs based on analysis on a much larger dataset consisting of >13 000 genomes.

In addition, our feature extraction method successfully identified mutations associated with resistance, including those already documented in the WHO Mutation Catalogue 2021. Furthermore, the methods detect several new mutations in known drug resistance-associated genomic regions, as well as in some novel genes. While the interpretation of new mutations in known drug resistance associated genomic regions is straightforward, the identification of novel gene mutations requires further experimental validation. Interestingly many of the new DR associated genes predicted by our ML model map to efflux pumps, including *stp*, p.G78fs for EMB and RIF, *drrA*, p.R262G for ETH, *Rv0194*, p.E809G for STM, *mmr*, c.-29C>CATGTACAA for PZA, and *pstB*, p.T61M for STM. It has been proposed that mutations associated with efflux pumps in *M.tb* often lead to pump overexpression, as seen in clinical isolates resistant to Bedaquiline with Rv0678 mutation (Ismail *et al.* 2019, Laws *et al.* 2022).

We have identified 55 *M.tb* variants demonstrating STM resistance according to DST data, which TB-profiler failed to predict. GENTB also predicted only 12 out of these 55 variants as STM resistant, but with a very low confidence score, given that GENTB generates a probability score for antibiotic resistance rather than providing a binary prediction. Upon examining the mutation profile generated by our model, we observed the absence of known STM-resistant markers. According to our model, potential novel genes, such as *Rv0893c*, *Rv0012*, and *pstB*, may be responsible for conferring resistance in these 55 variants. These results demonstrate the advantage of our WG-XGB model, as it can detect novel resistant variants, a challenging task for knowledge-based methods based on known resistance markers.

Similarly, WG-XGB performed better than TB-profiler with 8% and 11% higher sensitivity for PZA and CAP, respectively, while maintaining the same specificity. These results suggest that the WG-XGB model has the potential to improve the accuracy of tuberculosis drug resistance prediction, which could have significant implications for TB diagnosis and treatment. Even though the WG-XGB model is found to have a lower sensitivity of 6% compared to the TB-Profiler in detecting KAN resistance, it has an MCC value of 0.87 which matches with TB-Profiler. Since the development of ML models for the prediction of DR requires both genomic sequence and phenotypic DST data, currently it is not possible to develop meaningful prediction models for new drugs like Bedaquiline, Linezolid, etc.

Despite the advantages of our whole genome based method over other knowledge-based methods for the prediction of drug resistance in *M.tb*, our method has a few methodological limitations. Our current pipeline relies heavily on reference genome based variant calling. Traditional variant calling approaches, including tools like SNIPPY, predominantly focus on mapped genomic reads and potentially overlook critical genetic information coming from unmapped genomic regions. These unmapped genomic regions arise from highly variable sequences, structural variants, and novel insertions, and may contain significant biological insights that our current method might inadvertently exclude. Split mapping techniques can partially mitigate this challenge by fragmenting and remapping reads. Using a single reference genome is another problematic limitation of our method. Variations in reference genome selection can substantially influence prediction outcomes, introducing potential systematic biases. Future work could address these methodological constraints to enhance the efficiency and precision of drug resistance prediction in *M.tb*.

## 5 Conclusions

We have developed a novel machine learning approach that can predict drug resistance of *M.tb* for 13 anti-tubercular drugs from WGS without using information on known resistance associated mutations for different drugs. Extensive benchmarking studies indicate that our ML model implemented as TB-AMRpred can predict genotypic drug resistance with high accuracy. In addition, to the best of our knowledge, it is the only method that can predict resistance associated mutations using a completely automated approach. The unique feature of our model is the use of an XAI approach involving XGBoost feature scoring function, and SHAP^0.41.0^ (SHapley Additive exPlanations) framework to analyze mutations in the entire genome for identifying drug resistance associated mutations. Even though our model does not use information on known resistance markers for training, it can automatically identify known as well as new mutations associated with DR because of the power of an XAI framework. A comparison of our results with WHO mutation catalogue reveals that our XAI approach has correctly identified a significant number of known mutations associated with resistance to different drugs. In addition, it has also predicted several new mutations in known genes as well as mutations in new genes as putative DR associated mutations. Many of the new resistance associated genes predicted by our ML model belong to the family of efflux pumps, ribosomal proteins, and methyltransferases, which are typical protein families associated with drug resistance. We strongly believe that our approach offers key insights into previously unknown aspects of drug resistance, enhancing the overall understanding of this critical issue.

## Supplementary Material

vbaf050_Supplementary_Data

## Data Availability

TB-AMRpred webserver can be accessed freely at http://www.nii.ac.in/TB-AMRpred.html, no login is required. Source code for the standalone version can also be download freely from the “Download” link of the TB-AMRpred webserver or from GitHub at https://github.com/Ankitapal1995/TB-AMRpred. Supplementary Files S3–S8 are available at https://github.com/Ankitapal1995/TB-AMRpred/tree/main/Additional_excel_files
