# Supplementary material for: Machine learning-based approach for identification of new resistance associated mutations from whole genome sequences of Mycobacterium tuberculosis
Source: Bioinform Adv. 2025 Mar 11;5(1):vbaf050. doi: 10.1093/bioadv/vbaf050 (PMC11930343; doi:10.1093/bioadv/vbaf050)
Supplement: vbaf050_Supplementary_Data [file vbaf050_supplementary_data.zip › Supplementary File 1.docx]

**Supplementary Methods**

**Compilation of WGS of *M.tb* strains with known antibiotic resistance phenotype data**

For training and test datasets, all the whole genome *fasta* sequences and respective lab typing method-based drug susceptibility test results (phenotypic data) were gathered from the BV-BRC (previously known as PATRIC database[1]) excluding all the sequences taken from CRyPTIC. A total of 13947 whole genome *fasta* sequences were used to train 13 different drug-resistant models including first-line and second-line drugs (first-line drugs: Isoniazid (INH), Ethambutol (EMB), Rifampicin (RIF), Pyrazinamide (PZA), Streptomycin (STM), and second-line drugs: Ethionamide (ETH), Ofloxacin (OFLX), Kanamycin (KAN), and Capreomycin(CAP), Amikacin (AMI), Moxifloxacin (MXF), Cycloserine (CYCLO), Para-aminosalicylic acid (PAS) (**Table 1**), whereas 12602 sequences were taken separately to test (**Table 2**) those models. It is important to note that while the training and test datasets are reported separately, there is an overlap between them due to the presence of multidrug-resistant strains. For training, we kept the dataset balanced by taking an equal number of drug-resistant and antibiotic-susceptible isolates to avoid model biasedness. For susceptible isolates, we have selected those labelled as susceptible for all drugs.

**Generation of feature vectors for machine learning**

As we aimed to build a data-driven decision-making tool, we did not use any domain or genomic region-specific knowledge of antibiotic resistance-conferring properties in *M.tb* to feed the model. Instead of that, we transformed whole genome sequence information in terms of mutations using variant calling. A well-known variant calling tool named SNIPPY^4.6.0^[2] has been used for aligning each  *M.tb* whole genome *fasta* sequence with a reference sequence  *M.tb* H37Rv (Genome assembly ID: GCA_000195955.2, biosample ID: SAMEA3138326) and all of its SNPs and INDELs both in coding and non-coding regions were gathered. Regarding the resistance dataset, we created a master file containing the unique mutations and their abundance. We also created a similar file for the susceptible dataset. We then proceeded according to the steps outlined in **Supplementary** **Figure S1**. For data processing we applied filters to all mutation lists of drug-resistant *M.tb* strains used in the training dataset followed by discarding i) all the synonymous or silent mutations which are defined as low-impact variants annotated by SNIPPY ii) *PE, PPE, PE-PGRS* region mutations( SNPs and INDELs) as these regions are highly repetitive so it contains high rates of mutations due to the presence of intrinsically disordered regions. Also these regions are very difficult to sequence using short-read sequencing techniques. However, these proteins occupied almost 10% of *M.tb* proteomes, which will create a bias in the model and affect the analysis[3]. iii) mutations occurring in 0.2% of drug-resistant as well as drug-susceptible *M.tb* genomes according to the respective sample size. The remaining mutations of all the drug-resistant *M.tb* strains were finally taken as features (as shown in **Supplementary Table S1**) to create the presence-absence binary matrix for each  *M.tb* genome using an in-house python script. We assigned ‘1’ if a mutation is present in an *M.tb* genome otherwise we assigned ‘0’. This matrix will be loaded to build the model using *scikit-learn^1.1.1^*[4] framework.

**Building the machine learning models**

Whole genome extreme gradient boost(WG-XGB) and Whole genome artificial neural network (WG-ANN) models were built for every 13 drugs. For WG-ANN models were implemented with *Keras*(v3.0.1)[5] upon *Tensorflow*(v2.10)[6], comprising one input layer which involves the same number of features taken in model-building as input dimension, two hidden layers having 8 units of ReLU activation function and 1 unit of ‘Sigmoid’ activation function respectively. All the WG-ANN models were trained for 10 epochs using stochastic gradient descent and ‘Adam’ optimizer. The WG-XGB models were trained with a “gpu_hist” tree method, random_state of 1, learning_rate of 0.1 and other parameters with the default values of the current release of XGBoost (XGBoost^1.7.1)^)[7]. To build an unbiased model we used a balanced dataset of an equal number of drug-resistance and susceptible  *M.tb* genomes for each drug. 2-fold, 5-fold, 10-fold, and leave-one-out cross-validation (LOOCV) were used to evaluate the performance of the training dataset. The hold out dataset (12,602 genomes) used for testing the performance of the ML classifiers was imbalanced as phenotypic drug susceptible samples in our dataset were much higher than the phenotypic drug resistant samples. Both the WG-XGB and WG-ANN models developed in the current study as well as the TB-Profiler were benchmarked on the same dataset using taking their DST labels as a gold standard and performances of the models were evaluated using standard statistical measures namely sensitivity (Sn) or recall and specificity (Sp).

$$Sn or Recall=\frac{TP}{TP+FN}$$

$$Sp=\frac{TN}{TN+FP}$$

Where *TP, FP, TN, FN* are number of ‘true positive’, ‘false positive’, ‘true negative’ and ‘false negative’ predictions.

Overall model performance was analyzed using the Matthews Correlation Coefficient (MCC), which is a measure of the quality of binary classifications. An improved binary classification model's performance is typically demonstrated by a higher MCC score and it is calculated using the following formula.

$$MCC= \left( TP\times TN \right)-\left( FP\times FN \right)$$

$\sqrt{\left( TP+FP \right)\left( TP+FN \right)\left( TN+FP \right)\left( TN+FN \right)}$

The MCC values have a range from -1 to +1, where +1 represents a perfect prediction, 0 represents a random prediction, and -1 represents a complete disagreement between the predicted outcome and the observation.

Similarly, precision or Positive Predictive Value (PPV), Negative Predictive Value (NPV), F1 score, and accuracy were considered to evaluate the overall model performance.

Precision quantifies the accuracy of positive predictions by calculating the proportion of correctly identified positive instances among all positive predictions.

$$Precision or PPV=\frac{TP}{TP+FP}$$

Negative Predictive Value (NPV) is a statistical metric that represents the proportion of actual negative cases correctly identified by a classification model among all instances predicted as negative.

$$NPV=\frac{TN}{TN+FN}$$

F1 score provides a balanced metric that combines precision and recall using the harmonic mean, providing a single comprehensive performance indicator. The closer the score is to 1, the better the model's predictive capabilities.

$$F1 score=\frac{\left( 2*Precision*Recall \right)}{Precision+Recall}$$

Accuracy measures the overall correctness of predictions of a model. It represents the proportion of correct predictions made by a model out of the total number of predictions.

$$Accuracy=\frac{TP+TN}{TP+TN+FP+FN}$$

**Benchmarking of the ML models on BV-BRC dataset**

To assess all WG-XGB and WG-ANN training models' performance, held test datasets totaling 12602 isolates (as shown in **Table 2**), were utilized. The evaluation was based on these parameters: sensitivity(recall), specificity, and MCC, precision (PPV), NPV, F1 score and accuracy with actual DST data serving as the ground truth. The TB-Profiler “fasta_profile” module was also run on the same test dataset to compare the models' drug-resistance prediction performance in terms of sensitivity(recall), specificity, MCC, precision (PPV), NPV, F1 score, and accuracy.

**Benchmarking on CRyPTIC dataset**

To assess the real-world applicability of our WG-XGB and WG-ANN models, we obtained the "CRyPTIC_reuse_table_20211019.csv"[8] dataset from ftp.ebi.ac.uk/pub/databases/cryptic/release_june2022/. This dataset comprises a total of 12,287 Run accession IDs and laboratory-based drug susceptibility phenotypic data for 13 drugs associated with each isolate (Amikacin, Isoniazid, Rifampicin, Ethambutol, Kanamycin, Moxifloxacin, Ethionamide, Bedaquiline, Clofazinimine, Delamide, Linezolid, Levofloxacin and Rifabutin). Taking this data as the ground truth, we conducted predictions using only common drug models such as INH, RIF, EMB, KAN, AMI, ETH, and MXF to predict susceptibility. The TB-Profiler "fasta_profile" module was executed on the identical test dataset to assess and compare the drug-resistance prediction performance of the models in relation to sensitivity(recall), specificity, MCC, precision (PPV), NPV, F1 score, and accuracy.

**Comparison of the imbalanced and balanced test datasets from both the BV-BRC held-out test dataset and CRyPTIC dataset.**

The effect of an imbalanced test dataset on model accuracy was evaluated using down sampling technique by employing ‘RandomUnderSampler’ module of scikit-learn, which was employed to create a balanced test dataset. In this approach, the total number of minority positive class instances (drug-resistant samples) was maintained, while the majority negative class (drug-susceptible samples) was balanced by randomly selecting an equivalent number of instances. This method ensures a proportional representation of both classes, mitigating potential bias introduced by a class imbalance in the predictive modeling process.

**Feature importance analysis for identification of DR associated mutations**

As we trained our model without feeding any prior knowledge of drug resistance hence, analyzing feature impact on model decision-making became necessary. Since XGBoost algorithm is based on random forest, we can get the information about each feature contribution in model prediction. Using XGBoost^1.7.1^ inbuilt function “get_booster().get_score() taking “gain” as importance_type, the feature important score was computed. The features having a “0” score were discarded and the rest were taken for downstream analysis.

In the case of WG-ANN model, for extracting important feature information, we implemented SHAP^0.41.0^(SHapley Additive exPlanations) framework which is a Python library, reconcilable with almost all types of machine learning model topologies for explaining the model outcomes. With “DeepExplainer()” function of SHAP, all the WG-ANN models were elucidated globally (global explainability) in terms of feature ranking based on their contribution to the model prediction. All the important features were captured followed by removing low-ranked features using a cut-off 0.01 from the analysis. Given the inherent differences in scoring scales between these methods, we normalized the scores using an in-house Python script with scikit-learn preprocessing.normalize function, ensuring comparable and standardized feature importance assessments across both models. After which we combined the normalized scores from both XGBoost's internal feature module and SHAP to create a unified list of highly relevant resistant mutations (**Supplementary Figure S2**).

**Comparison of predicted mutations with WHO mutation catalogue.**

We compared our predicted highly relevant resistant mutations to “Assoc w R” category mutations for each drug listed in WHO catalogue first edition published in 2021[9]. For each predicted mutation we also have taken into account their abundance in resistant and susceptible *M.tb* populations of each drug. Our ML-predicted mutations were compared based on the five categories listed in the WHO mutation catalogue such as 1) “Assoc w R” 2) “Assoc w R-interim” 3) “Uncertain significance” 4) “Not assoc w R” 5) “Not assoc w R-interim”. Mutations were classified as "Assoc w R” when they were identified as solo mutations using WHO-endorsed category 1 and 2 methods across at least five DR *M.tb* isolates with a positive predictive value (PPV) with a 95% confidence interval (CI) lower bound of at least 0.25, an odds ratio (OR) of 1 or higher, and a statistically significant p-value after false discovery rate (FDR) correction. Mutations will be considered as “Assoc w R – Interim” if they occur in fewer than 5 DR *M.tb* isolates as solo mutations with PPV ≥ 50%. Mutations were classified as "Not assoc w R" if PPV with 95% CI upper bound for phenotypic resistance was less than 10%. Mutations that did not meet the criteria for definitive resistance association or clear non-association were designated as having "Uncertain significance". Unlike other drugs, in PZA resistance, numerous individually infrequent mutations are dispersed across the *pncA* gene[10]. Therefore grading criteria are a bit relaxed for PZA. Mutations present as a solo in *pncA* in at least two resistant DR *M.tb* isolates and with at least 50% PPV were classified as “Assoc w R – Interim”. If any new mutations are not reported yet in WHO mutation category but are found to occur in known resistance associated regions we named those as “New mut in known gene” whereas mutations appearing in entirely new genes with unreported involvement as 'New mutations'. However, Given the substantial overlap in phenotypes among multi-drug resistant *M.tb* genomes, ML-based approaches often identify mutations associated with resistance in other drugs. These mutations or genetic alterations are termed Co-occurrent mutations.
**Validation of each predicted mutation using a variant quality matrix:**

To evaluate whether predicted mutations were artifacts of sequencing errors, annotated VCF files were generated using SNIPPY from the corresponding FASTQ files of all the samples taken from BV-BRC, and CRyPTIC datasets. From a total of 14,735 unique dataset samples including training and testing, we successfully performed variant quality assessment and genome assembly on 11585 genomes, utilizing sample metadata information retrieved from the BV-BRC. Accession IDs of the FASTQ files were not available in the sample metadata file for the remaining 3150 samples. In contrast, for the CRyPTIC dataset, we obtained and conducted a variant quality assessment on all 12,287 sample FASTQ files, ensuring comprehensive genomic analysis across both datasets. Within the SNIPPY variant calling pipeline, Freebayes is utilized for VCF file generation, while snpEff performs variant annotation, facilitating comprehensive genomic variant identification. Predicted mutations from drug-resistant *M.tb* samples were systematically collected and statistically analyzed, focusing on key variant quality metrics. These metrics included calculating the median, first, and third quartile values of total read depth per base, average alternate allele frequency with standard deviation, and average base quality score with standard deviation. This approach allowed us to validate predicted genetic variations, distinguishing genuine mutations from sequencing artifacts.

**Implementation as a webserver**

We have implemented our WG-XGB based method as a webserver named ‘TB-AMRpred’ on a LINUX machine using HTML, CSS, Javascript, python flask framework, and Apache webserver. TB-AMRpred takes the whole genome sequence in FASTA format or WGS reads in FASTQ format and uses SNIPPY in the backend to identify mutations for the prediction of DR. It predicts resistance profile for 13 antibiotics and the DR associated mutations for each of them.

**SUPPLEMENTARY INFORMATION**

**Supplementary File 1:** Supplementary Methods

**Supplementary File 2**: Description of all the table and figures are in below: **Table S1**: Total number of mutations are taken for model building. **Table S2**: 2-fold, 5-fold, 10-fold and leave-one-out cross validation results of the training dataset. **Table S3**: Comparison of model predictive performance on test dataset taken from BV-BRC. **Table S4:** Comparison of model predictive performance between imbalanced and balanced BV-BRC test dataset. **Table S5**: Comparison of model predictive performance on test dataset taken fromCRyPTIC. **Table S6:** Comparison of model predictive performance between imbalanced and balanced CRyPTIC dataset. **Table S7:** Total number of important mutations extracted by XGBoost and SHAP method. **Table S8**: Mutation wise list of WHO 2021 and WHO 2023 and XAI predicted mutations. **Table S9**: WHO-endorsed list of genes associated with drug resistance in *M.tb.* **Figure S1**: Method of feature generation. **Figure S2:** Method of extracting important mutations. **Figure S3**: Comparison of predicted mutation with WHO mutation catalogue in terms of percentage. **Figure S4**: The mutation profiles of all 13 drugs, along with their respective abundance in both resistant and susceptible *M.tb* populations, are presented alongside the ML predicted scores. **Figure S5:** Mutation quality analysis of INH and RIF.
**Figure S6:** Overview of TB-AMRpred webserver.

**Supplementary File 3**: Accessions and phenotypic drug resistance data used for training and test dataset of BV-BRC and accession IDs of 55 and 104 STM resistant *M.tb* isolates with novel mutation patterns.

**Supplementary File 4**: CRyPTIC dataset accession IDs and its laboratory based drug susceptible data.

**Supplementary File 5**: Drug resistance prediction and associated mutation using WG-XGB on CRyPTIC dataset.

**Supplementary File 6:** Novel genes predicted by ML method with their score and functions.

**Supplementary File 7:** Variant quality matrix of all predicted mutations including known and new.

**Supplementary File 8:** Sensitivity and Specificity comparison of WG-XGB, WG-ANN, TB-PROFILER, WHO mutations, GENTB-RF, GENTB-WDNN,MD-CNN, SD-CNN

**Reference:**

1. Antonopoulos, D.A., et al., *PATRIC as a unique resource for studying antimicrobial resistance.* Brief Bioinform, 2019. **20**(4): p. 1094-1102.

2. Seemann, T. *SNIPPY; Rapid haploid variant calling and core genome alignment*. Available from: <https://github.com/tseemann/snippy>.

3. Gomez-Gonzalez, P.J., et al., *Functional genetic variation in pe/ppe genes contributes to diversity in Mycobacterium tuberculosis lineages and potential interactions with the human host.* Front Microbiol, 2023. **14**: p. 1244319.

4. Pedregosa F, V.G., Gramfort A, Michel V, Thirion B, Grisel O, et al., *Scikit-learn: Machine Learning in Python.* Journal of Machine Learning Research, 2011.

5. Chollet F, e.a. *Keras*. 2015; Available from: <https://keras.io/>.

6. Abadi M, A.A., Barham P, Brevdo E, Chen Z, Citro C, et al. *TensorFlow: Large-Scale Machine Learning on Heterogeneous Systems*. 2015; Available from: <https://www.tensorflow.org/>.

7. Friedman, J.H., *Greedy function approximation: A gradient boosting machine.* Ann. Statist. 29 (5), October 2001.

8. Consortium, C.R., et al., *Prediction of Susceptibility to First-Line Tuberculosis Drugs by DNA Sequencing.* N Engl J Med, 2018. **379**(15): p. 1403-1415.

9. Walker, T.M., et al., *The 2021 WHO catalogue of Mycobacterium tuberculosis complex mutations associated with drug resistance: A genotypic analysis.* Lancet Microbe, 2022. **3**(4): p. e265-e273.

10. Koser, C.U., D.M. Cirillo, and P. Miotto, *How To Optimally Combine Genotypic and Phenotypic Drug Susceptibility Testing Methods for Pyrazinamide.* Antimicrob Agents Chemother, 2020. **64**(9).
