# Supplementary material for: Machine learning-based approach for identification of new resistance associated mutations from whole genome sequences of Mycobacterium tuberculosis
Source: Bioinform Adv. 2025 Mar 11;5(1):vbaf050. doi: 10.1093/bioadv/vbaf050 (PMC11930343; doi:10.1093/bioadv/vbaf050)

**Supplementary Table S1**: Total number of mutations are taken to build a final presence absence matrix.

| **Drugs** | **Total number of features (mutations)** |
| --- | --- |
| INH | 5511 |
| EMB | 3185 |
| RIF | 5030 |
| PZA | 27585 |
| STM | 2832 |
| ETH | 21619 |
| OFLX | 14333 |
| KAN | 3552 |
| CAP | 2289 |
| AMI | 13437 |
| MXF | 18122 |
| CYCLO | 3787 |
| PAS | 5287 |

**Supplementary Table S2: Results of 2-fold, 5-fold, 10-fold, and leave-one-out (LOO) cross-validation for the training dataset of five first-line and eight second-line drugs.** Sn=sensitivity; Sp=specificity. Bold values in **blue** indicate the highest values of Sn and Sp, while bold values in **green** indicate the lowest values of Sn and Sp.

| Type of drugs | **Drugs** | **Training dataset** | | **2-fold** | | **5-fold** | | **10-fold** | | **LOO** | |
| --- | --- | --- | --- | --- | --- | --- | --- | --- | --- | --- | --- |
|  |  | **Resistance** | **Susceptible** | **Sn** | **Sp** | **Sn** | **Sp** | **Sn** | **Sp** | **Sn** | **Sp** |
| First-line | INH | 3512 | 3512 | **0.78** | 0.99 | 0.79 | 0.99 | 0.80 | 0.99 | **0.83** | **0.99** |
|  | EMB | 1700 | 1700 | **0.85** | 1 | 0.88 | 1 | 0.89 | 1 | **0.91** | **1** |
|  | RIF | 2750 | 2750 | **0.89** | 1 | 0.91 | 1 | 0.91 | 1 | **0.93** | **1** |
|  | PZA | 1500 | 1500 | **0.94** | 0.99 | 0.94 | 0.99 | 0.95 | 0.99 | **0.95** | **0.99** |
|  | STM | 1500 | 1500 | **0.84** | 1 | 0.85 | 1 | 0.85 | 1 | **0.89** | **1** |
| Second-line | ETH | 1076 | 1076 | **0.70** | 0.79 | 0.72 | 0.79 | 0.72 | 0.79 | **0.78** | **0.79** |
|  | OFLX | 959 | 959 | **0.85** | **0.94** | 0.86 | 0.94 | 0.86 | 0.95 | **0.87** | **0.96** |
|  | KAN | 500 | 500 | **0.47** | 1 | 0.70 | 1 | 0.76 | 1 | **0.87** | **1** |
|  | CAP | 400 | 400 | **0.69** | 1 | 0.81 | 1 | 0.82 | 1 | **0.85** | **1** |
|  | AMI | 350 | 350 | **0.83** | 1 | 0.83 | 1 | 0.83 | 1 | **0.84** | **1** |
|  | MXF | 242 | 242 | **0.84** | **0.90** | **0.85** | 0.92 | **0.85** | 0.93 | **0.85** | **0.95** |
|  | CYCLO | 100 | 100 | **0.35** | 1 | 0.46 | 1 | 0.56 | 1 | **0.64** | **1** |
|  | PAS | 90 | 90 | **0.12** | 1 | 0.51 | 1 | 0.56 | 1 | **0.62** | **1** |

**Supplementary Table S3: Model evaluation on the BV-BRC hold out test dataset and comparison with TB-Profiler:** BV-BRC test dataset model evaluation of both WG-XGB and WG-ANN and comparison with TB-profiler based on precision/PPV, NPV and accuracy. The bolded numbers indicates our model performing better than TB-profiler. R= Resistant, S=Susceptible

| Drugs | | INH | EMB | RIF | PZA | STM | ETH | OFLX | KAN | CAP | AMI | MXF | CYCLO | PAS |
| --- | --- | --- | --- | --- | --- | --- | --- | --- | --- | --- | --- | --- | --- | --- |
| R | | 288 | 200 | 278 | 230 | 235 | 63 | 100 | 38 | 38 | 62 | 35 | 26 | 11 |
| S | | 3186 | 3642 | 5178 | 1796 | 916 | 1036 | 890 | 740 | 587 | 596 | 831 | 362 | 283 |
| WG-XGB | Precision/PPV | **79%** | **93%** | **64%** | 95% | **99%** | 16% | 67% | 60% | **99%** | **90%** | 44% | 29% | 46% |
| WG-ANN |  | 57% | 63% | 40% | 66% | 96% | 8% | 11% | 22% | 74% | 44% | 13% | 20% | 50% |
| TB-profiler |  | 58% | 83% | 62% | 100% | 95% | 18% | 91% | 60% | 65% | 73% | 77% | 32% | 54% |
| WG-XGB | NPV | 99% | 99% | 99% | **99%** | **98%** | 98% | 99% | 99% | 99% | 98% | 99% | **96%** | **99%** |
| WG-ANN |  | 99% | 99% | 99% | 99% | 97% | 96% | 91% | 98% | 74% | 97% | 99% | 95% | 98% |
| TB-profiler |  | 99% | 99% | 100% | 98% | 96% | 98% | 99% | 99% | 99% | 98% | 99% | 95% | 97% |
| WG-XGB | Accuracy | **97%** | **99%** | 97% | 98% | **98%** | 76% | 95% | **98%** | **99%** | **97%** | 95% | 88% | 96% |
| WG-ANN |  | 94% | 96% | 93% | 93% | 96% | 67% | 54% | 87% | 98% | 88% | 79% | 87% | 96% |
| TB-profiler |  | 94% | 98% | 97% | 98% | 95% | 81% | 97% | 96% | 98% | 95% | 98% | 91% | 96% |

**Supplementary Table S4: Comparison of model predictive performance between imbalanced and balanced BV-BRC hold out dataset:** The performance metrics including sn (sensitivity)/recall, sp (specificity), MCC, Precision/PPV, NPV, F1 score, and accuracy, were evaluated for both imbalanced and balanced test datasets using WG-XGB and WG-ANN models. In the presented table, instances where WG-XGB demonstrated identical performance across imbalanced and balanced datasets are highlighted in bolded blue while for WG-ANN, results are emphasized in bold green. R= Resistant, S=Susceptible

| **Drug** | **R** | **S** | **Dataset** | **Sn/Recall** | **Sp** | **MCC** | **Precision/PPV** | **NPV** | **F1 score** | **Accuracy** |
| --- | --- | --- | --- | --- | --- | --- | --- | --- | --- | --- |
| INH | 288 | 3186 | WG-XGB imbalanced | **86%** | 98% | 0.81 | 79% | 99% | 0.83 | 97% |
|  |  |  | WG-ANN imbalanced | **90%** | 94% | 0.69 | 58% | 99% | 0.70 | 94% |
|  | 288 | 288 | WG-XGB balanced | **86%** | 97% | 0.83 | 97% | 87% | 0.91 | 92% |
|  |  |  | WG-ANN balanced | **90%** | 93% | 0.83 | 93% | 90% | 0.91 | 91% |
| EMB | 200 | 3642 | WG-XGB imbalanced | **90%** | **99%** | 0.91 | 93% | 99% | 0.91 | 99% |
|  |  |  | WG-ANN imbalanced | **92%** | 97% | 0.74 | 63% | 99% | 0.75 | 96% |
|  | 200 | 200 | WG-XGB balanced | **90%** | **99%** | 0.88 | 99% | 91% | 0.93 | 94% |
|  |  |  | WG-ANN balanced | **92%** | 95% | 0.88 | 95% | 92% | 0.94 | 94% |
| RIF | 278 | 5178 | WG-XGB imbalanced | **90%** | 97% | 0.74 | 64% | 99% | 0.75 | 97% |
|  |  |  | WG-ANN imbalanced | **93%** | 92% | 0.57 | 40% | 99% | 0.55 | 93% |
|  | 278 | 278 | WG-XGB balanced | **90%** | 98% | 0.88 | 93% | 90% | 0.94 | 94% |
|  |  |  | WG-ANN balanced | **93%** | 93% | 0.85 | 93% | 93% | 0.93 | 93% |
| PZA | 230 | 1796 | WG-XGB imbalanced | **95%** | **99%** | **0.94** | 99% | 99% | 0.95 | 98% |
|  |  |  | WG-ANN imbalanced | **81%** | 95% | 0.71 | 91% | 99% | 0.73 | 93% |
|  | 230 | 230 | WG-XGB balanced | **95%** | **99%** | **0.94** | 99% | 95% | 0.97 | 97% |
|  |  |  | WG-ANN balanced | **81%** | 93% | 0.75 | 93% | 83% | 0.86 | 87% |
| STM | 235 | 916 | WG-XGB imbalanced | **91%** | **99%** | 0.94 | 96% | 98% | **0.95** | 98% |
|  |  |  | WG-ANN imbalanced | **90%** | **98%** | 0.86 | 96% | 97% | 0.90 | 96% |
|  | 235 | 235 | WG-XGB balanced | **91%** | **99%** | 0.91 | 99% | 92% | **0.95** | 95% |
|  |  |  | WG-ANN balanced | **90%** | **98%** | 0.88 | 98% | 91% | 0.94 | 94% |
| ETH | 63 | 1036 | WG-XGB imbalanced | **79%** | 77% | 0.29 | 17% | 98% | 0.27 | 76% |
|  |  |  | WG-ANN imbalanced | **49%** | 68% | 0.08 | 9% | 96% | 0.14 | 67% |
|  | 63 | 63 | WG-XGB balanced | **79%** | 68% | 0.48 | 71% | 76% | 0.75 | 74% |
|  |  |  | WG-ANN balanced | **49%** | 65% | 0.14 | 58% | 56% | 0.53 | 57% |
| OFLX | 100 | 890 | WG-XGB imbalanced | **89%** | **95%** | 0.77 | 67% | 99% | 0.78 | 95% |
|  |  |  | WG-ANN imbalanced | **52%** | 55% | 0.04 | 11% | 91% | 0.18 | 54% |
|  | 100 | 100 | WG-XGB balanced | **89%** | **95%** | 0.84 | 95% | 90% | 0.91 | 92% |
|  |  |  | WG-ANN balanced | **52%** | 56% | 0.06 | 54% | 54% | 0.51 | 53% |
| KAN | 38 | 740 | WG-XGB imbalanced | **79%** | 99% | 0.87 | 60% | 99% | **0.87** | 98% |
|  |  |  | WG-ANN imbalanced | **65%** | 88% | 0.34 | 22% | 98% | 0.33 | 87% |
|  | 38 | 38 | WG-XGB balanced | **79%** | 97% | 0.77 | 96% | 82% | **0.87** | 88% |
|  |  |  | WG-ANN balanced | **65%** | 87% | 0.54 | 83% | 71% | 0.74 | 76% |
| CAP | 38 | 587 | WG-XGB imbalanced | **89%** | 100% | 0.94 | **99%** | 99% | 0.94 | 99% |
|  |  |  | WG-ANN imbalanced | **87%** | 98% | 0.78 | 74% | 74% | 0.84 | 98% |
|  | 38 | 38 | WG-XGB balanced | **89%** | 99% | 0.90 | **99%** | 90% | 0.94 | 94% |
|  |  |  | WG-ANN balanced | **87%** | 99% | 0.87 | 99% | 88% | 0.93 | 93% |
| AMI | 62 | 596 | WG-XGB imbalanced | **77%** | 99% | 0.81 | 90% | 98% | 0.85 | 97% |
|  |  |  | WG-ANN imbalanced | **70%** | 91% | 0.50 | 45% | 97% | 0.54 | 88% |
|  | 62 | 62 | WG-XGB balanced | **77%** | 98% | 0.77 | **97%** | 81% | 0.86 | 88% |
|  |  |  | WG-ANN balanced | **70%** | 90% | 0.61 | **88%** | 75% | 0.77 | 80% |
| MXF | 35 | 831 | WG-XGB imbalanced | **83%** | 95% | 0.59 | 44% | 99% | 0.58 | 95% |
|  |  |  | WG-ANN imbalanced | 72% | 79% | 0.24 | 13% | 99% | 0.21 | 79% |
|  | 35 | 35 | WG-XGB balanced | **83%** | 97% | 0.81 | 97% | 85% | 0.89 | 90% |
|  |  |  | WG-ANN balanced | 71% | 85% | 0.57 | 83% | 75% | 0.77 | 79% |
| CYCLO | 26 | 362 | WG-XGB imbalanced | **54%** | 90% | 0.32 | 29% | 96% | 0.38 | 88% |
|  |  |  | WG-ANN imbalanced | **27%** | **92%** | 0.16 | 20% | 95% | 0.22 | 88% |
|  | 26 | 26 | WG-XGB balanced | **54%** | 92% | 0.50 | 87% | 67% | 0.66 | 73% |
|  |  |  | WG-ANN balanced | **27%** | **92%** | 0.25 | 77% | 56% | 0.4 | 60% |
| PAS | 10 | 283 | WG-XGB imbalanced | 64% | 97% | 0.52 | 46% | 99% | 0.54 | 96% |
|  |  |  | WG-ANN imbalanced | **54%** | 98% | 0.48 | 50% | 98% | 0.47 | 96% |
|  | 10 | 10 | WG-XGB balanced | 60% | 90% | 0.65 | 86% | 70% | 0.74 | 80% |
|  |  |  | WG-ANN balanced | **54%** | 91% | 0.48 | 86% | 66% | 0.66 | 73% |

**Supplementary Table S5: Model evaluation on the CRyPTIC dataset and comparison with TB-Profiler:** Model evaluation of both WG-XGB and WG-ANN on CRyPTIC dataset and comparison with TB-profiler based on precision/PPV, NPV, and accuracy. Bolded numbers indicate our model performing better than TB-profiler. Given that all training models were exclusively built using the BV-BRC dataset, the utilization of the entire CRyPTIC dataset as a hold out test dataset aimed to evaluate model diversity and reproducibility. R= Resistant, S=Susceptible

| Drugs | | INH | EMB | RIF | ETH | KAN | AMI | MXF |
| --- | --- | --- | --- | --- | --- | --- | --- | --- |
| R | | 5908 | 2261 | 4684 | 1727 | 1121 | 883 | 1724 |
| S | | 6161 | 8337 | 7414 | 9431 | 11008 | 11188 | 10565 |
| WG-XGB | Precision/PPV | 96% | 71% | **92%** | 65% | 51% | **88%** | 46% |
| WG-ANN |  | 90% | 76% | 86% | 15% | 43% | 33% | 21% |
| TB-profiler |  | 97% | 76% | 91% | 73% | 64% | 75% | 70% |
| WG-XGB | NPV | 86% | 97% | 95% | 95% | 97% | 98% | 97% |
| WG-ANN |  | 87% | 98% | 84% | 84% | 95% | 95% | 92% |
| TB-profiler |  | 93% | 98% | 96% | 95% | 97% | 98% | 97% |
| WG-XGB | Accuracy | 85% | 90% | 94% | 90% | 91% | **97%** | 84% |
| WG-ANN |  | 88% | 92% | 92% | 53% | 90% | 90% | 58% |
| TB-profiler |  | 94% | 92% | 94% | 92% | 94% | 96% | 93% |

**Supplementary Table S6:** **Comparison of model predictive performance between imbalanced and balanced CRyPTIC dataset:** The performance metrics including sn (sensitivity)/recall, sp (specificity), MCC, Precision/PPV, NPV, F1 score, and accuracy, were evaluated for both imbalanced and balanced test datasets using WG-XGB and WG-ANN models. In the presented table, instances where WG-XGB demonstrated identical performance across imbalanced and balanced datasets are highlighted in bold blue while for WG-ANN, results are emphasized in bold green. R= Resistant, S=Susceptible.

| **Drug** | **R** | **S** | **Dataset** | **Sn/Recall** | **Sp** | **MCC** | **Precision/PPV** | **NPV** | **F1 score** | **Accuracy** |
| --- | --- | --- | --- | --- | --- | --- | --- | --- | --- | --- |
| INH | 5908 | 6161 | WG-XGB imbalanced | **84%** | 96% | 0.81 | 96% | 86% | 0.90 | 91% |
|  |  |  | WG-ANN imbalanced | **86%** | 91% | 0.85 | 90% | 87% | 0.93 | 93% |
|  | 5908 | 5908 | WG-XGB balanced | **84%** | 84% | 0.69 | 85% | 85% | 0.85 | 85% |
|  |  |  | WG-ANN balanced | **86%** | 85% | 0.72 | 85% | 86% | 0.86 | 86% |
| EMB | 2261 | 8337 | WG-XGB imbalanced | **91%** | 90% | 0.74 | 71% | 97% | 0.80 | 90% |
|  |  |  | WG-ANN imbalanced | **92%** | 92% | 0.78 | 76% | 98% | 0.83 | 92% |
|  | 2261 | 2261 | WG-XGB balanced | **91%** | 92% | 0.83 | 92% | 91% | 0.91 | 91% |
|  |  |  | WG-ANN balanced | **92%** | 93% | 0.85 | 93% | 92% | 0.93 | 93% |
| RIF | 4684 | 7414 | WG-XGB imbalanced | **92%** | 95% | 0.87 | 92% | 95% | **0.92** | 94% |
|  |  |  | WG-ANN imbalanced | **94%** | 90% | 0.83 | 86% | 96% | 0.90 | 92% |
|  | 4684 | 4684 | WG-XGB balanced | **92%** | 93% | 0.85 | 93% | 92% | **0.92** | 93% |
|  |  |  | WG-ANN balanced | **94%** | 94% | 0.88 | 94% | 94% | 0.94 | 94% |
| ETH | 1727 | 9431 | WG-XGB imbalanced | **72%** | 93% | 0.61 | 65% | 95% | 0.68 | 90% |
|  |  |  | WG-ANN imbalanced | **43%** | 55% | 0.01 | 15% | 84% | 0.22 | 53% |
|  | 1727 | 1727 | WG-XGB balanced | **72%** | 90% | 0.64 | 88% | 76% | 0.80 | 81% |
|  |  |  | WG-ANN balanced | **43%** | 76% | 0.21 | 65% | 57% | 0.52 | 60% |
| KAN | 1121 | 11008 | WG-XGB imbalanced | **72%** | 93% | 0.54 | 51% | 97% | 0.58 | 91% |
|  |  |  | WG-ANN imbalanced | **51%** | **93%** | 0.40 | 43% | 95% | 0.46 | 90% |
|  | 1121 | 1121 | WG-XGB balanced | **72%** | 95% | 0.68 | 94% | 77% | 0.81 | 83% |
|  |  |  | WG-ANN balanced | **51%** | **93%** | 0.48 | 88% | 65% | 0.64 | 72% |
| AMI | 883 | 11188 | WG-XGB imbalanced | **74%** | 99% | 0.80 | 85% | 98% | 0.81 | 97% |
|  |  |  | WG-ANN imbalanced | **36%** | 94% | 0.26 | 33% | 95% | 0.35 | 90% |
|  | 883 | 883 | WG-XGB balanced | **74%** | 96% | 0.71 | 95% | 80% | 0.83 | 85% |
|  |  |  | WG-ANN balanced | **36%** | 93% | 0.36 | 84% | 60% | 0.50 | 64% |
| MXF | 1724 | 10565 | WG-XGB imbalanced | **83%** | 84% | 0.53 | 46% | 97% | 0.60 | 84% |
|  |  |  | WG-ANN imbalanced | **68%** | 57% | 0.17 | 21% | 92% | 0.32 | 58% |
|  | 1724 | 1724 | WG-XGB balanced | **83%** | 97% | 0.81 | 97% | 85% | 0.89 | 90% |
|  |  |  | WG-ANN balanced | **68%** | 82% | 0.51 | 80% | 72% | 0.73 | 75% |

**Supplementary Table S7: Total number of important mutations extracted by XGBoost (xgb) feature score and SHAP method.** For MXF, OFLX, and ETH, the XGBoost important feature extraction module was exclusively employed, as the performance of WG-ANN in these models is suboptimal.

| Drugs | No. of predicted drug resistant associated features (xgb) | No. of predicted drug resistant associated features (SHAP) | No. of predicted drug resistant associated features (combined) |
| --- | --- | --- | --- |
| INH | 45 | 34 | 46 |
| EMB | 37 | 35 | 36 |
| RIF | 32 | 36 | 36 |
| PZA | 99 | 90 | 91 |
| STM | 33 | 44 | 48 |
| ETH | 6 | NA | 6 |
| OFLX | 9 | NA | 9 |
| KAN | 9 | 20 | 21 |
| CAP | 7 | 14 | 15 |
| AMI | 2 | 7 | 7 |
| MXF | 5 | NA | 5 |
| CYCLO | 9 | 23 | 25 |
| PAS | 7 | 22 | 24 |

**Supplementary Table S8:** **Mutation wise list of WHO 2021 and WHO 2023 and XAI predicted mutations:** for OFLX, PAS, CYCLO mutation information were not there both in WHO 2021 and 2023. For these three drugs, all mutations were considered as new gene mutations.

| Drugs |  | Assoc w R | Assoc w R - Interim | Uncertain significance | Not assoc w R | Not assoc w R - Interim | Known gene new mut | New gene mutation | Co-occurrant mutation |
| --- | --- | --- | --- | --- | --- | --- | --- | --- | --- |
| INH | WHO 2021 | 4 | 118 | 2252 | 22 | 6 | NA | NA | NA |
|  | WHO 2023 | 8 | 135 | 5404 | 57 | 1682 | NA | NA | NA |
|  | ML model | 2 | 1 | 14 | 0 | 0 | 2 | 10 | 17 |
| EMB | WHO 2021 | 14 | 1 | 2641 | 39 | 5 | NA | NA | NA |
|  | WHO 2023 | 13 | 0 | 4943 | 84 | 2078 | NA | NA | NA |
|  | ML model | 10 | 0 | 4 | 0 | 0 | 0 | 11 | 11 |
| RIF | WHO 2021 | 24 | 111 | 1550 | 28 | 0 | NA | NA | NA |
|  | WHO 2023 | 26 | 110 | 4484 | 84 | 2570 | NA | NA | NA |
|  | ML model | 17 | 0 | 0 | 0 | 0 | 3 | 10 | 6 |
| PZA | WHO 2021 | 105 | 233 | 775 | 15 | 16 | NA | NA | NA |
|  | WHO 2023 | 140 | 202 | 1465 | 25 | 740 | NA | NA | NA |
|  | ML model | 28 | 1 | 0 | 0 | 0 | 1 | 41 | 20 |
| STM | WHO 2021 | 12 | 166 | 1327 | 15 | 1 | NA | NA | NA |
|  | WHO 2023 | 15 | 144 | 2342 | 21 | 539 | NA | NA | NA |
|  | ML model | 5 | 1 | 5 | 1 | 0 | 0 | 33 | 3 |
| ETH | WHO 2021 | 4 | 327 | 1099 | 0 | 0 | NA | NA | NA |
|  | WHO 2023 | 6 | 281 | 1944 | 515 | 2 | NA | NA | NA |
|  | ML model | 1 | 1 | 0 | 0 | 0 | 0 | 3 | 1 |
| OFLX | WHO 2021 | NA | NA | NA | NA | NA | NA | NA | NA |
|  | WHO 2023 | NA | NA | NA | NA | NA | NA | NA | NA |
|  | ML model | NA | NA | NA | NA | NA | 0 | 9 | 0 |
| KAN | WHO 2021 | 7 | 1 | 594 | 12 | 1 | NA | NA | NA |
|  | WHO 2023 | 6 | 2 | 1862 | 14 | 356 | NA | NA | NA |
|  | ML model | 1 | 0 | 0 | 2 | 0 | 1 | 11 | 6 |
| CAP | WHO 2021 | 5 | 33 | 1009 | 20 | 0 | NA | NA | NA |
|  | WHO 2023 | 6 | 64 | 2273 | 57 | 254 | NA | NA | NA |
|  | ML model | 1 | 0 | 0 | 1 | 0 | 0 | 12 | 1 |
| AMI | WHO 2021 | 2 | 2 | 1543 | 24 | 0 | NA | NA | NA |
|  | WHO 2023 | 2 | 2 | 1772 | 70 | 344 | NA | NA | NA |
|  | ML model | 1 | 0 | 0 | 0 |  | 0 | 6 | 0 |
| MXF | WHO 2021 | 9 | 5 | 552 | 11 | 2 | NA | NA | NA |
|  | WHO 2023 | 10 | 8 | 1775 | 24 | 906 | NA | NA | NA |
|  | ML model | 3 | 0 | 0 | 0 | 0 | 0 | 2 | 0 |
| CYCLO | WHO 2021 | NA | NA | NA | NA | NA | NA | NA | NA |
|  | WHO 2023 | NA | NA | NA | NA | NA | NA | NA | NA |
|  | ML model | NA | NA | NA | NA | NA | 0 | 25 | 0 |
| PAS | WHO 2021 | NA | NA | NA | NA | NA | NA | NA | NA |
|  | WHO 2023 | NA | NA | NA | NA | NA | NA | NA | NA |
|  | ML model | NA | NA | NA | NA | NA | 0 | 23 | 1 |

**Supplementary Table S****9: WHO-endorsed list of genes associated with drug resistance in *Mycobacterium tuberculosis.***

| **Drugs** | **Genes for the sequences relevant to drug resistance** | **Total number of genes** |
| --- | --- | --- |
| INH | *ahpC, inhA, katG, mshA, ndh, Rv1258c, Rv2752c* | 7 |
| EMB | *embB, embC, embR, ubiA* | 5 |
| RIF | *rpoA, rpoB, rpoC, Rv2752c* | 4 |
| PZA | *clpC1, panD, pncA, PPE35, Rv1258c, Rv3236c* | 6 |
| STM | *gid, rpsL, rrs, Rv1258c, whiB6, whiB7* | 6 |
| ETH | *ethA, ethR, inhA, mshA, ndh, Rv3083* | 6 |
| KAN | *eis, rrs, whiB7* | 3 |
| CAP | *aftB, ccsA, fprA, rrs, tlyA, whiB6* | 6 |
| AMI | *aftB, ccsA, eis, fprA, rrs, whiB6, whiB7* | 7 |
| MXF | *gyrA, gyrB* | 2 |

**Supplementary Figure S1:** **Method of feature generation:** Using snippy variant calling tool all the mutations from drug resistance and susceptible were gathered and a unique mutation file was prepared. Applying filters, the final presence-absence matrix was prepared.


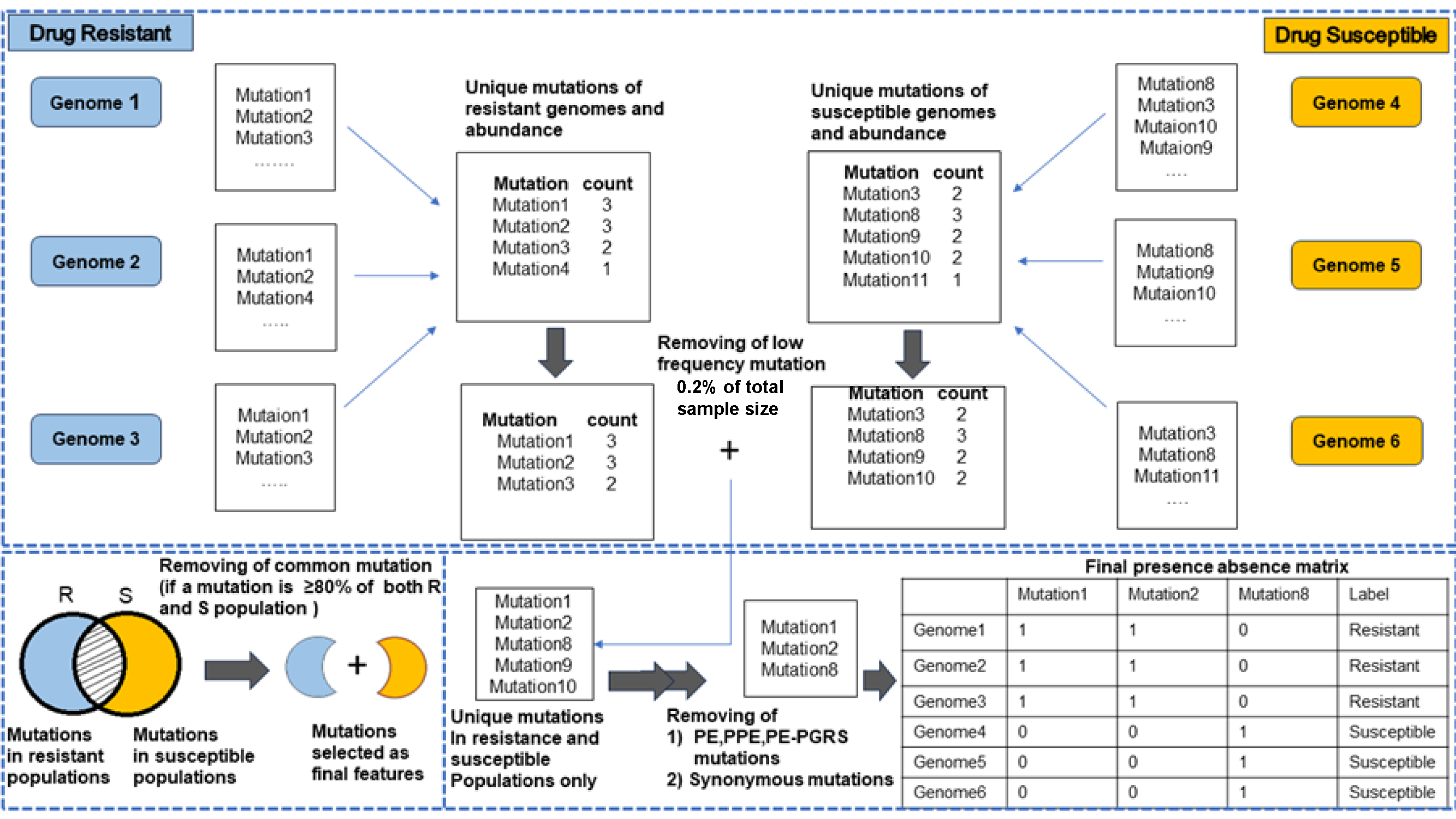


**Supplementary Figure S2**: **Important mutations were extracted using XGBoost and SHAP**. A cut-off of '0' was applied to XGBoost scores, and a cut-off of ‘0.01’ was applied to SHAP scores to select high-scoring mutations from each method. Because the scoring scales differed between the two methods, normalization was performed to bring the feature scores to a common scale before combining the scores for each mutation.
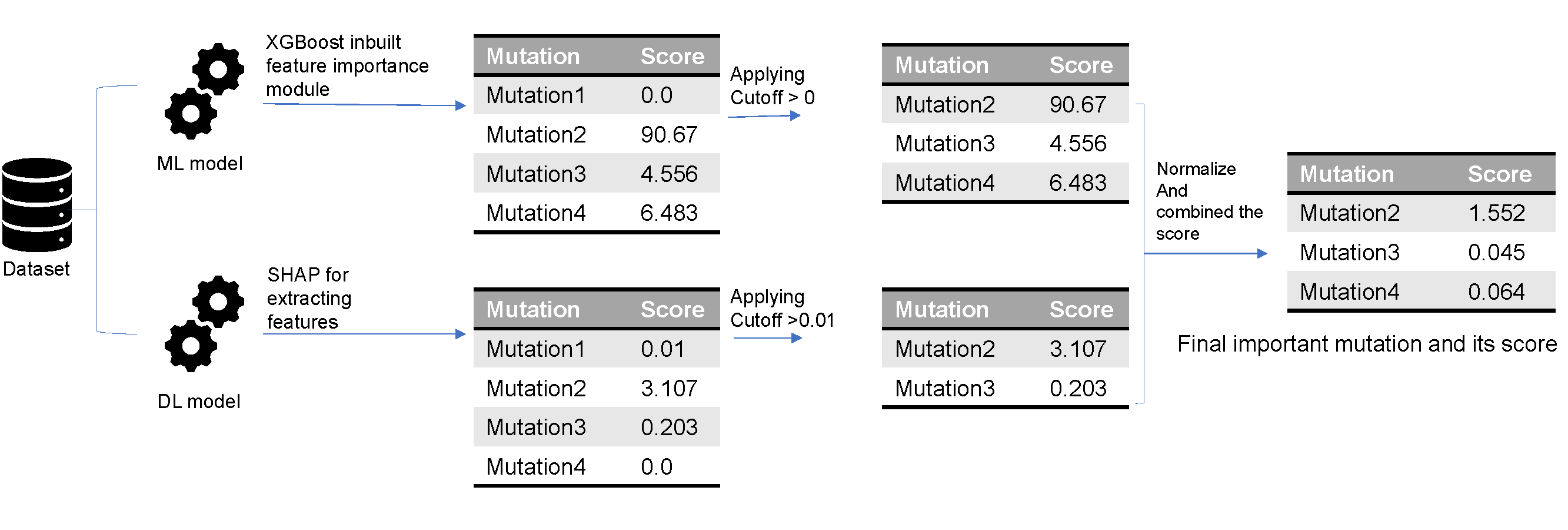


**Supplementary Figure S3:** **Comparison of predicted mutation with WHO mutation catalogue in terms of percentage:** New gene mutations refer to those not reported in the WHO mutation catalogue. In the case of OFLX, PAS, and CYCLO, as these drugs are not yet listed, all mutations are considered as new gene mutations**.** Percentage of overlap of each category of WHO mutations in the ML-predicted mutation list for **A.** INH, EMB, RIF; **B.** STM, PZA; **C.** KAN, AMI, CAP; **D.**MXF, OFLX, ETH; **E.** PAS, CYCLO**.**


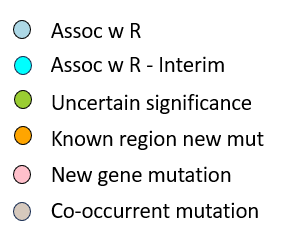


**A.**

| 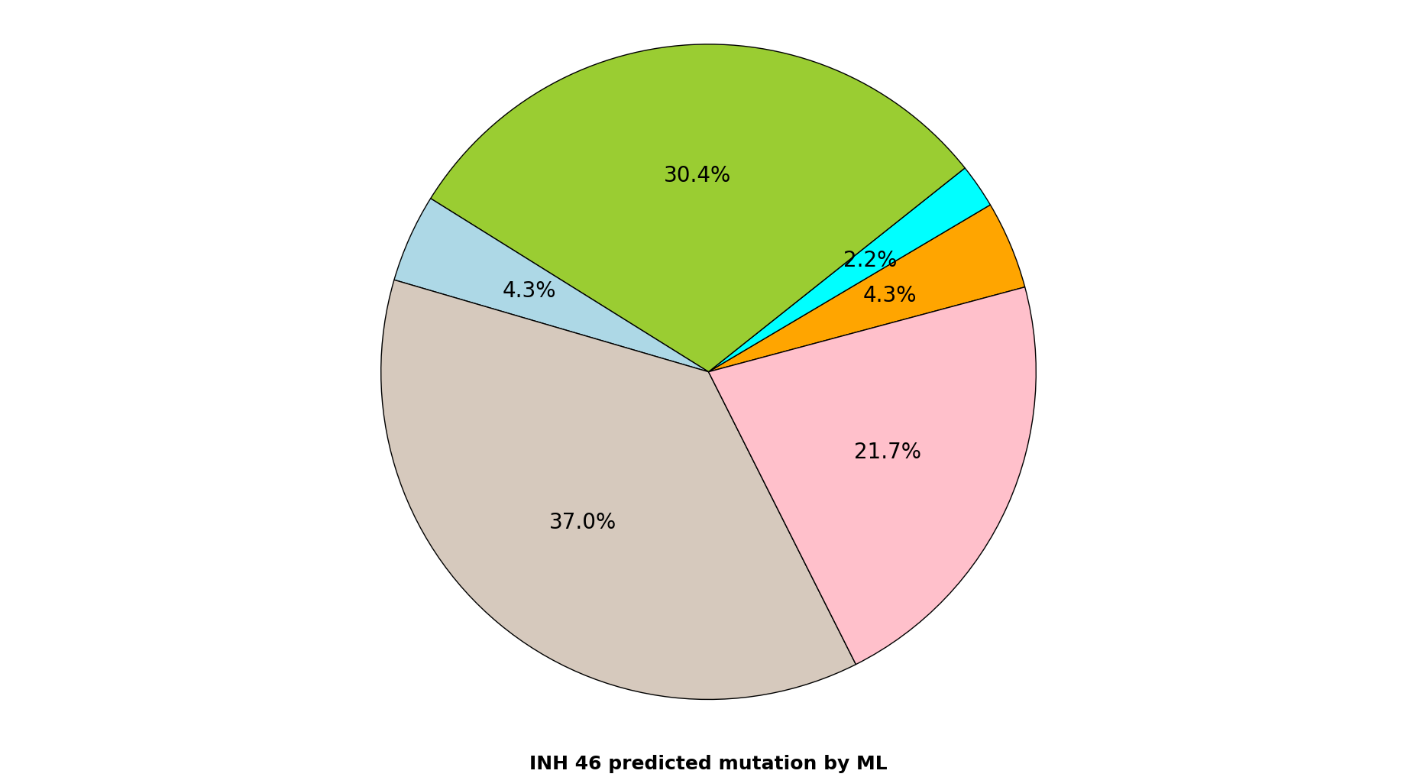 | 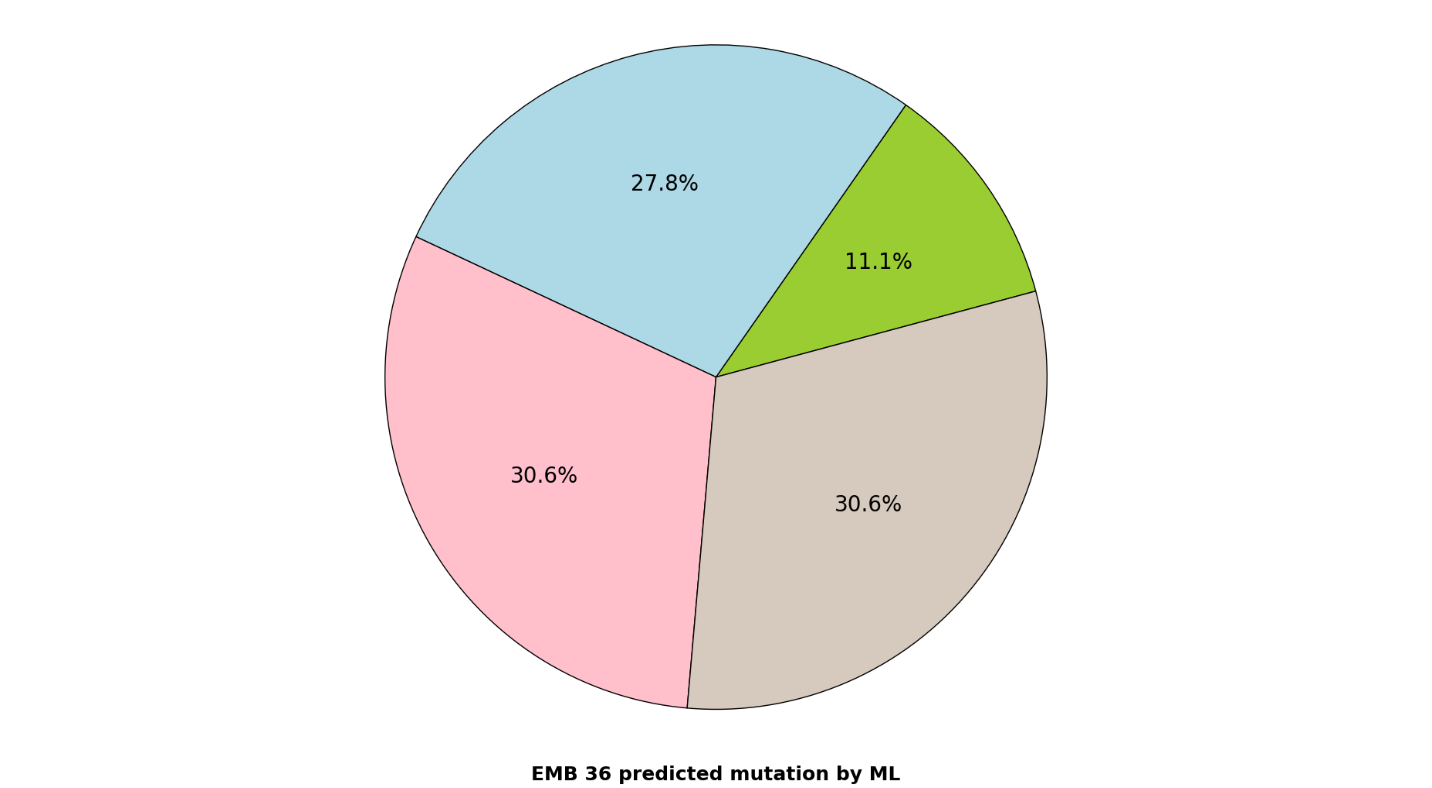 | 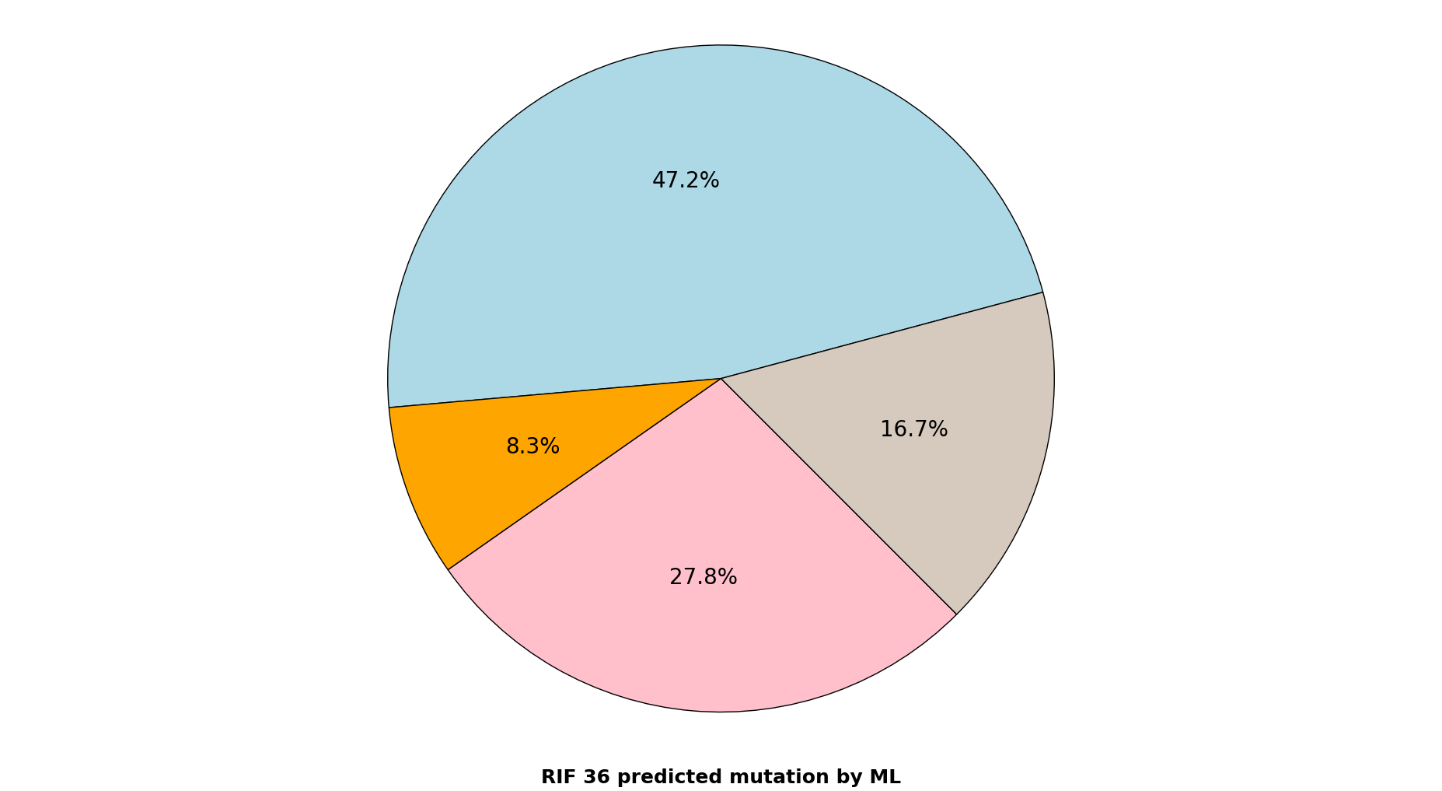 |
| --- | --- | --- |


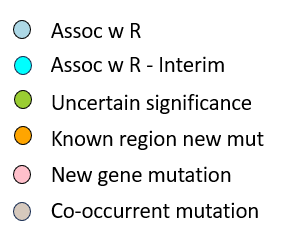


**B.**

| **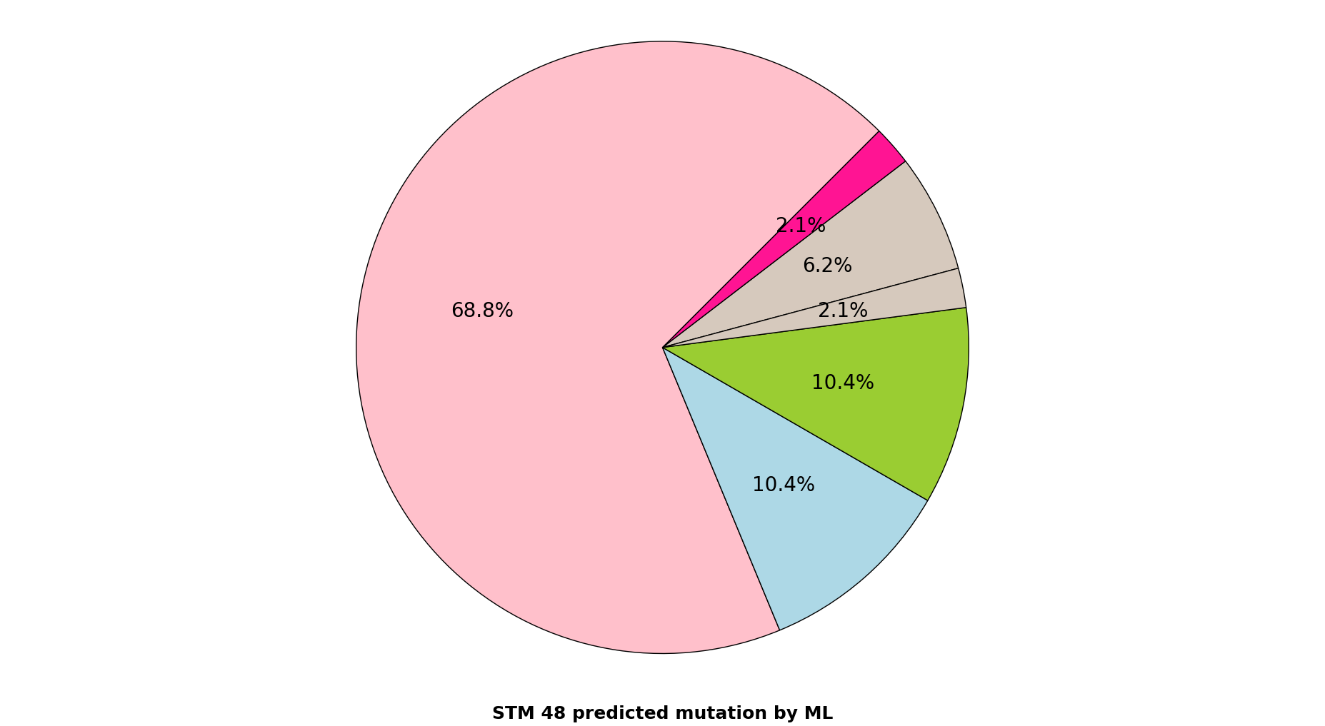** | **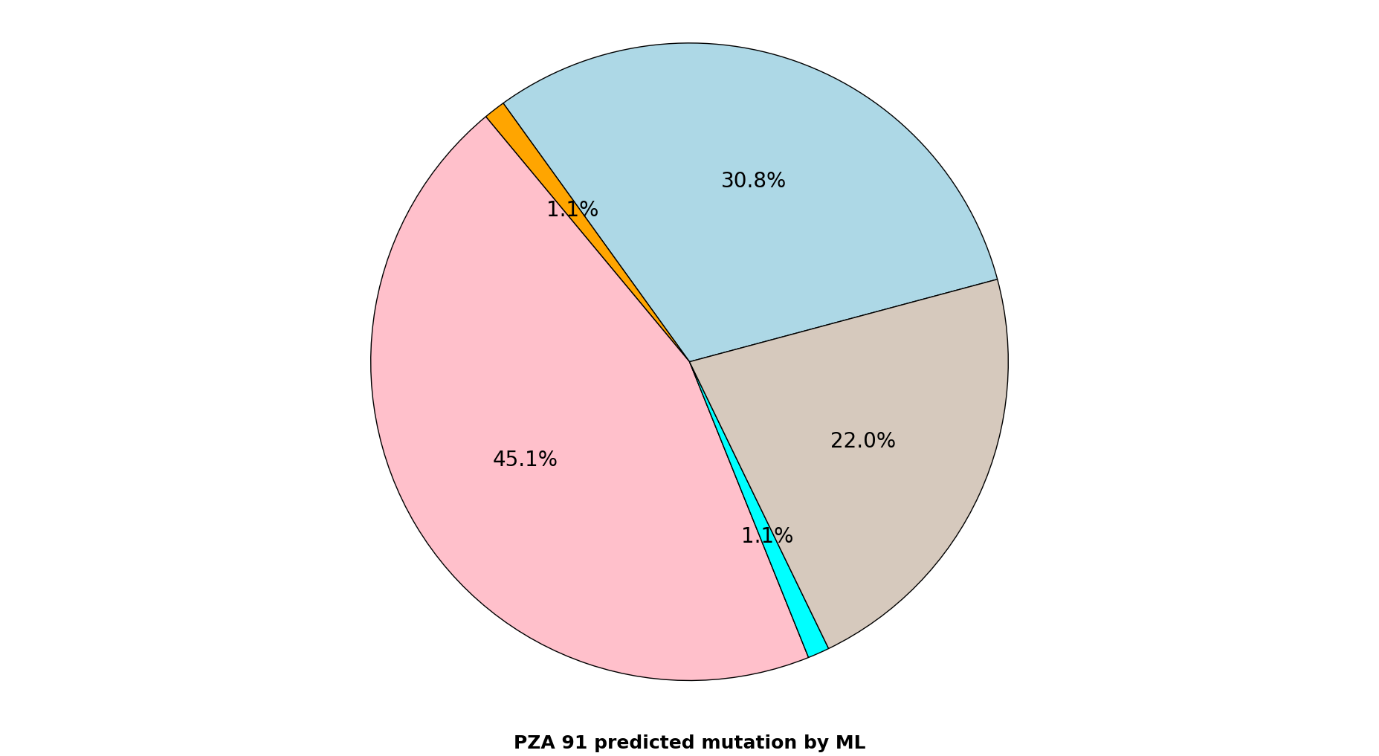** |
| --- | --- |


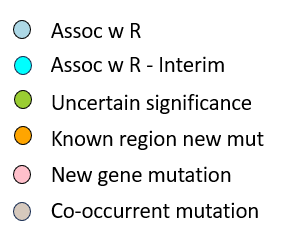


| **C.**  **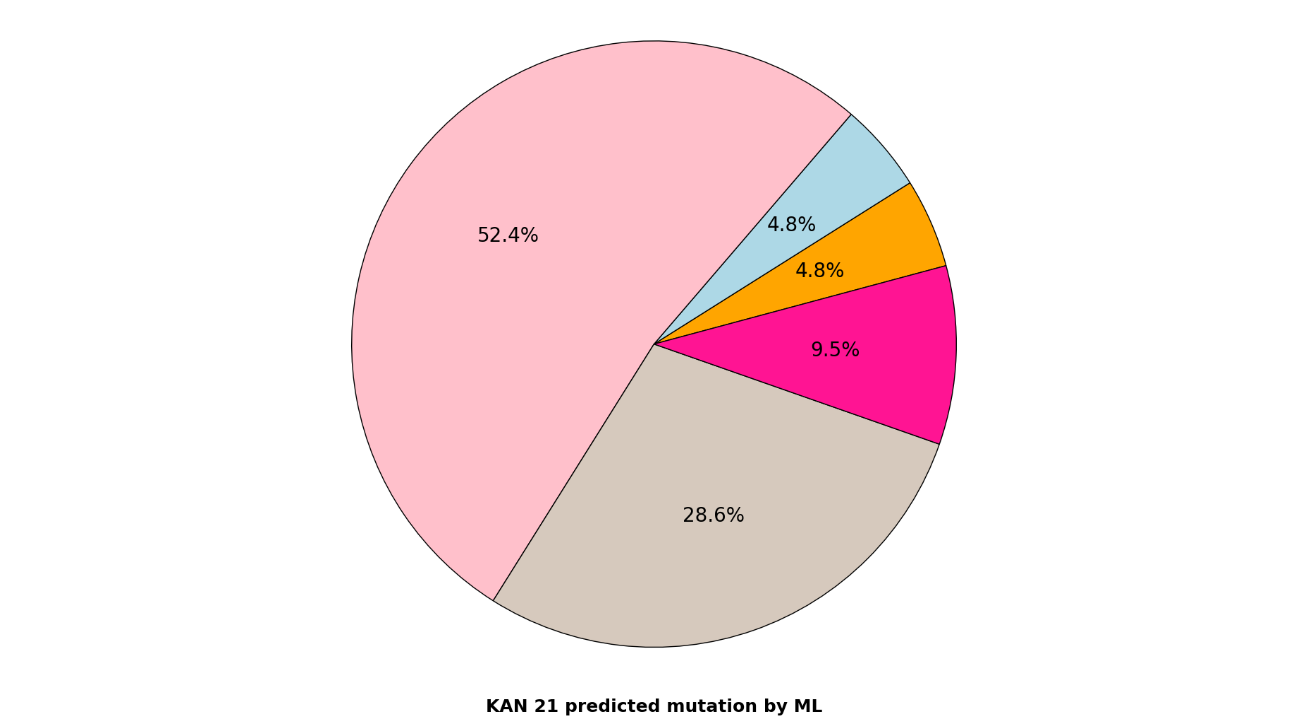** | **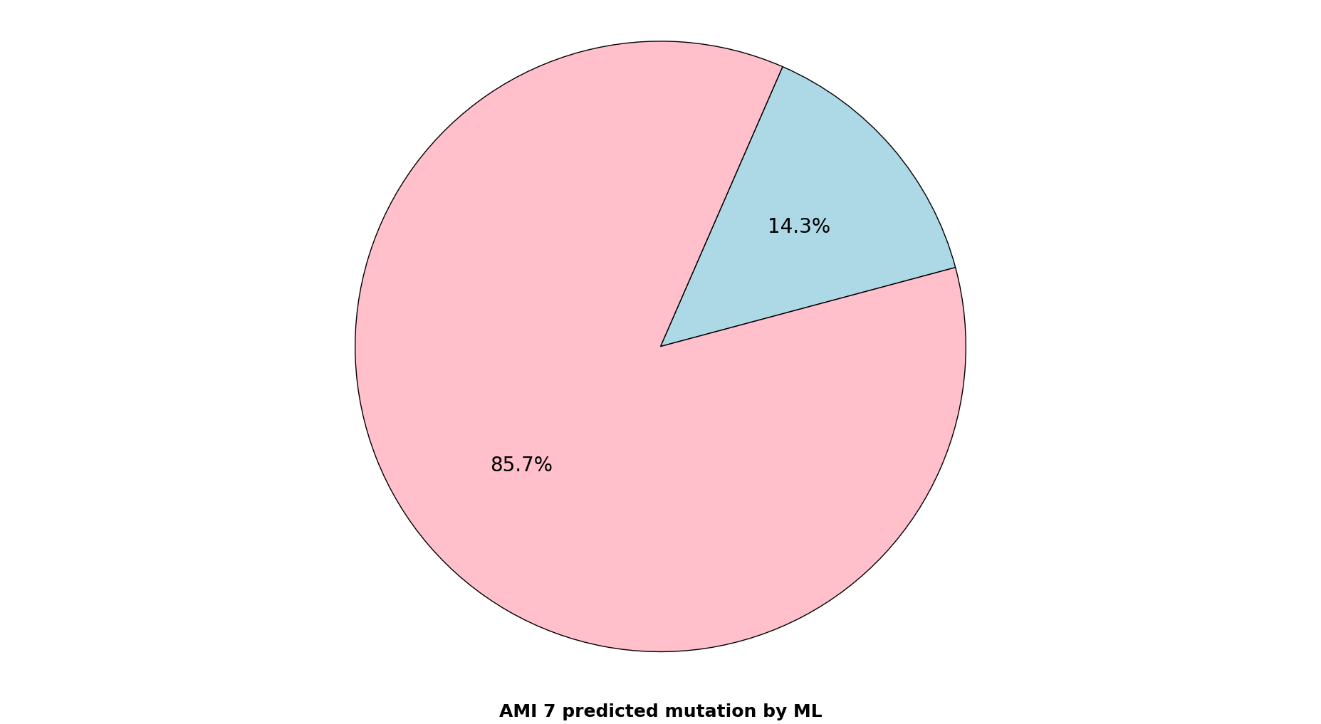** | 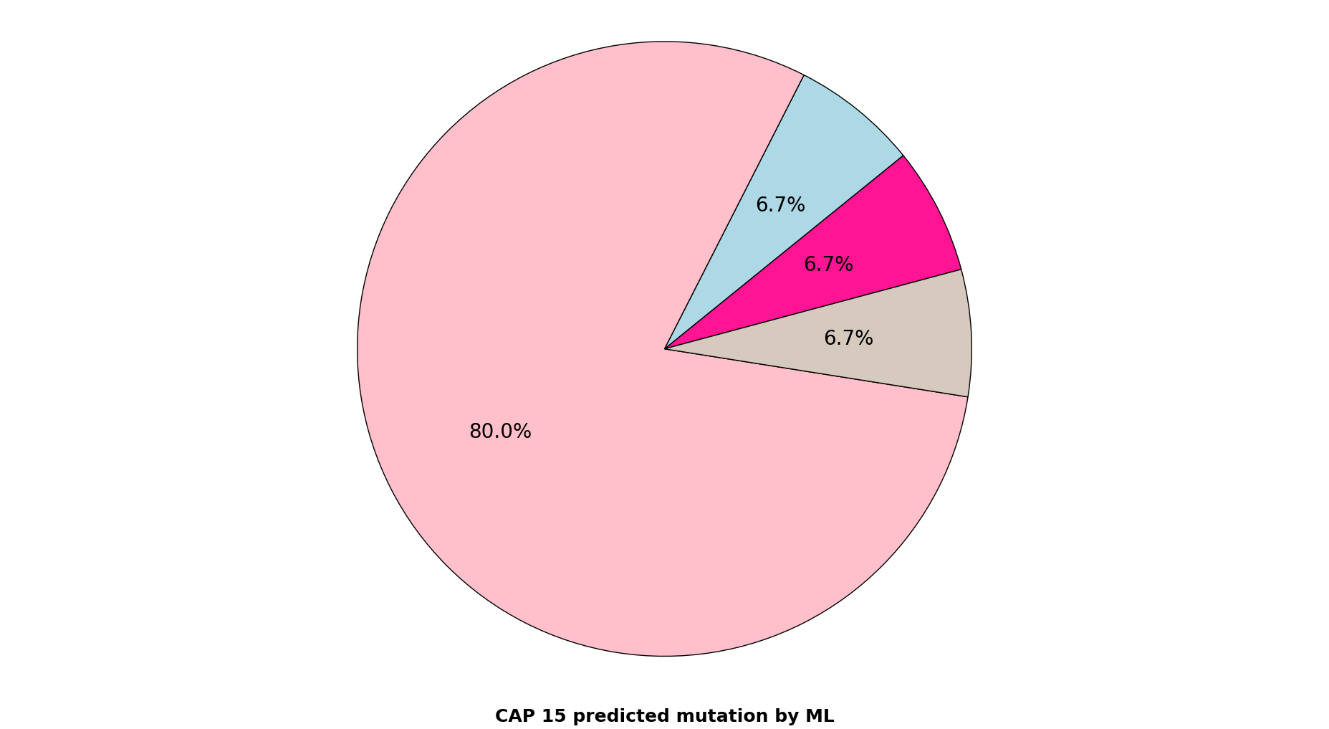 |
| --- | --- | --- |


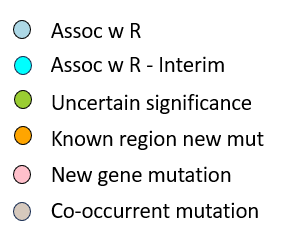


**D.**

| **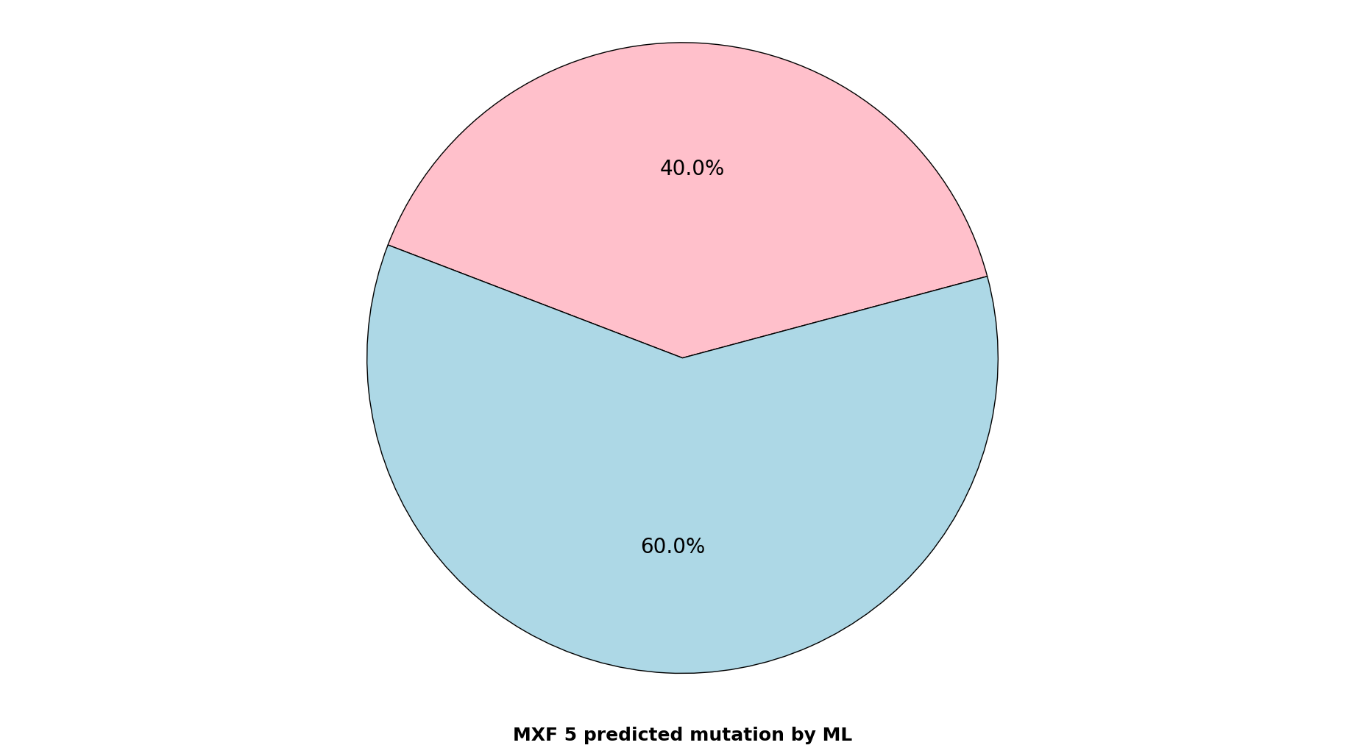** | **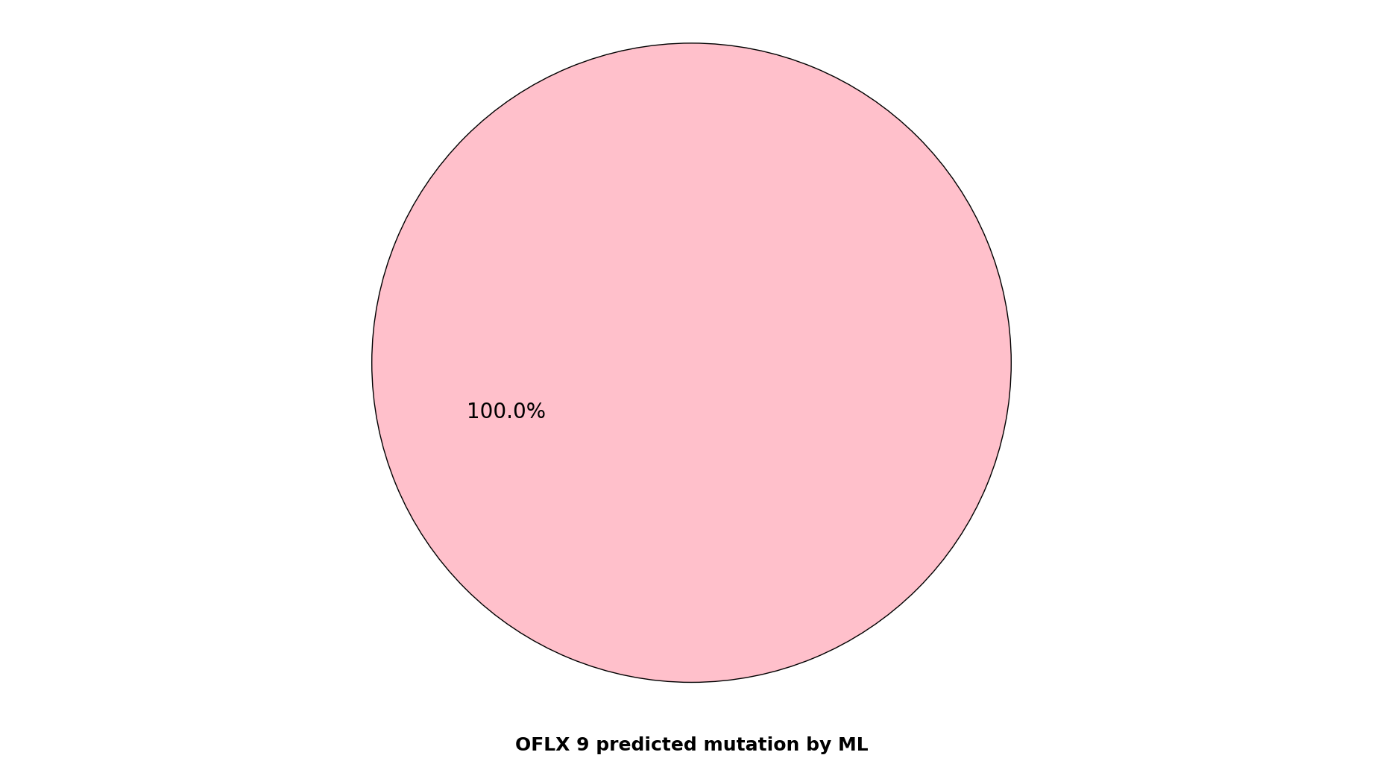** | **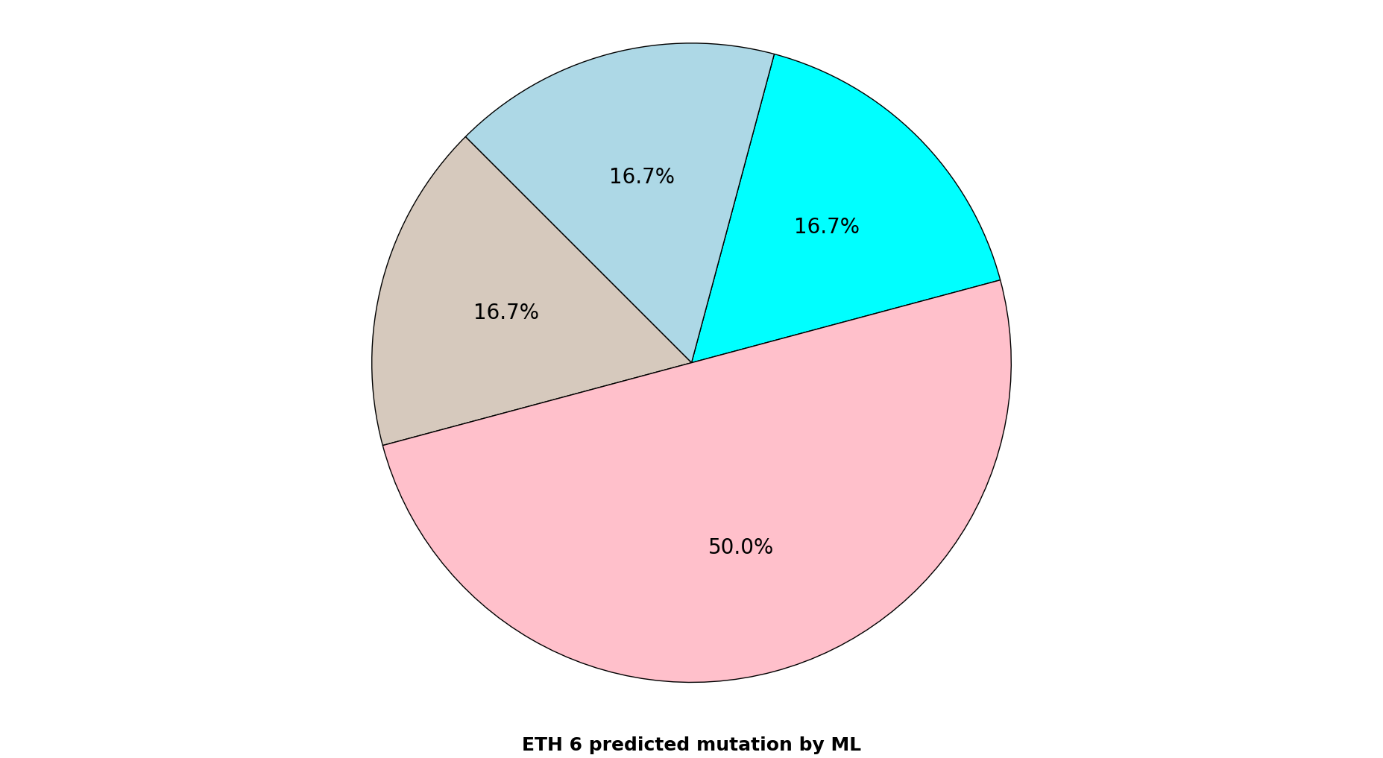** |
| --- | --- | --- |


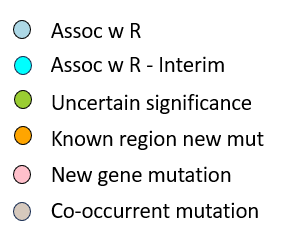


**E.**

| **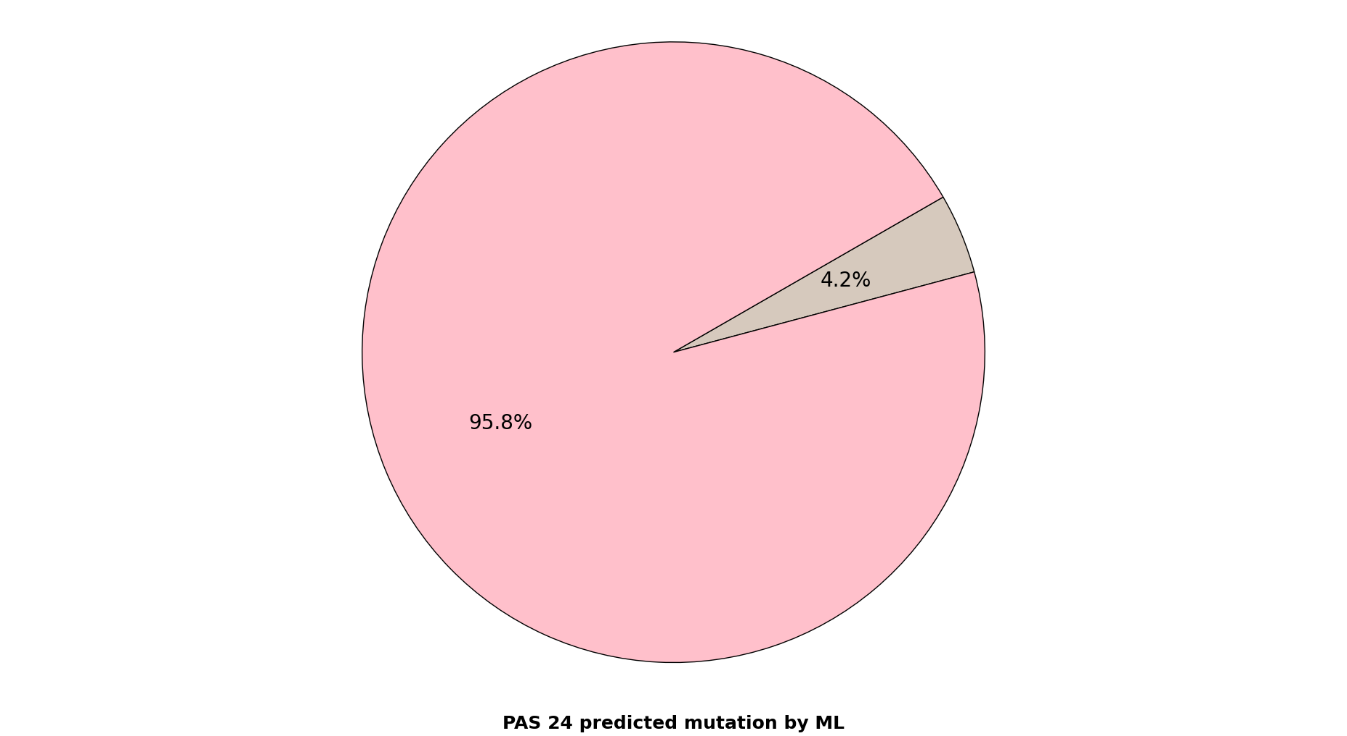** | **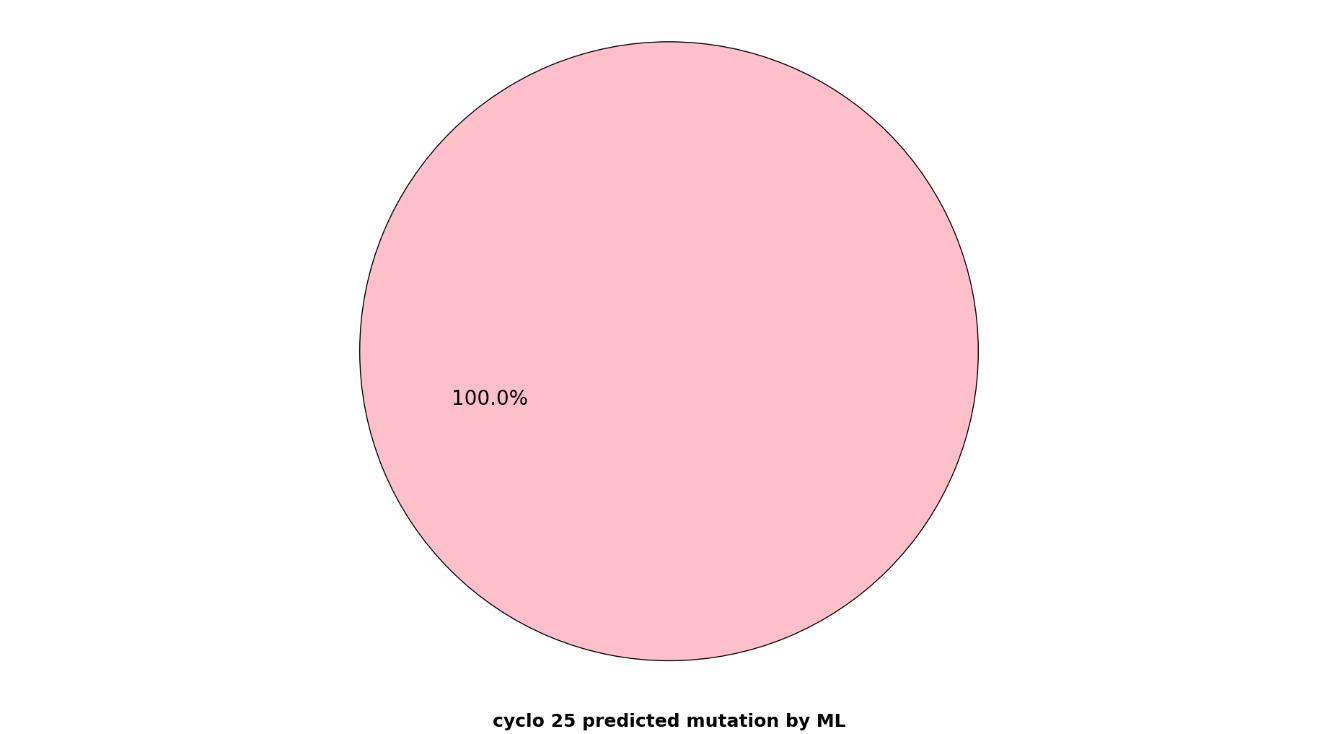** |
| --- | --- |

**Supplementary Figure S4: The mutation profiles of all 13 drugs, along with their respective abundance in both resistant and susceptible M. tb populations, are presented alongside the ML predicted scores.**  Assessment of each mutation for all the 11 drugs (**S4A**. Isoniazid; **S4B**. Pyrazinamide; **S4C** Rifampicin; **S4D.** Capreomycin; **S4E.** Amikacin; **S4F.** Kanamycin; **S4G.** Ethionamide; **S4H.** Moxifloxacin; **S4I.** Ofloxacin; **S4J.** Cycloserinee and **S4K.** PAS) in terms of the machine learning predicted score and relative abundance in both drug-resistant and susceptible *M.tb* populations. Each mutation is denoted by a distinct color of an asterisk (*), with each color representing a specific WHO gradation While arrow (^) in magenta and black colours indicated “New gene mutation” and “New mutation in known gene” respectively, predicted by our ML medthod.


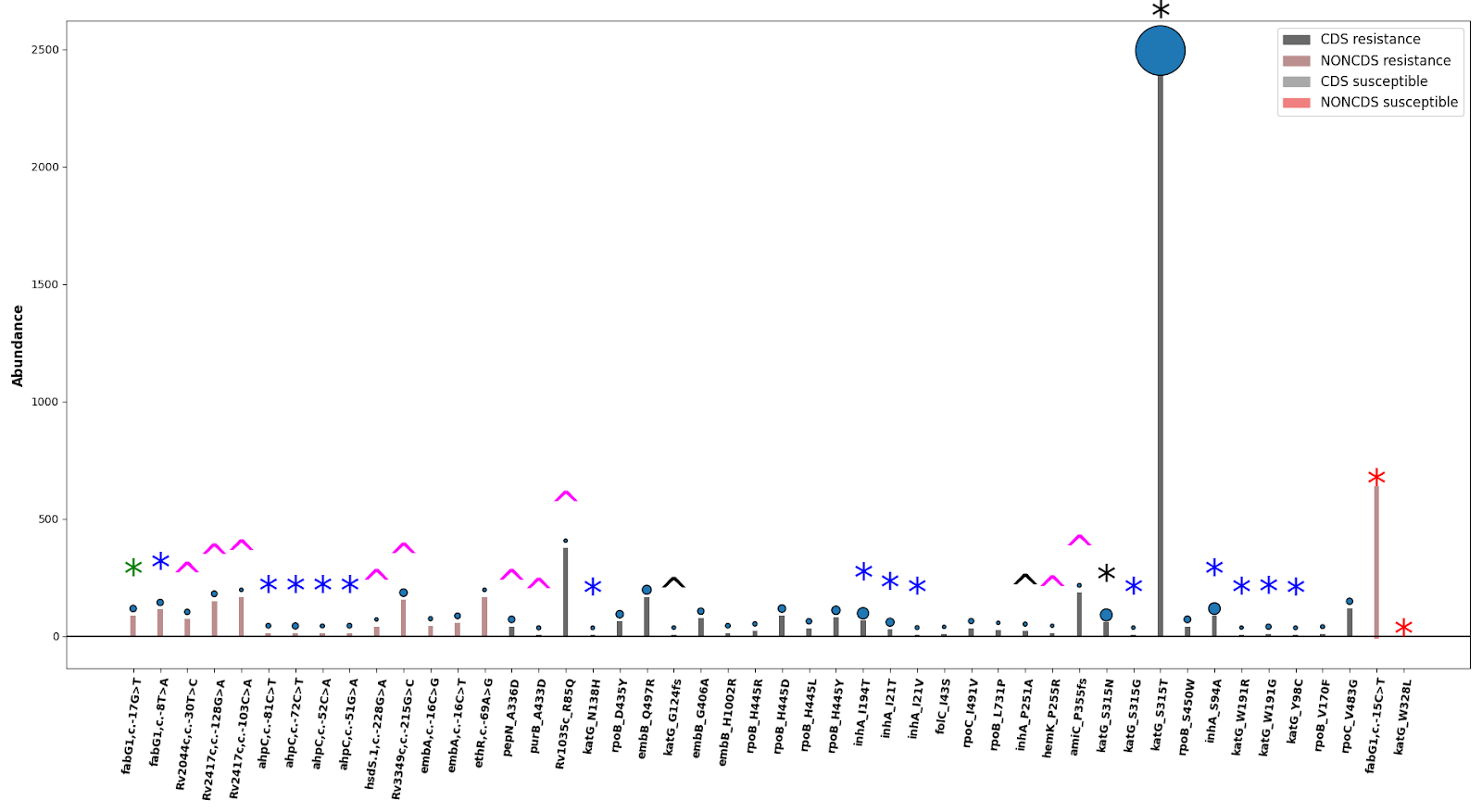

**Figure S4A: Isoniazid mutation profile**

**
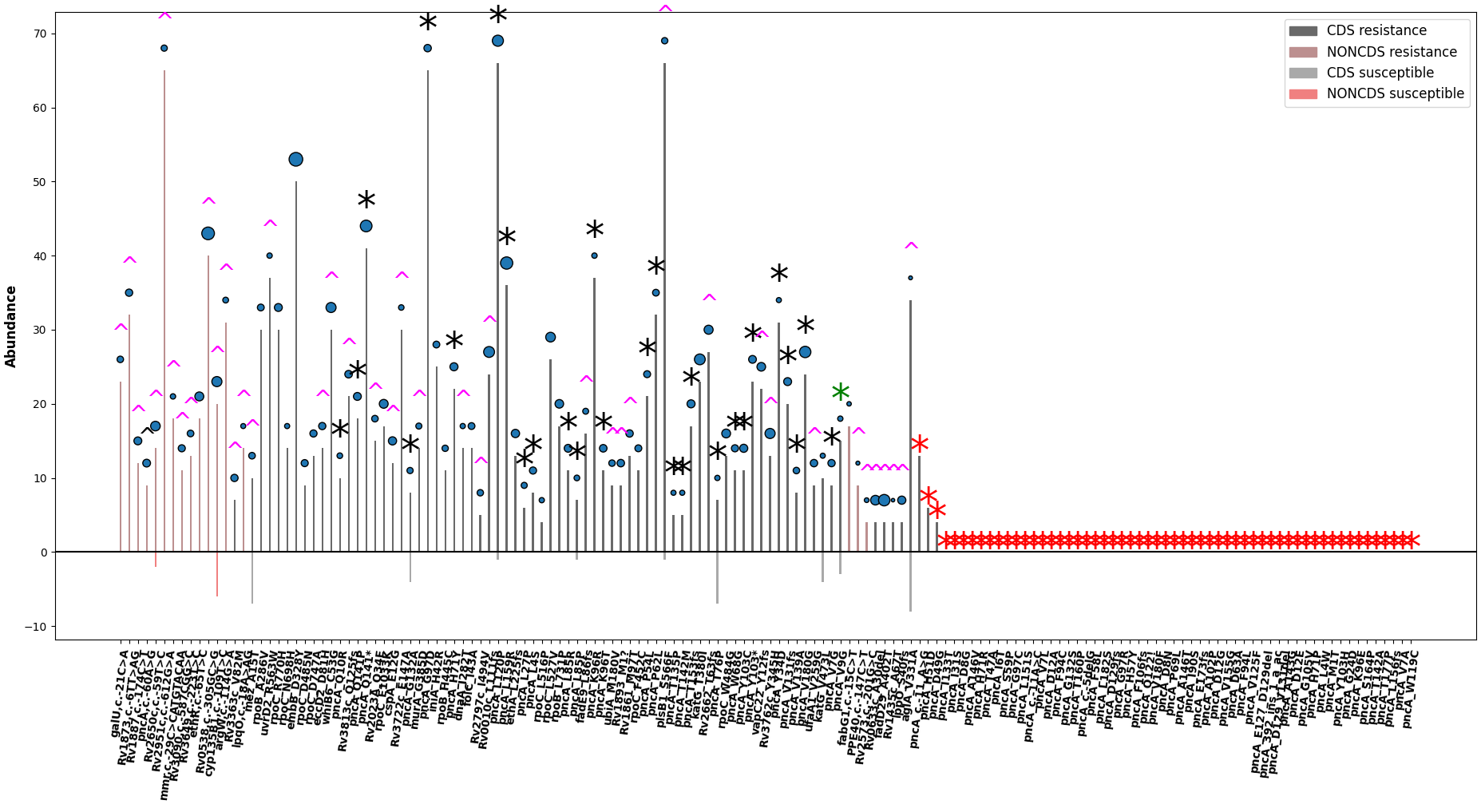
**

**Figure S4B: Pyrazinamide mutation profile**

**
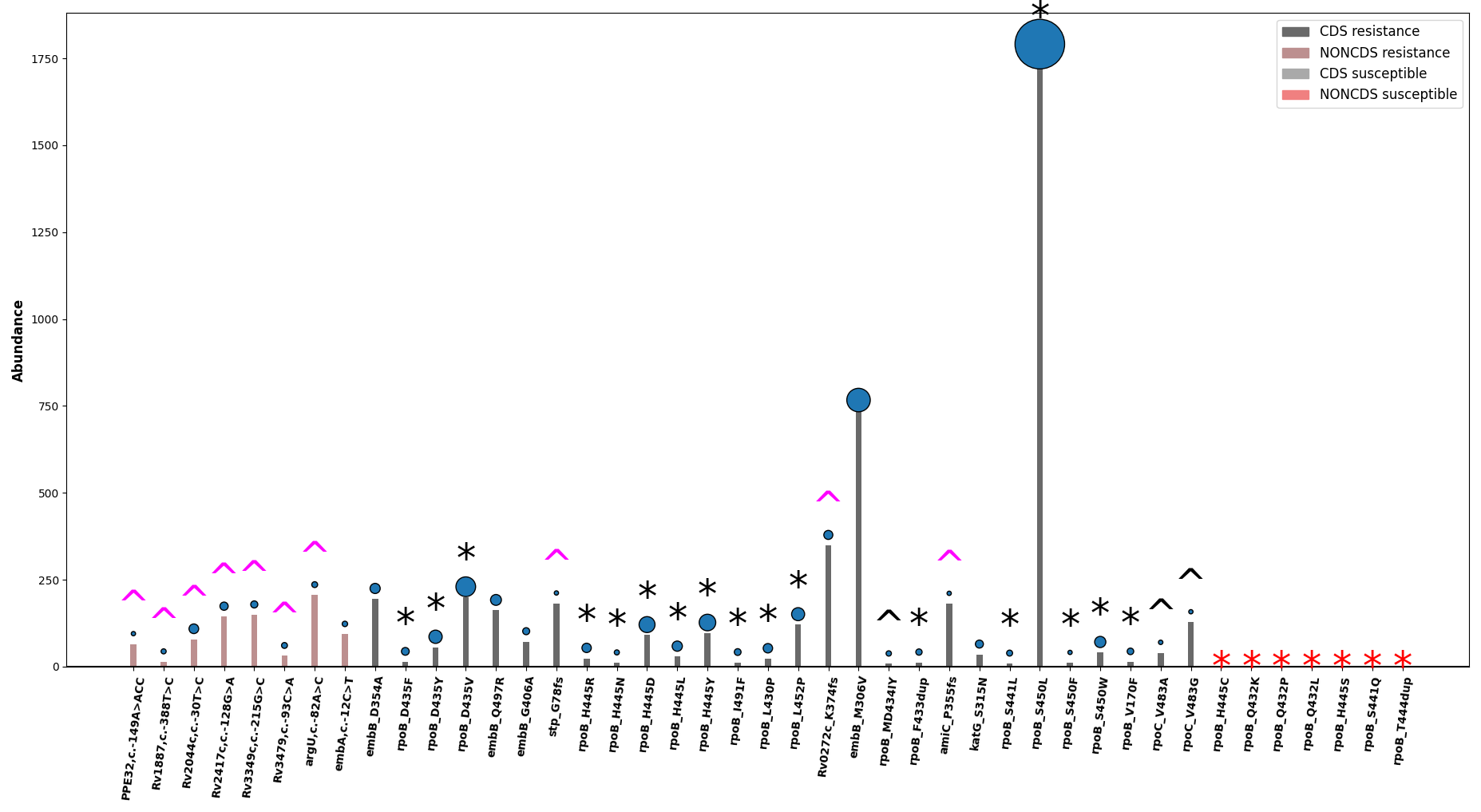
**

**Figure S4C: Rifampicin mutation profile**

**
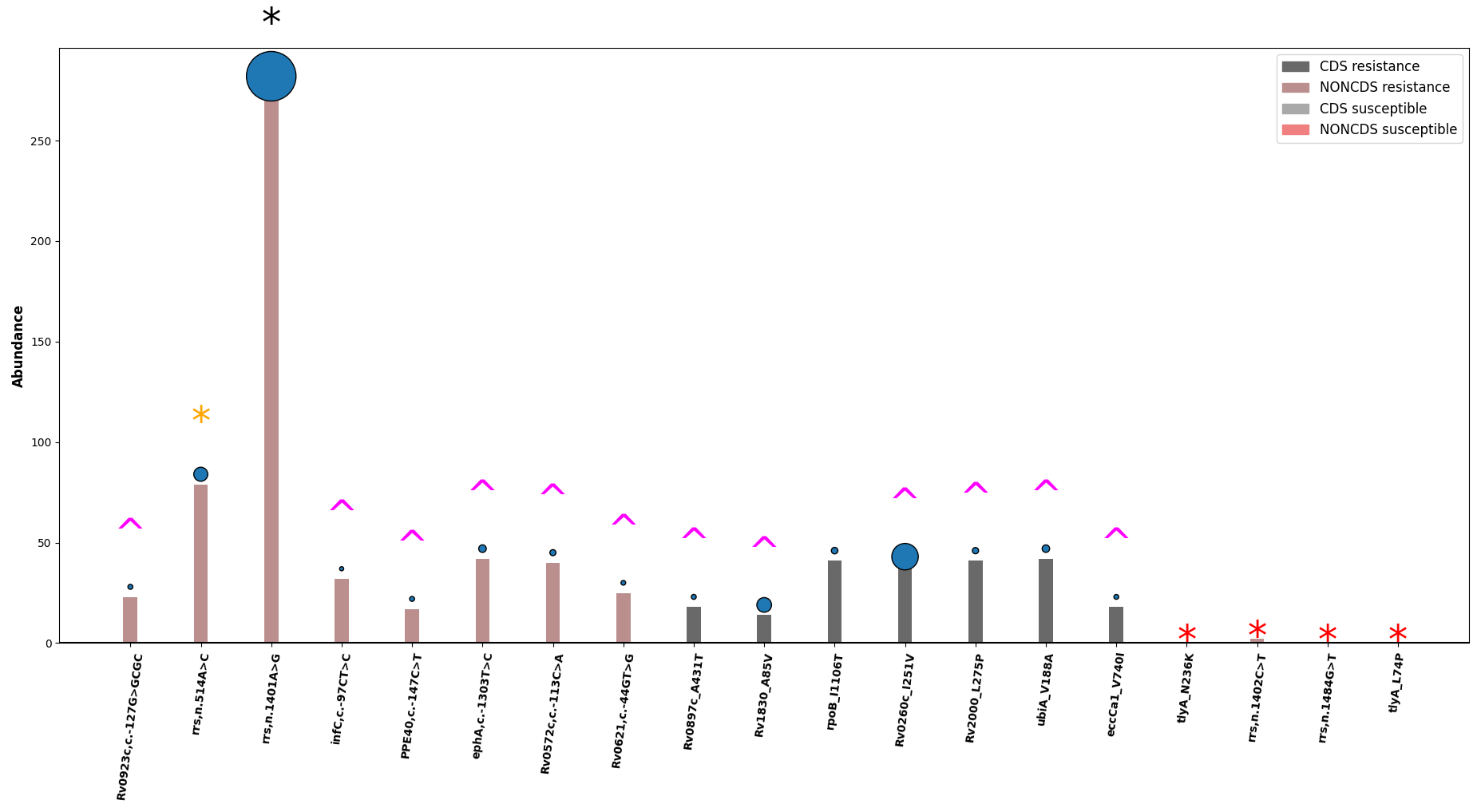
Figure S4D: Capreomycin mutation profile**

**
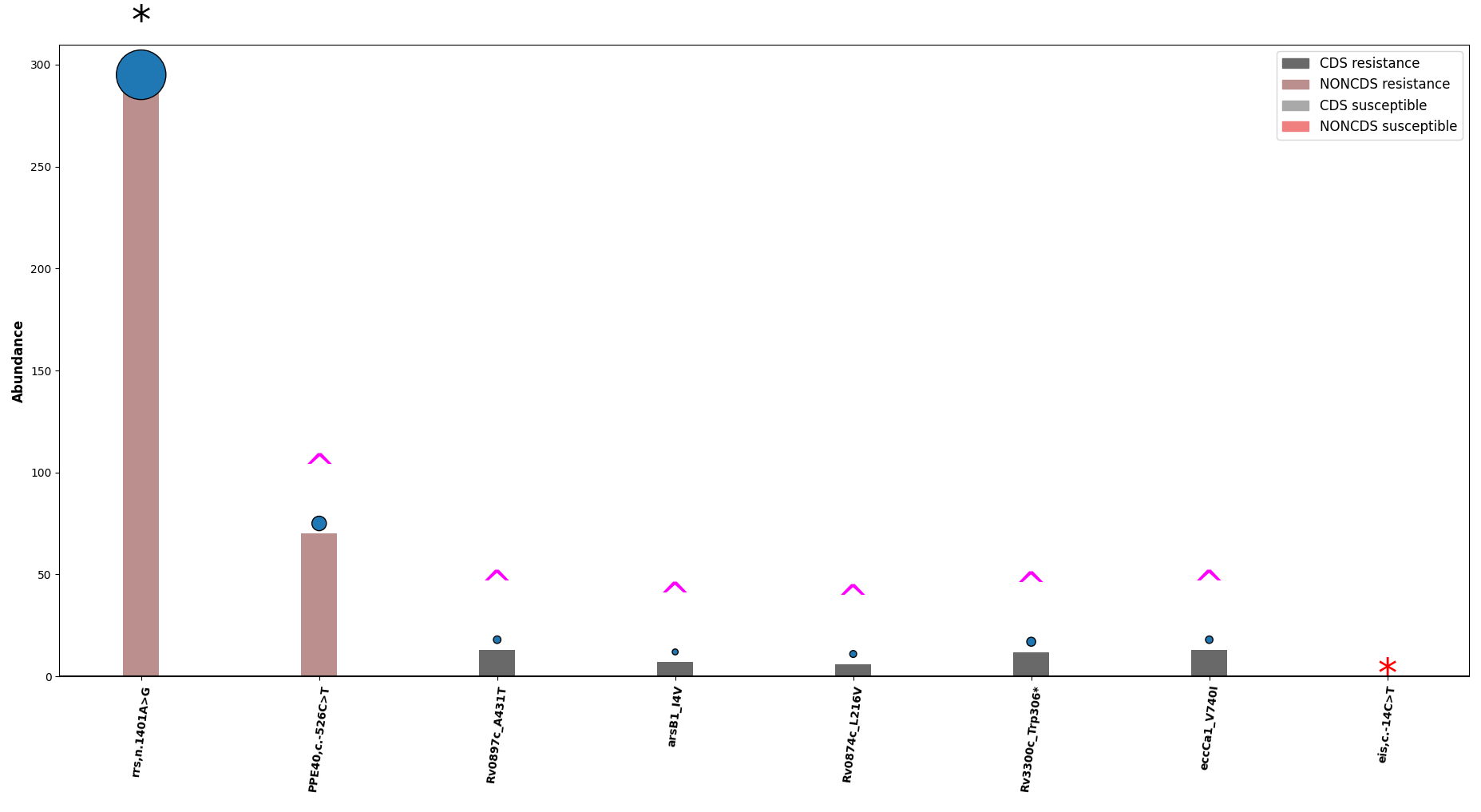
**

**Figure S4E: Amikacin mutation profile**

**
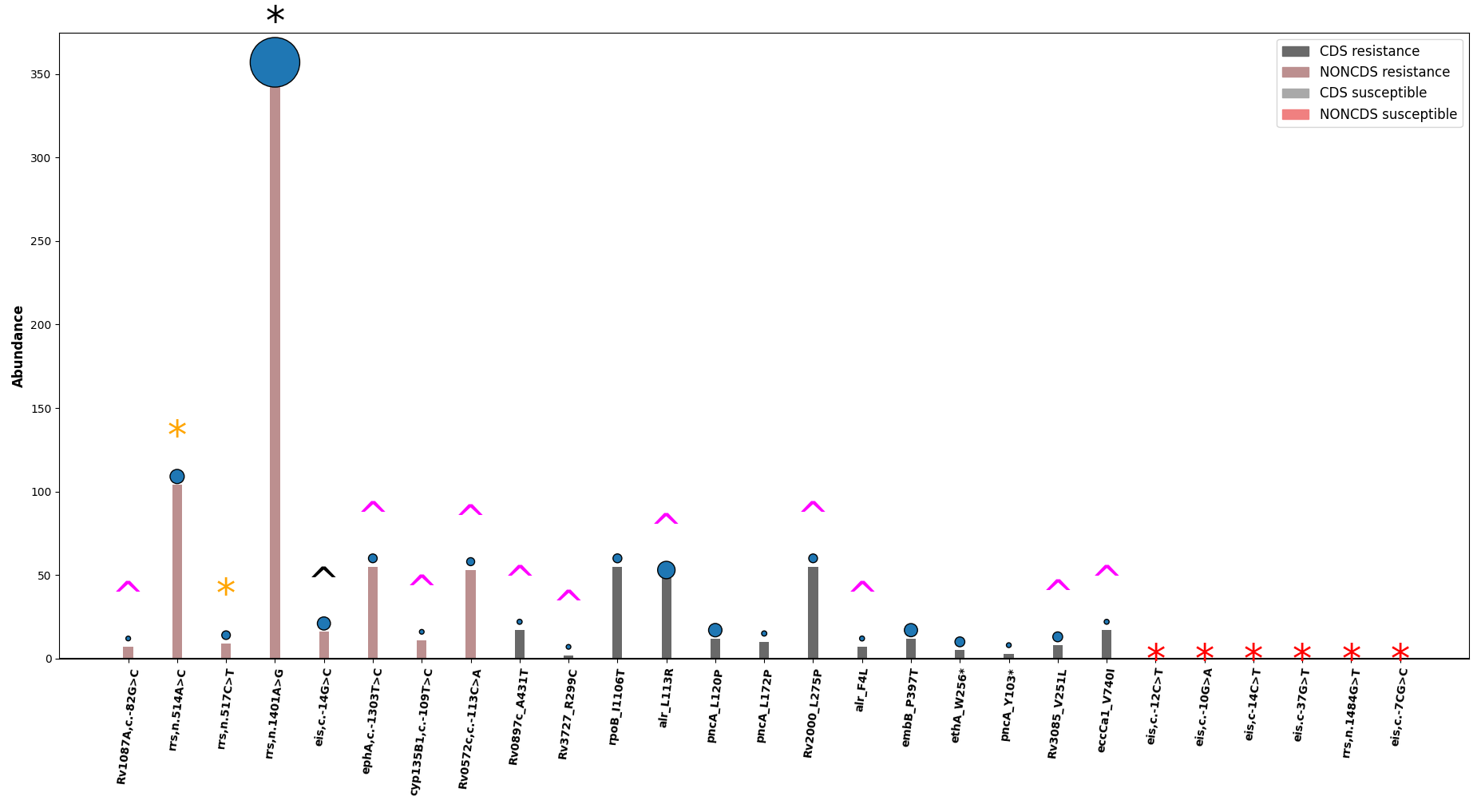
**

**Figure S4F: Kanamycin mutation profile**

**
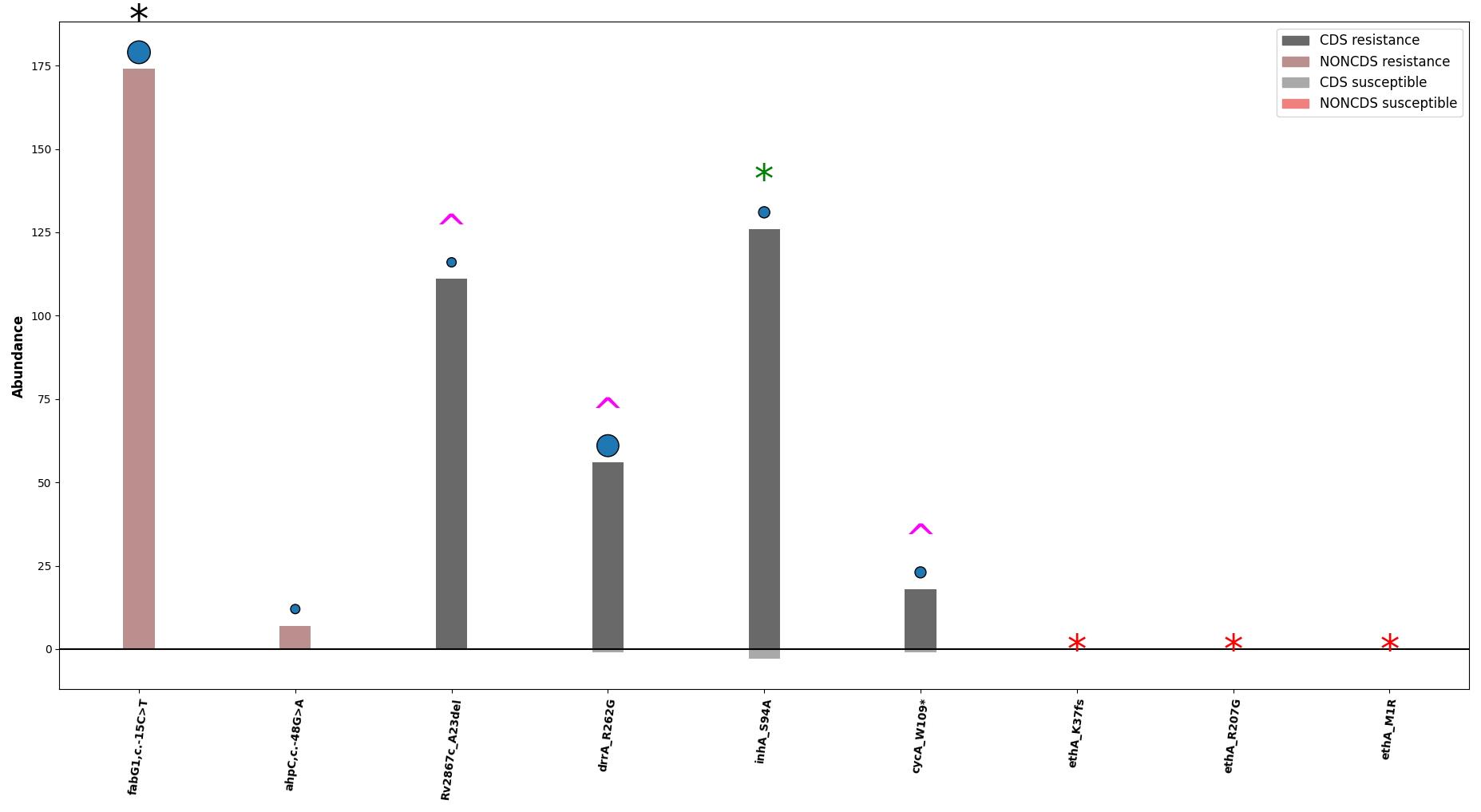
**

**Figure S4G: Ethionamide mutation profile**

**
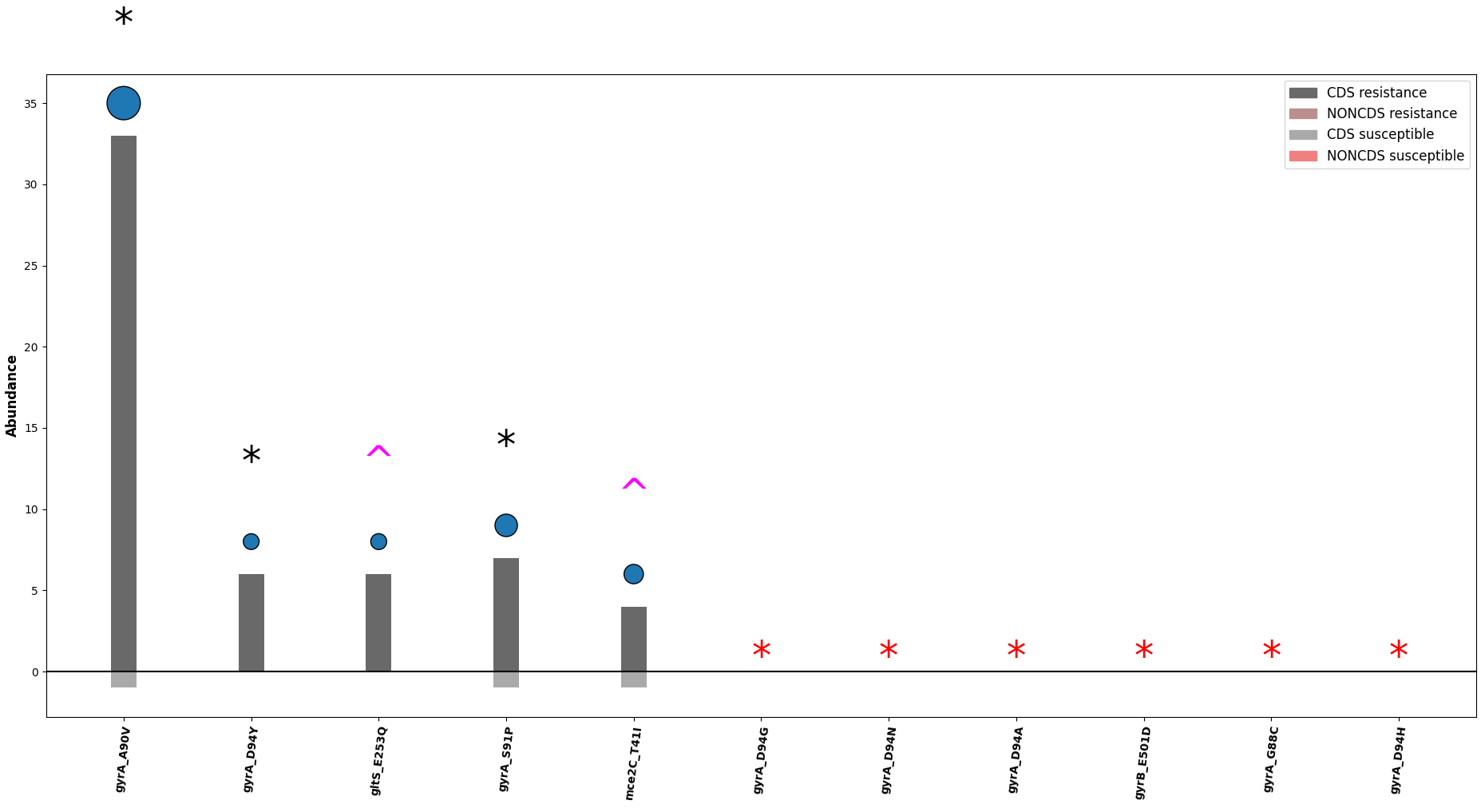
**

**Figure S4H: Moxifloxacin mutation profile**

**
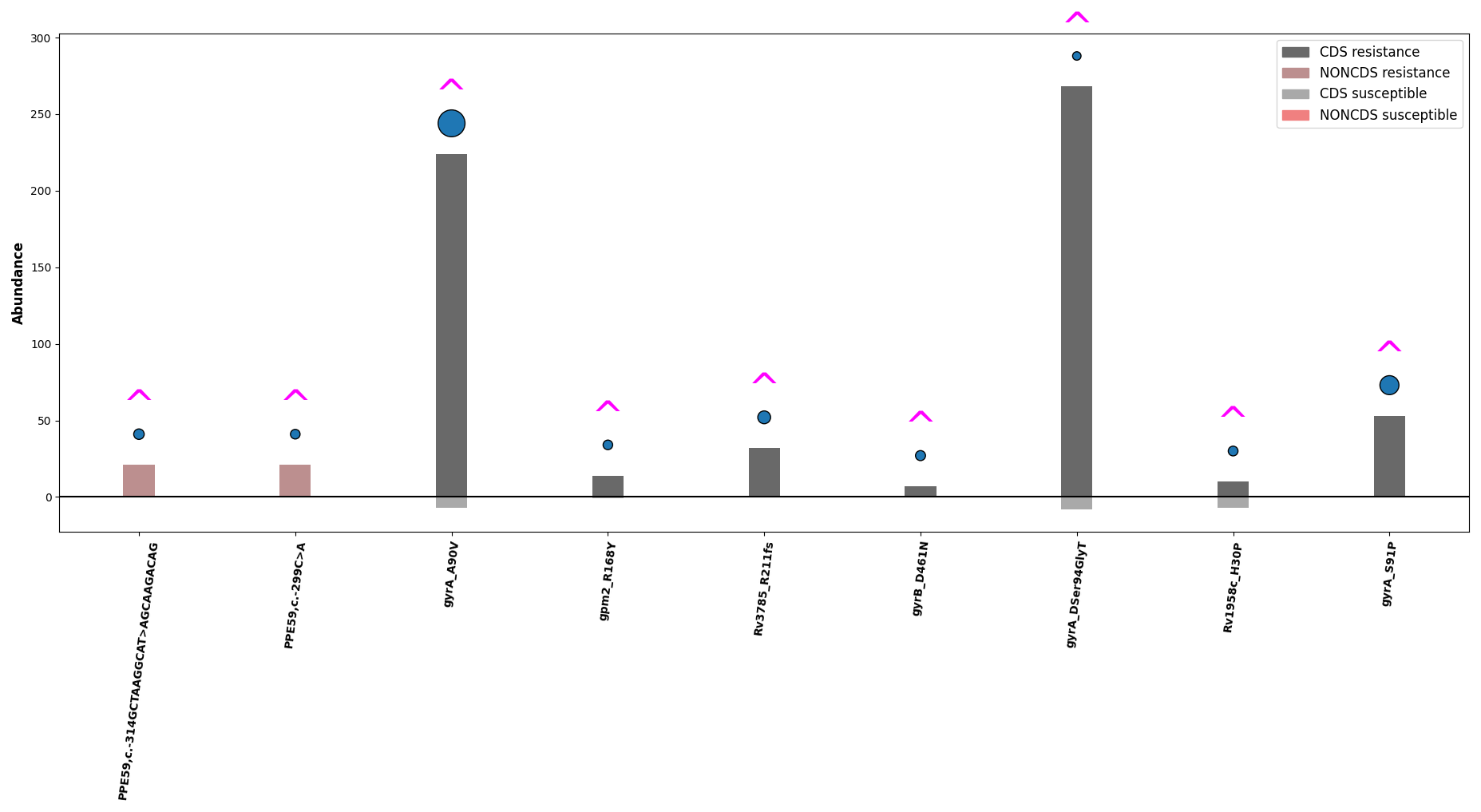
**

**Figure S4I: Ofloxacin mutation profile**

**
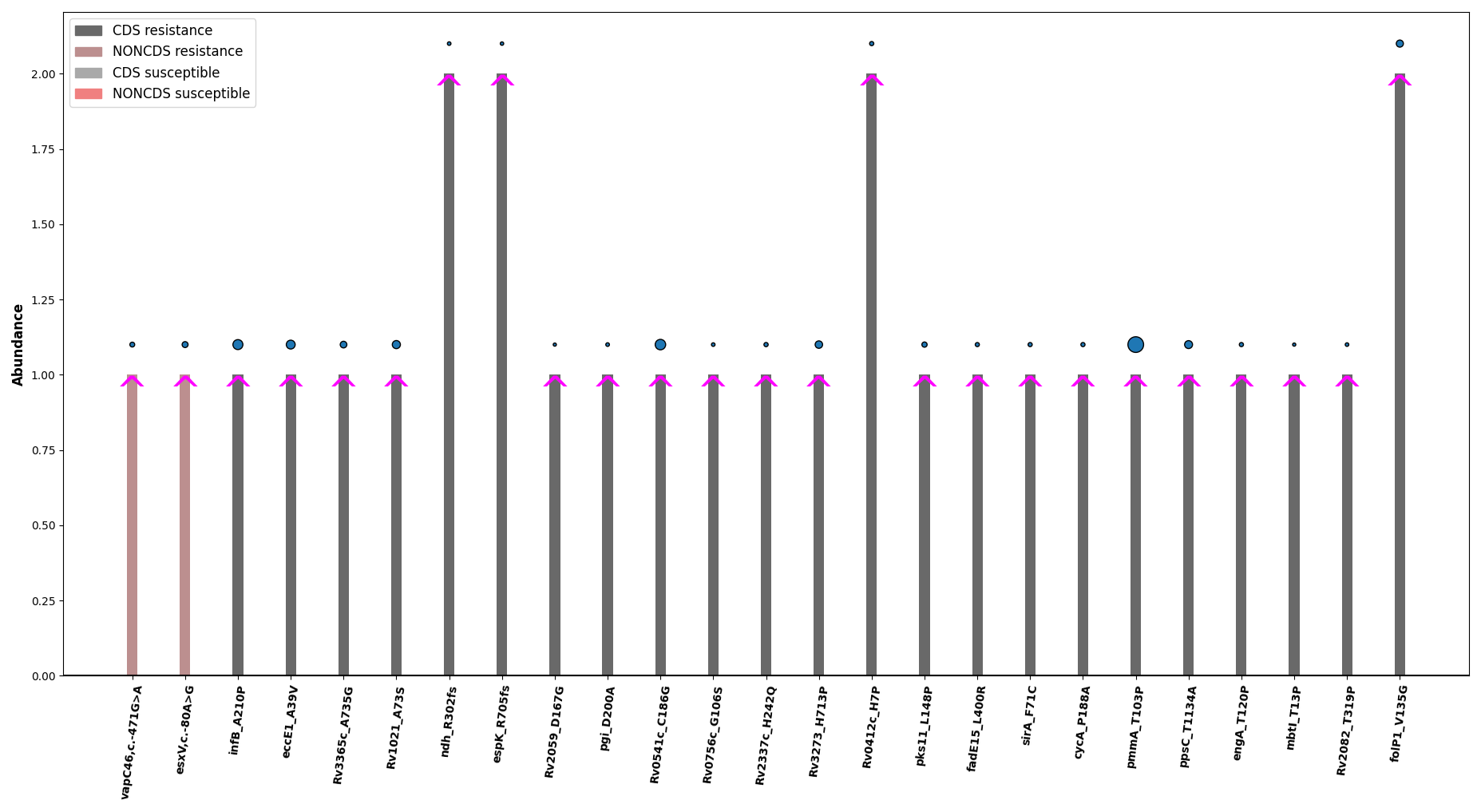
**

**Figure S4J: Cycloserine mutation profile**

**
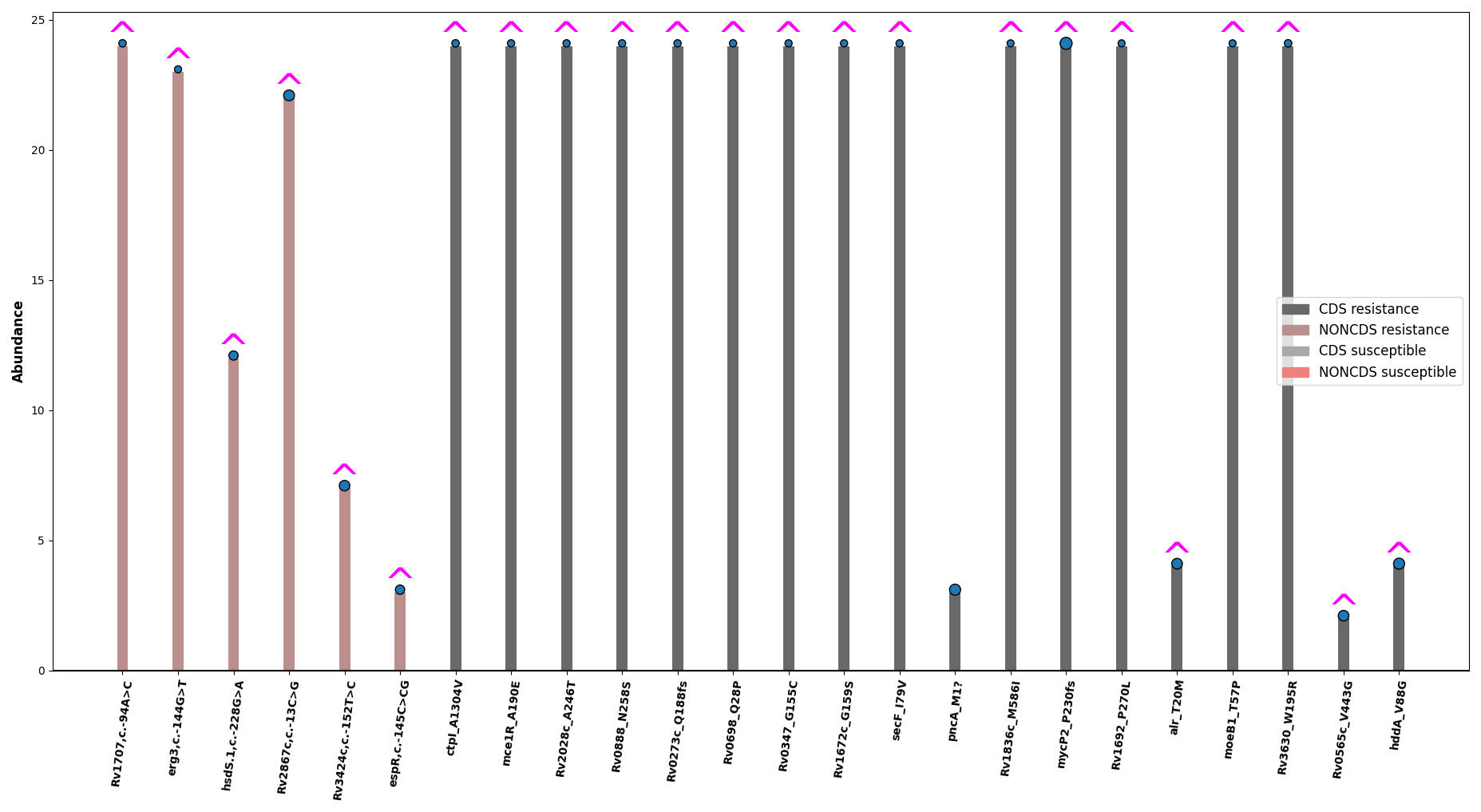
**

**Figure S4K: PAS mutation profile**

**Supplementary Figure S5: Mutation quality analysis in terms of total read depth per base**: a box plot showing y-axis represents total read depth, while the x-axis displays mutations. Figures A and B illustrate known mutations for INH and RIF, respectively, with Figures C and D presenting new mutation in known genes, new gene mutations and co-occurring mutations for the same drugs. Each box plot illustrates: the lower whisker indicates the minimum total read depth, while the upper whisker shows the maximum total read depth. The blue box represents the interquartile range, capturing the central 50% of the data distribution. A yellow line within the box denotes the median value, providing a central tendency measure. A red dashed line at a total read depth of 30 serves as a critical benchmarking threshold to distinguish high-confidence mutations. Points extending beyond the lower and upper whiskers are identified as outlier.

**
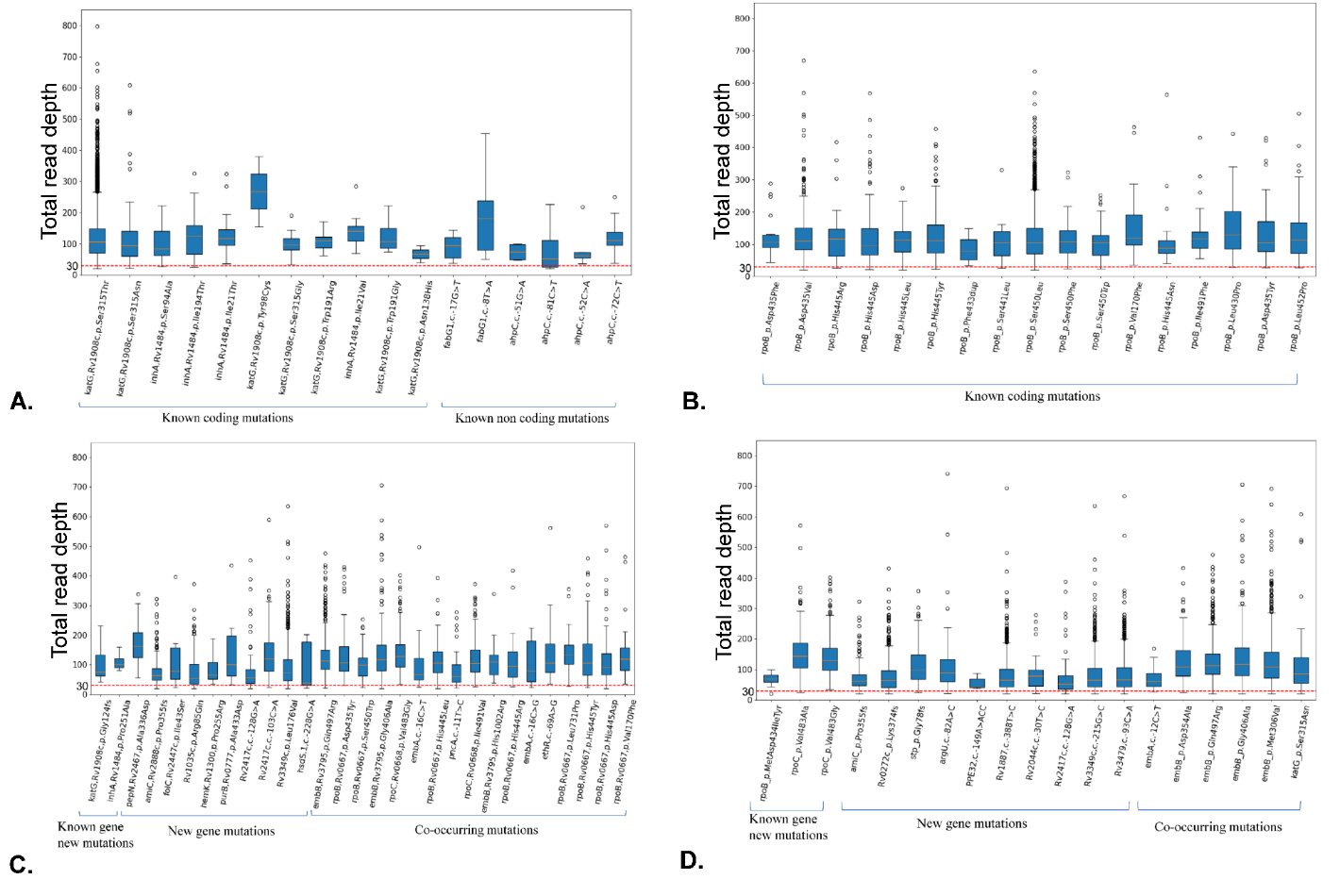
**

**Supplementary Figure S6:** Overview of TB-AMRpred webserver. **A.** The screenshot illustrates the web interface designed for the upload of essential input files required for drug resistance prediction. The input file must consist of the whole genome nucleotide sequence of *M.tb* in either FASTA or FASTQ format. **B.** The screenshot showcases the results of drug resistance prediction for all 13 tubercular drugs.


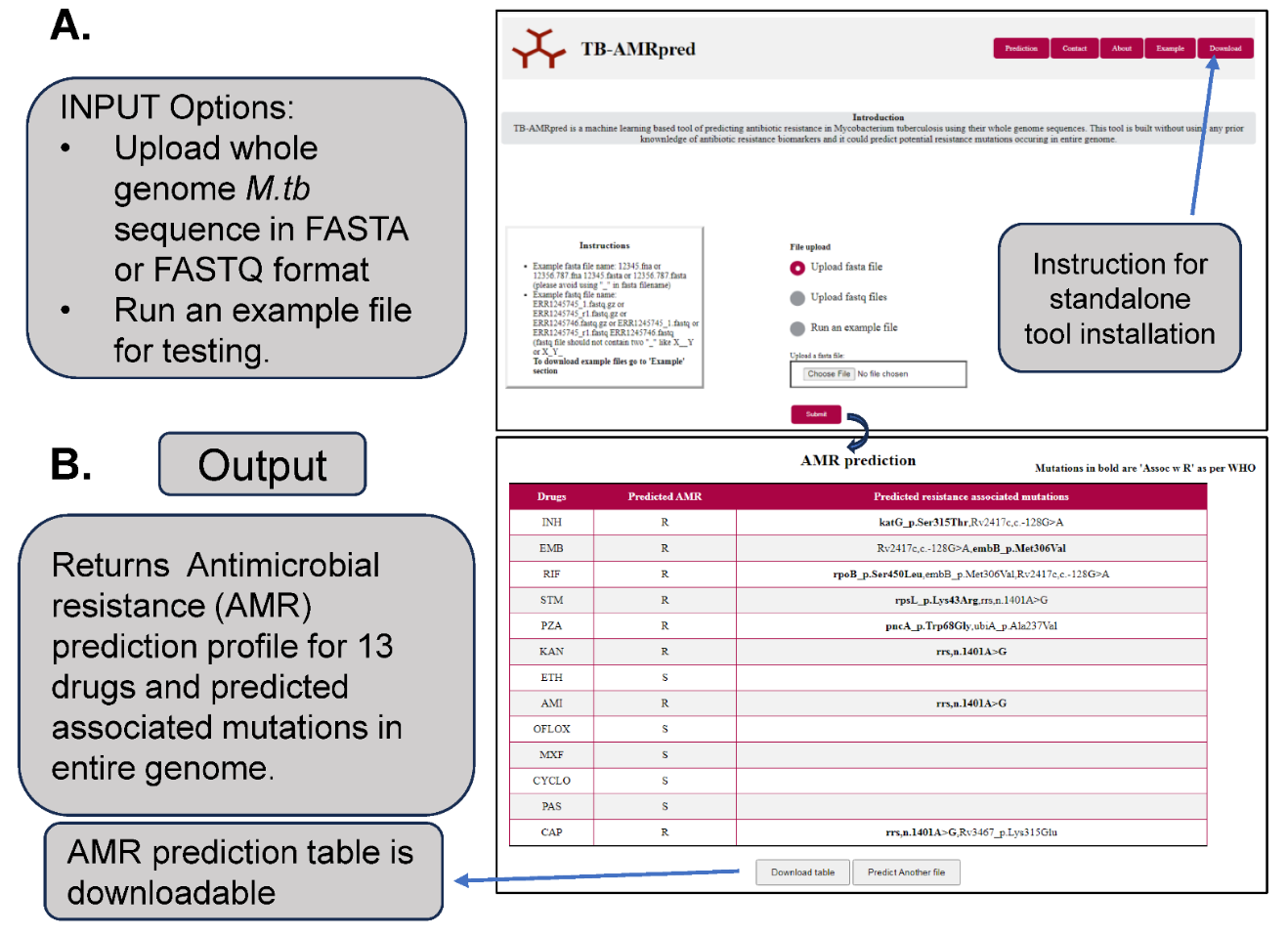

Supplement: vbaf050_Supplementary_Data [file vbaf050_supplementary_data.zip › Supplementary File 2.docx]
